# Ontogenetic variability of the intertympanic sinus distinguishes lineages within Crocodylia

**DOI:** 10.1111/joa.13830

**Published:** 2023-01-29

**Authors:** Gwendal Perrichon, Lionel Hautier, Yohan Pochat‐Cottilloux, Irena Raselli, Céline Salaviale, Benjamin Dailh, Nicolas Rinder, Vincent Fernandez, Jérôme Adrien, Joël Lachambre, Jeremy E. Martin

**Affiliations:** ^1^ CNRS UMR 5276, Université Claude Bernard Lyon 1, ENS de Lyon, Laboratoire de Géologie de Lyon: Terre, Planètes, Environnement Villeurbanne France; ^2^ Institut des Sciences de l'Évolution Université Montpellier, CNRS, IRD, EPHE Montpellier France; ^3^ Mammal Section, Life Sciences, Vertebrate Division The Natural History Museum London UK; ^4^ Geoscience Department, Chemin de Musée 6 University of Fribourg Jurassica Museum Porrentruy Switzerland; ^5^ Imaging and Analysis Centre The Natural History Museum London UK; ^6^ Laboratoire Matériaux, Ingénierie et Science Institut National des Sciences Appliquées de Lyon Villeurbanne France

**Keywords:** 3D geometric morphometrics, Crocodylia, ontogeny, sinus

## Abstract

The phylogenetic relationships within crown Crocodylia remain contentious due to conflicts between molecular and morphological hypotheses. However, morphology‐based datasets are mostly constructed on external characters, overlooking internal structures. Here, we use 3D geometric morphometrics to study the shape of the intertympanic sinus system in crown crocodylians during ontogeny, in order to assess its significance in a taxonomic context. Intertympanic sinus shape was found to be highly correlated with size and modulated by cranial shape during development. Still, adult sinus morphology distinguishes specimens at the family, genus and species level. We observe a clear distinction between Alligatoridae and Longirostres, a separation of different *Crocodylus* species and the subfossil Malagasy genus *Voay*, and a distinction between the *Tomistoma* and *Gavialis* lineages. Our approach is independent of molecular methods but concurs with the molecular topologies. Therefore, sinus characters could add significantly to morphological datasets, offering an alternative viewpoint to resolve problems in crocodylian relationships.

## Graphical abstract

We recognised the usefulness of the paratympanic sinuses in distinguishing major lineages within Crocodylia. 3D geometric morphometric approaches allow us to quantify the ontogeny and variability of the intertympanic sinus within one of the largest dataset of modern species, including one of the largest specimens ever scanned at such a high resolution. We identify internal structures that are clade‐specific and show the potential of those being used in phylogenetic datasets in order to solve major contentions among the Crocodylia, including the long‐standing *Tomistoma*‐*Gavialis* debate.

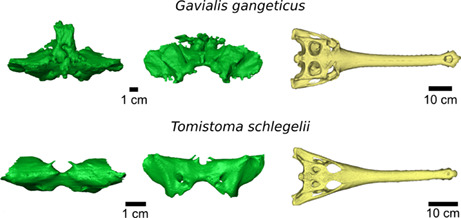

## 1 INTRODUCTION

Modern crocodylians are the living representatives of the order Crocodylia, dating back to the Late Cretaceous period, and constitute one of the two extant archosaurian lineages, along with their closest relatives, Aves. Nowadays, the diversity of the crown group is represented by semi‐aquatic ambush predators dwelling under intertropical climates, comprising nine genera and 23–28 species determined morphologically and/or molecularly (Grigg & Kirshner, 2015). Their phylogenetic relationships have been traditionally explored mostly through the lens of external morphology (Brochu, 1997, 1999, 2000, 2001; Groh et al., 2019; Rio & Mannion, 2021). Molecular phylogenies, using both mitochondrial and nuclear DNA, have often resulted in conflicting results with the morphological methods regarding relationships between and within the three extant families (Hekkala et al., 2021; Janke et al., 2005; Meredith et al., 2011; Milián‐García et al., 2020; Oaks & Dudley, 2011; Pan et al., 2021; Poe, 1996; Roos et al., 2007; Willis et al., 2007). Differences between molecular and most morphological topologies are mainly centred around the phylogenetic position of Gavialidae, and the *Gavialis—Tomistoma* debate, a case study discussed within the frame of convergence towards longirostry (Brochu, 2003; Gatesy et al., 2003; Groh et al., 2019; Lee & Yates, 2018; Rio & Mannion, 2021).

Crocodylomorpha and their pseudosuchian stem‐groups stand out in being characterised by an expanded set of paratympanic sinuses, a system of endocranial pneumatic cavities linked to the auditory system and the pharynx, invading the braincase bones (Dufeau & Witmer, 2015; Kuzmin et al., 2021). Among mammals and archosaurs, previous studies have investigated the use of endocranial sinuses to resolve ecological, phylogenetic or biomechanical questions (Witmer, 1999). The shape of such structures has often been revealed to be correlated with the development of cranial features (Curtis et al., 2015; Farke, 2010; Ito & Nishimura, 2016). Sinus shape has also been proposed to be linked to cranial stress dissipation (Sharp & Rich, 2016), and shape differentiation has been observed between phylogenetic groupings or biogeographically distinct populations (Billet et al., 2017; Curtis et al., 2015; Curtis & Van Valkenburgh, 2014; Farke, 2010; Rossie, 2008). Sinus morphology also seems to be impacted by the living environment, showing substantial volume reduction in tetrapod lineages that underwent land to marine environment transition (Brusatte et al., 2016; Cowgill et al., 2021; Curtis et al., 2015; Fernández & Herrera, 2021). The potential relevance of these structures for systematics and ecological studies thus deserves attention in crocodylian species, with some fossil lineages showing debated phylogenetic positions or undergoing drastic habitat transitions (e.g. Thalattosuchia, Dyrosauridae, Sebecidae).

The complex morphologies of the crocodylian paratympanic sinuses were initially described on the basis of dissections and mechanical tomography (Colbert, 1946; Owen, 1850; Tarsitano, 1985; Van Beneden, 1882). The recent development of non‐invasive analytical X‐ray micro‐computed tomography techniques (μCT) has facilitated further investigation of crocodylian cranial anatomy: thus, paratympanic structures have recently received renewed interest and resulted in recent revisions to traditional sinus nomenclature (Dufeau & Witmer, 2015; Kuzmin et al., 2021). In the last decade, many papers have examined the neuroanatomy of fossil crocodylomorphs, pointing out that such structures may be important for inferring ecological and evolutionary patterns (Bona et al., 2015; Brusatte et al., 2016; Erb & Turner, 2021; Martin et al., 2022; Pochat‐Cottilloux et al., 2021; Serrano‐Martínez et al., 2021; Serrano‐Martínez, Knoll, Narváez, et al., 2019; Serrano‐Martínez, Knoll, Narváez, et al., 2019). Therefore, as these approaches are multiplying, it has become timely to get an as complete as possible perspective on the morphological variability of these structures studied across extant crocodylian lineages. Among extant Crocodylia, a few studies already highlighted several endocranial and sinus structures as an additional proxy to refine the taxonomic determination of extant and fossil specimens, then proposed their potential use in phylogenies (Kuzmin et al., 2021; Martin et al., 2022).

Furthermore, several works have shown that the crocodylian skull and endocranial system are heavily modified during post‐hatching development (Dufeau & Witmer, 2015; Hu et al., 2021; Kuzmin et al., 2021; Lessner et al., 2022; Tarsitano, 1985). Cranial morphology is plastic across ontogeny, especially regarding the shape of the snout, which is the most variable skull region in extant species. The shape of the braincase, containing the endocranial organs, also displays important modifications during development such as verticalisation, skull table flattening, quadrate extension and enlargement of temporal fenestrae (Cossette et al., 2021; Morris et al., 2019, 2021; Piras et al., 2010, 2014). Assessing the ontogenetic changes in the paratympanic sinuses from a developmental point of view is thus necessary to discuss their morphology and evolution.

**FIGURE 1:**
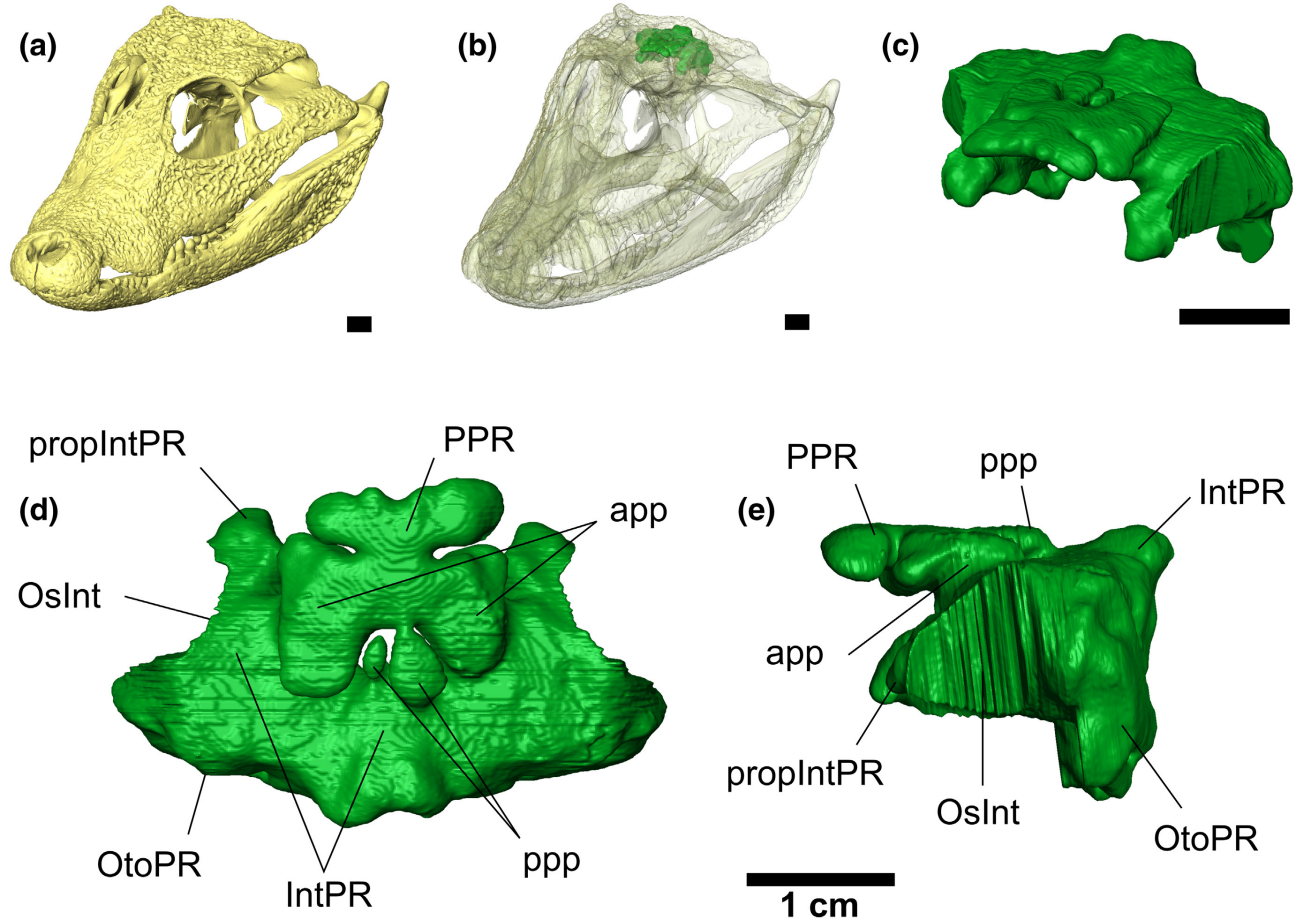
Intertympanic sinus system of *Osteolaemus tetraspis* (MHNM 9095.0). (a) Oblique view of the skull; (b) semi‐transparent view of the skull showing the position of the intertympanic sinus system; (c) oblique, (d) dorsal, (e) left lateral views of the intertympanic sinus system. app, anterolateral pre‐parietal process; IntPR, intertympanic pneumatic recess; OsInt, ostium between intertympanic pneumatic recess and middle ear; OtoPR, otoccipital pneumatic recess; ppp, posteromedial pre‐parietal process; PPR, parietal pneumatic recess; PropIntPR, prootic part of intertympanic pneumatic recess. Scale bars: 1 cm.

In the present paper, we focus specifically on the morphological variability of the intertympanic sinus system (Figure 1), a part of the paratympanic sinuses that displays noticeable differences across extant crocodylian species. The aim of this study was to provide a detailed description of the ontogeny of the intertympanic sinus and associated diverticula in extant crocodylian species, to establish an overview of its variability and provide new tools for taxonomic identification and future phylogenetic analyses. We use 3D geometric morphometric quantification to assess the morphological variations of the intertympanic sinus system in 17 modern and two subfossil species (*Voay robustus* and *Crocodylus* sp.), reconstruct ontogenetic trajectories and discuss morphological features regarding the crocodylian phylogenetic framework.

## 2 MATERIAL AND METHODS

### 2.1 Specimen sampling

The braincases of 64 extant specimens were examined for 3D reconstructions of their endocranial structures (Table 1). Our sample comprises 17 extant species, representing all but one (*Paleosuchus*) of the extant genera currently recognised: *Alligator*, *Caiman*, *Melanosuchus*, *Crocodylus*, *Osteolaemus*, *Mecistops*, *Gavialis* and *Tomistoma*. Our dataset includes hatchlings, juveniles, sub‐adults and adults to account for ontogenetic changes, especially in *Alligator*, *Caiman*, *Crocodylus* and *Gavialis* for which we had enough specimens to describe ontogenetic series (Table 1). All species but *Caiman yacare*, *Melanosuchus niger*, *Crocodylus acutus*, *Crocodylus halli* and *Crocodylus palustris*, are represented by at least two adult or sub‐adult specimens. All specimens were retrieved from museum collections or online databases and no living specimens were used or euthanised for the purpose of this study.

**TABLE 1:** List of extant specimens studied along with their total skull length and ontogenetic stage.

Species	ID number	Skull length (cm)	Ontogenetic stage	Snout shape
*Alligator mississippiensis*	OUVC 9761	30.35	Adult	Brevirostrine
*Alligator mississippiensis*	UCBL WB35	26	Sub‐adult	
*Alligator mississippiensis*	TMM M‐983	17.46	Sub‐adult	
*Alligator mississippiensis*	SMNK‐REP 308	12.32	Juvenile	
*Alligator mississippiensis*	SMNK‐REP 309	10.97	Juvenile	
*Alligator mississippiensis*	OUVC 11415	9.52	Juvenile	
*Alligator mississippiensis*	SMNK‐REP 311	8.57	Juvenile	
*Alligator mississippiensis*	SMNK‐REP 164	3.7	Hatchling	
*Alligator mississippiensis*	OUVC 10606	2.94	Hatchling	
*Caiman crocodilus*	UMMZ herps 128024	21.91	Adult	Brevirostrine
*Caiman crocodilus*	UMMZ herps 46112	18.12	Adult	
*Caiman crocodilus*	UMMZ herps 155282	10.08	Juvenile	
*Caiman latirostris*	UMMZ herps 155287	21.84	Adult	Brevirostrine
*Caiman latirostris*	UMMZ herps 155286	15.65	Adult	
*Caiman latirostris*	UMMZ herps 155288	15.33	Adult	
*Caiman latirostris*	UMMZ herps 155283	13.4	Sub‐adult	
*Caiman latirostris*	UMMZ herps 155285	10.1	Sub‐adult	
*Caiman latirostris*	UMMZ herps 155284	7.6	Juvenile	
*Caiman latirostris*	SMNK‐REP 315	3.22	Hatchling	
*Caiman latirostris*	SMNK‐REP 317	3.2	Hatchling	
*Caiman latirostris*	SMNK‐REP 316	3.19	Hatchling	
*Caiman latirostris*	SMNK‐REP 314	3.06	Hatchling	
*Caiman yacare*	UMMZ herps 155289	30.14	Adult	Brevirostrine
*Crocodylus acutus*	MZS Cro 055	52.5	Adult	Mesorostrine
*Crocodylus halli*	UF herp 145927	28.44	Sub‐adult	Mesorostrine
*Crocodylus niloticus*	MHNL 50001405	61	Adult	Mesorostrine
*Crocodylus niloticus*	MHNL 50001387	45.2	Adult	
*Crocodylus niloticus*	MHNL 50001397	45	Adult	
*Crocodylus niloticus*	MHNL 50001388	42	Adult	
*Crocodylus niloticus*	UM 2001‐1756‐1‐434NR	21.07	Sub‐adult	
*Crocodylus niloticus/suchus*	MHNL 90001855	13.4	Juvenile	
*Crocodylus niloticus/suchus*	MHNL 90001850	12.5	Juvenile	
*Crocodylus niloticus/suchus*	MHNL 90001851	10.12	Juvenile	
*Crocodylus niloticus*	ag SVSTUA 022002	5.78	Hatchling	
*Crocodylus palustris*	MHNL 50001398	47.5	Adult	Mesorostrine
*Crocodylus* cf *porosus*	UCBL 2019‐1‐237	34.5	Adult	Mesorostrine
*Crocodylus porosus*	OUVC 10899	9	Juvenile	
*Crocodylus rhombifer*	MHNL 42006507	23.43	Sub‐adult	Mesorostrine
*Crocodylus rhombifer*	MHNL 42006506	21.8	Sub‐adult	
*Crocodylus siamensis*	UCBL WB41	47.2	Adult	Mesorostrine
*Crocodylus siamensis*	MHNL 50001389	38.3	Adult	
*Crocodylus* sp.	UCBL‐FSL 532077	19.1	Sub‐adult	Mesorostrine
*Gavialis gangeticus*	MHNL 50001407	68.4	Adult	Longirostrine
*Gavialis gangeticus*	UF herp 118998	51.69	Adult	
*Gavialis gangeticus*	NHMUK 1873	45	Adult	
*Gavialis gangeticus*	NHMUK 1846.1.7.3	27.5	Sub‐adult	
*Gavialis gangeticus*	YPM herr 008438	8.15	Hatchling	
*Mecistops* sp.	NHMUK 1924.5.10.1	62	Adult	Longirostrine
*Mecistops* sp.	UM N89	49	Adult	
*Mecistops* sp.	AMU Zoo‐04721	39.4	Sub‐adult	
*Mecistops* sp.	MZS Cro083	35	Sub‐adult	
*Mecistops* sp.	MHNL 50001393	31.4	Sub‐adult	
*Mecistops* sp.	ag SVSTUA 022001	29.47	Sub‐adult	
*Melanosuchus niger*	MZS Cro073	37.8	Adult	Brevirostrine
*Osteolaemus tetraspis*	UCBL 2019‐1‐236	24.1	Adult	Brevirostrine
*Osteolaemus tetraspis*	MZS Cro040	19.2	Adult	
*Osteolaemus tetraspis*	NHMUK 1862.6.30.5	17.5	Adult	
*Osteolaemus tetraspis*	MHNM 9095.0	16	Adult	
*Osteolaemus tetraspis*	FMNH 98,936	7.81	Juvenile	
*Tomistoma schlegelii*	NHMUK 1893.3.6.14	52.1	Adult	Longirostrine
*Tomistoma schlegelii*	MZS Cro094	48.5	Adult	
*Tomistoma schlegelii*	TMM M‐6342	33.55	Sub‐adult	
*Tomistoma schlegelii*	UM1097	20.73	Juvenile	
*Tomistoma schlegelii*	FMNH 98874	13.54	Juvenile	

Three specimens of the subfossil species *V. robustus* from the Holocene of Madagascar were included in the analyses along with extant crocodylian species, as this genus was still present until less than 2000 years cal BP and was contemporaneous of the genus *Crocodylus* on the island (Hekkala et al., 2021; Martin et al., 2022). Two subfossil specimens of *Crocodylus* sp. (MHNL QV14 and MNHN F.1908.5‐2) from the Holocene of Madagascar (Martin et al., 2022) were also included in the study (Table 2).

**TABLE 2:** List of fossil specimens studied along with their braincase width and ontogenetic stage.

Species	ID number	Age	Locality	Braincase width (cm)	Inferred ontogenetic stage
*Crocodylus* sp.	MNHN F.1908.5–2	Holocene	Madagascar	15	Adult
*Crocodylus* sp.	MHNL QV14	7670‐7510 cal BP	Madagascar	11.5	Adult
*Voay robustus*	NHMUK PV R 36685	Holocene	Madagascar	12.5	Adult
*Voay robustus*	NHMUK PV R 36684	Holocene	Madagascar	11.76	Adult
*Voay robustus*	MNHN F.1908.5	Holocene	Madagascar	11.25	Adult

### 2.2 X‐ray micro‐computed tomography

Thirty specimens were scanned at the Laboratoire Mateis (INSA), using a V|tome|X CT instrument (GE Sensing & Inspection Technologies Phoenix X‐Ray) for skulls smaller than 50 cm, and a DTHE (Double Tomographe Haute Energie by RX Solutions) for larger skulls. One specimen was scanned at MNHN Viscom France; two at the ISEM (RX Solutions EasyTom 150); the eight specimens from the SMNK were scanned at the KIT IPS and seven were scanned at the NHMUK (Nikon Metrology XT H 225 ST). The rest of the specimens comes from CT data available in the online databases Morphosource (https://www.morphosource.org/) and Digimorph (http://www.digimorph.org/). Additional information on the μCT parameters, including voxel size, can be found in Data S1.

### 2.3 Image processing

Raw CT data were imported into the software ImageJ (Schneider et al., 2012), where 16‐bit image stacks were converted into 8‐bit to reduce file weight and contrast was enhanced between cranial bones and cavities. The resulting files were imported into the software Avizo Lite (version 7, 8.1, 9 and 9.5) for digital segmentation, volume rendering and visualisation of the endocranial structures. Segmentation was performed by multiple authors, and anatomical descriptions are outlined in Section 3.1. It was either done semi‐automatically with inter‐slice interpolation for most extant specimens, or manually slice by slice for subfossil specimens due to the sediment filling. We follow the nomenclature of Kuzmin et al. (2021) (and references therein) regarding endocranial terminology. Due to the size limitations of the Phoenix instrument, μCT acquisition of large specimens was, in this case, restricted to the basicranium: metrical measurements were thus obtained on the complete skulls when they were available. The most recent μCT acquisitions were conducted on the DTHE (INSA Lyon), where size limitations were no longer a problem, allowing for the largest skulls in our database to be μCT‐scanned entirely. Total skull length (SL) was measured from the anterior margin of the premaxillae to the posterior margin of the supraoccipital. All volume renderings are available on MorphoMuseum (https://www.morphomuseum.com/) for 3D visualisation (Perrichon et al., 2022).

### 2.4 Ontogenetic stage determination

Crocodylians undergo continuous growth during development, precluding a clear osteological assessment of ontogenetic stages and making it difficult to attribute precise size boundaries which would also differ for each species (Morris et al., 2019; Schwab et al., 2021). Therefore, extant specimens were classified into four size classes (hatchling, juvenile, sub‐adult and adult) based on their total SL and respective genus (see Table 1, Table S1), in order to compare sinus shape between specimens of different sizes. Our dataset includes eight hatchlings, 13 juveniles, 14 sub‐adults and 34 adults (Table 1). As rostrum size is greatly variable between species, and as the development of each individual of a species can also be different depending on food access and environmental condition, we also provide the skull width (SW) for each specimen (or the braincase width for incomplete fossils) in addition to the inferred ontogenetic stage when addressing a specific individual. This is because this size proxy is less influenced by rostrum length than is SL (O'Brien et al., 2019).

### 2.5 Taxonomical issues

Uncertainty over taxonomic distributions of museum specimens can be a problem, especially in the genus *Crocodylus* where determination at the species level is difficult for juvenile and sub‐adult specimens. Indeed, the differences between species of this genus often become clearly visible only in sub‐adults or adults, while some relevant post‐cranial characters are often missing. *Crocodylus siamensis* and *C. porosus* are especially difficult to distinguish, and museum identifications concerning these two species were often erroneous due to their convergent cranial characters; as such, specimens of these two species were considered as a same entity for the linear regressions, pending further work on the precise morphological delineation between these two species.

The case of *Crocodylus niloticus* is also particularly troubling because this ‘morphological’ species seems to be paraphyletic according to molecular phylogenies based on mitochondrial and nuclear DNA (Hekkala et al., 2011; Meredith et al., 2011; Pan et al., 2021). The ‘Eastern’ African populations are more closely related to the other *Crocodylus* species while the ‘Western’ African populations lie at the base of the phylogenetic relationships of the genus and represent the cryptic species *Crocodylus suchus* (Nicolaï & Matzke, 2019). However, as those populations are morphologically undistinguishable for now without information on their geographical origin (Nicolaï & Matzke, 2019), the Egyptian embalmed mummies (MHNL 90001850, 90001851, 90001855) could correspond to either *C. niloticus* or *C. suchus*.

### 2.6 Landmark protocol

**FIGURE 2:**
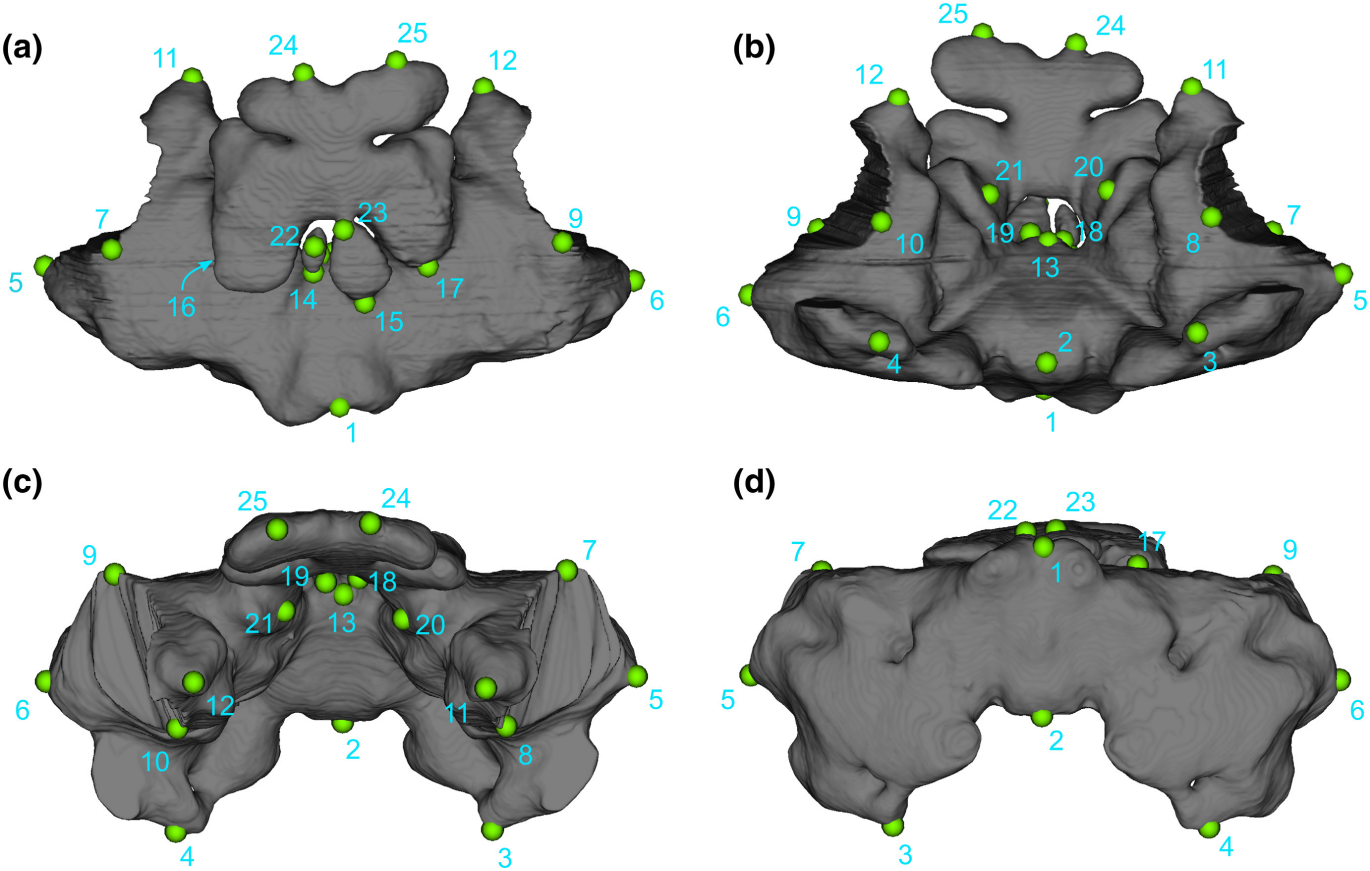
Location of the 3D landmarks captured on the specimens. (a) Dorsal view, (b) ventral view, (c) anterior view and (d) posterior view of the intertympanic sinus of *Osteolaemus tetraspis* (MNHM 9095.0). Landmarks definitions are given in Table 3.

The 3D mesh models of the intertympanic sinus system were exported from the software Avizo as STL files, to be quantified using a 3D geometric morphometric approach. Twenty‐five type II landmarks were placed on the surface of the 3D models using the software MorphoDig 1.6.4 (Lebrun, 2018). The landmarks were chosen to reflect the shape and extension of the pneumatic cavities and were placed on the maximal curvatures and maximum extensions of the different structures (Figure 2, Table 3). Using this so‐called ‘homology‐free’ method enables us to compare shape even if a given structure is seemingly absent in a given specimen, by using partly degenerate configurations of landmarks: that is, when a structure is extremely reduced or absent, all landmarks characterising it are placed in the same location, following Polly (2008) and Klingenberg (2008).

**TABLE 3:** Landmark definitions.

Number	Definition of landmarks
1	Maximum posterodorsal flexure of medial part of intertympanic recess
2	Maximum ventral extension of medial part of intertympanic recess
3	Maximum ventral extension of left otoccipital recess
4	Maximum ventral extension of right otoccipital recess
5	Maximum lateral extension of left otoccipital recess
6	Maximum lateral extension of right otoccipital recess
7	Maximum dorsal extension of left ostium between intertympanic recess and pharyngotympanic recess
8	Maximum ventral extension of left ostium between intertympanic recess and pharyngotympanic recess
9	Maximum dorsal extension of right ostium between intertympanic recess and pharyngotympanic recess
10	Maximum ventral extension of right ostium between intertympanic recess and pharyngotympanic recess
11	Maximum anterior extension of left prootic part of intertympanic recess
12	Maximum anterior extension of right prootic part of intertympanic recess
13	Maximum anterior extension of medial part of intertympanic recess
14	Maximum dorsal flexure of contact between left posterior pre‐parietal process and intertympanic recess
15	Maximum dorsal flexure of contact between right posterior pre‐parietal process and intertympanic recess
16	Maximum dorsal flexure of contact between left anterior pre‐parietal process and intertympanic recess
17	Maximum dorsal flexure of contact between right anterior pre‐parietal process and intertympanic recess
18	Maximum ventral flexure of contact between left posterior pre‐parietal process and intertympanic recess
19	Maximum ventral flexure of contact between right posterior pre‐parietal process and intertympanic recess
20	Maximum ventral flexure of contact between left anterior pre‐parietal process and intertympanic recess
21	Maximum ventral flexure of contact between right anterior pre‐parietal process and intertympanic recess
22	Maximum dorsal extension of left posterior pre‐parietal process
23	Maximum dorsal extension of right posterior pre‐parietal process
24	Maximum anterior extension of left anterior pre‐parietal process
25	Maximum anterior extension of right anterior pre‐parietal process

The two specimens of *Crocodylus rhombifer* (MHNL 42006506 and 42006507) were too incomplete to be quantified with the complete set of landmarks. They were only used for descriptive purposes and for comparisons of their dorsal parts with the other *Crocodylus* specimens but were excluded from the statistical analyses.

### 2.7 Data analyses

All subsequent analyses were performed in R 4.0.5 (R Core Team, 2021) using the package geomorph 4.0.0 for geometric morphometric analysis (Adams et al., 2016, 2022; Adams & Otárola‐Castillo, 2013). Generalised least‐squares Procrustes Analysis (GPA) superimposition was performed on the landmark dataset with the function *gpagen* to correct for differences in size and alignment of specimens.

Principal component analysis (PCA) was used on the Procrustes shape coordinates with the function *gm.prcomp* to find the major axes of shape variation within our sample. 3D wireframes were produced with *plotRefToTarget* to visualise shape modifications along PC axes with respect to the mean shape, obtained with *mshape* and *shape.predictor* functions. Data visualisation was performed with the package ggplot2 3.3.3 (Wickham, 2011).

First, we ran a PCA on the total dataset to visualise the variation of sinus shape during growth in all our modern Crocodylia samples. To assess the global effect of size, phylogenetic groupings, skull shape and lifestyle on sinus shape, we performed Procrustes ANOVA on the Procrustes coordinates in the total ontogeny morphospace using the function *procD.lm*, which corresponds to a non‐parametric Permutational Multivariate Analysis of Variance (Anderson, 2001). To accurately compare ontogenetic trajectories between species, we used Procrustes ANOVA of total shape and PC scores against log transformed centroid size and species (as a factor). A homogeneity of slopes test was performed using the *anova* function on the unique allometry (size × species) and common allometry (size + species) models to assess if species‐specific slopes were more appropriate than parallel allometric trajectories. The *pairwise* function was used to make pairwise comparisons of the means and variances of groups and ontogenetic trajectories. Finally, we used the *lm* function to trace regression lines between PC axes and log centroid size, and to visualise the axes of shape variation most correlated with size, using *ggplot* to assess ontogenetic changes in sinus shape.

Additionally, we ran three PCAs within each family (Alligatoridae, Crocodylidae and Gavialidae) to test if the principal axes of variance changed from one subclade to another, and if they could enable a better differentiation between species than in the total Crocodylia dataset (each clade‐specific analysis being independent from the total morphospace deformation induced by the differences of variance observed in each family). Statistical tests and linear regression on log‐transformed centroid size were performed both in the total dataset and in each subclade.

Both sides of the intertympanic sinus system were landmarked for visualisation purposes. To check how much variation was influenced by the asymmetry in our data, we used the function *bilat.symmetry* to quantify the symmetric and asymmetric component of shape variation and compare their statistical significance. The Procrustes ANOVA of the results found that 95.7% of the variation was significantly explained by the differences between individuals, while 0.2% was due to the directional asymmetry between both sides of the 3D models, and 4.1% coming from the fluctuating symmetry between individuals (interaction term). Thus, we considered that the impact of asymmetry on our results was marginal. Additionally, when performing PCA with only the medial and left side landmarks, the results are similar and do not change the interpretations.

### 2.8 Ancestral state reconstruction and phylogenetic mapping

A calibrated molecular phylogenetic tree was produced with Timetree 5 (Kumar et al., 2022) containing only the species for which we had at least three specimens to reconstruct an ontogenetic trend: *Alligator mississippiensis*, *Caiman crocodilus*, *Caiman latirostris*, *Tomistoma schlegelii*, *Gavialis gangeticus*, *Osteolaemus tetraspis*, *Mecistops cataphractus*, *Crocodylus siamensis/porosus* (considered as one) and *Crocodylus niloticus*. This topology was imported into R in Newick format using ape package 5.4‐1.

Reconstructions of the ancestral ontogenetic trajectories were undertaken with phytools package 1.2‐0. Coefficients of species‐specific linear models of PC1 and PC2 versus log‐centroid size were used to reconstruct ancestral states with *anc.ML* using maximum‐likelihood estimation. The mapping of these parameters on the phylogenetic tree was done with *contMap* to visualise the evolution of the intercepts and slopes of the ontogenetic trends along the tree branches. As species were represented by a small number of specimens (between three and 10), most 95% confidence intervals of the ancestral trajectories overlapped. Thus, we could hypothesise heterochronic shifts that occurred, following Alberch et al., 1979, based on the changes in rate and intercepts of the ontogenetic trajectory of a species compared to its ancestors, but these results have to be interpreted carefully. As such, a decrease or increase in slope corresponds to a deceleration or acceleration, implying a slowing or speeding in the rate of shape change. A decrease or increase in intercept corresponds to a post‐ or pre‐displacement, implying a change in the onset of development of the structure (occurring sooner or later in development respectively).

Finally, we used a consensus of the molecular topologies of Crocodylia from Pan et al. (2021), and Hekkala et al. (2021) to manually map adult intertympanic sinus characteristics and ontogenetic changes on phylogenetic relationships (Figure 13). Additionally, we made a second mapping on the morphological topology adapted from Rio and Mannion (Rio & Mannion, 2021) provided in Figure S16.

### 2.9 Institutional abbreviations

AMU, Aix‐Marseille Université, Marseille, France; CCEC, Centre de Conservation et d'Etude des Collections (Musée des Confluences), Lyon, France; FMNH, Field Museum of Natural History, Chicago, USA; INSA, Institut National des Sciences Appliquées, Lyon, France; ISEM, Institut des Sciences de l'Evolution de Montpellier, Montpellier, France; KIT IP, Karlsruhe Institute of Technology, Institute for Photon Science and Synchrotron Radiation, Karlsruhe, Germany; MHNL: Musée d'Histoire Naturelle de Lyon, Lyon, France; MHNM: Musée d'Histoire Naturelle de Marseille, Marseille, France; MNHN: Museum National d'Histoire Naturelle, Paris, France; MZS: Musée Zoologique de Strasbourg, Strasbourg, France; NHMUK: Natural History Museum, London, United Kingdom; OUVC, Ohio University Vertebrate Collection, Athens, USA; SMNK, Staatliches Museum für Naturkunde Karlsruhe, Karlsruhe, Germany; SVSTUA: Collections pédagogiques du Département de Biologie de l'Ecole Normale Supérieure de Lyon, Lyon, France; TMM, Texas Memorial Museum, Austin, USA; UCBL, Université Claude Bernard Lyon 1, Lyon, France; UF, University of Florida, Florida Museum of National History, Gainesville, USA; UM, Université de Montpellier, Montpellier, France; UMMZ, University of Michigan Museum of Zoology, Ann Arbor, USA; YPM, Yale Peabody Museum, New Haven, USA.

## 3 RESULTS

### 3.1 Comparative description of the intertympanic sinus system

A diverticulum is a soft‐tissue pocket of epithelial origin filled with air, forming a pneumatic sinus. Its development hollows out a ‘recess’, a cavity in the bone, generated by the growth of the pneumatic diverticulum (Kuzmin et al., 2021). In order to help appreciate the morphological variability occurring in our morphometric dataset, a comparative description of the intertympanic sinus system is provided for all modern crocodylian genera except *Paleosuchus*. Tw specimen of *Osteolaemus tetraspis* (MZS Cro 040) is used as a reference model for the description (Figures 1 and 2) because it displays well developed and differentiated diverticula compared to other species. Here, the term ‘intertympanic sinus system’ refers to the association of three pneumatic diverticula: the intertympanic diverticulum, parietal diverticulum and otoccipital diverticulum, which are more closely connected to each other than with the pharyngotympanic sinus system (Dufeau & Witmer, 2015), making it possible to study them as a separate unit.

**FIGURE 3:**
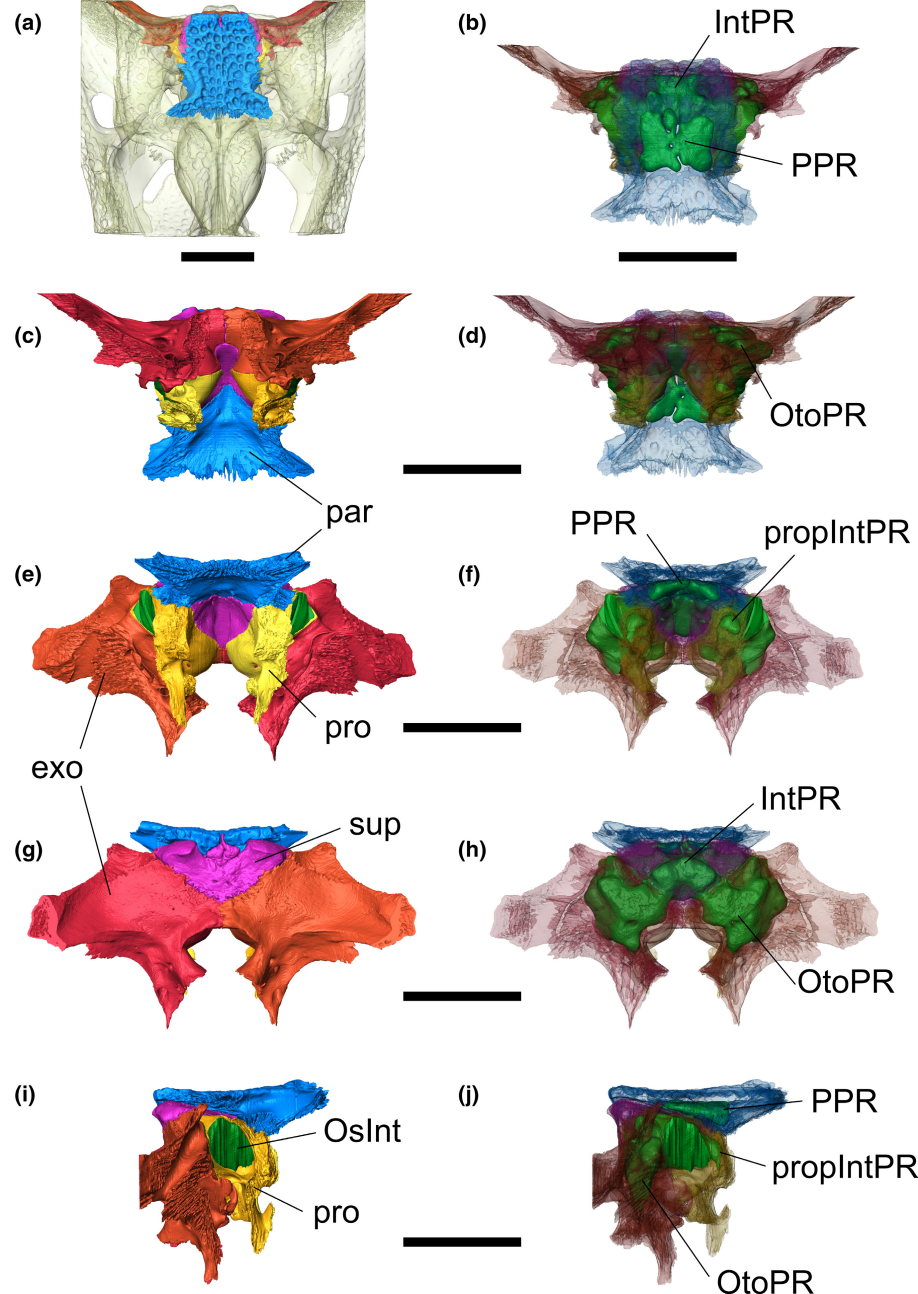
Volume renderings of the bone complex containing the intertympanic sinus system in the skull of *Osteolaemus tetraspis* (MZS Cro 040). (a, c, e, g, i) opaque and (b, d, f, h, j) semi‐transparent bones. (a, b) dorsal; (c, d) ventral; (e, f) anterior; (g, h) posterior; (i, j) right lateral views. Parietal in blue, supraoccipital in purple, paired prootic in yellow and gold, paired exoccipital in red and orange, intertympanic sinus system in green. exo, exoccipital; IntPR, intertympanic pneumatic recess; OsInt, ostium between intertympanic pneumatic recess and middle ear; OtoPR, otoccipital pneumatic recess; par, parietal; PPR, parietal pneumatic recess; pro, prootic; PropIntPR, prootic part of intertympanic pneumatic recess; sup, supraoccipital. Scale bars = 3 cm.

**FIGURE 4:**
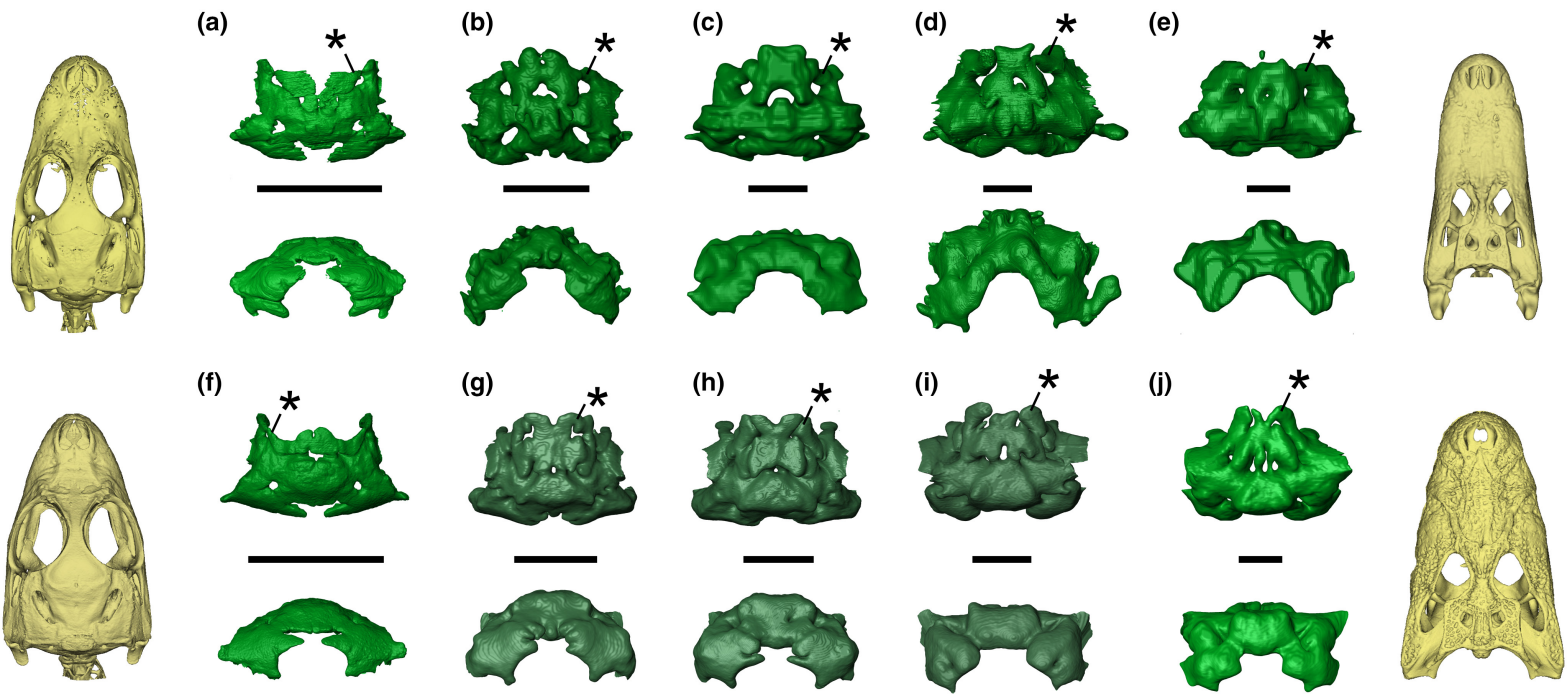
Ontogenetic variation in the intertympanic sinus system of *Alligator mississippiensis* and *Caiman latirostris* in dorsal (top) and posterior (bottom) views. (a‐e) *Alligator mississippiensis*; f–j, *Caiman latirostris* (a, SMNK REP 311; b, SMNK REP 311; c, TMM M 983; d, UCBL WB35; e, OUVC 9761; f, SMNK REP 314; g, UMMZ herp 155284; h, UMMZ herp 155285; i, UMMZ herp 155286; j, UMMZ herp 155287). (a, f) Hatchlings; (b, g) juveniles; (c, d, h) sub‐adults; (e, i, j) adults. Stars indicate the additional prootic‐parietal process present in all Alligatoridae studied. Volume renderings of skulls correspond to scale‐standardised extreme sizes of the ontogenetic series. Scale bars = 1 cm.

**FIGURE 5:**
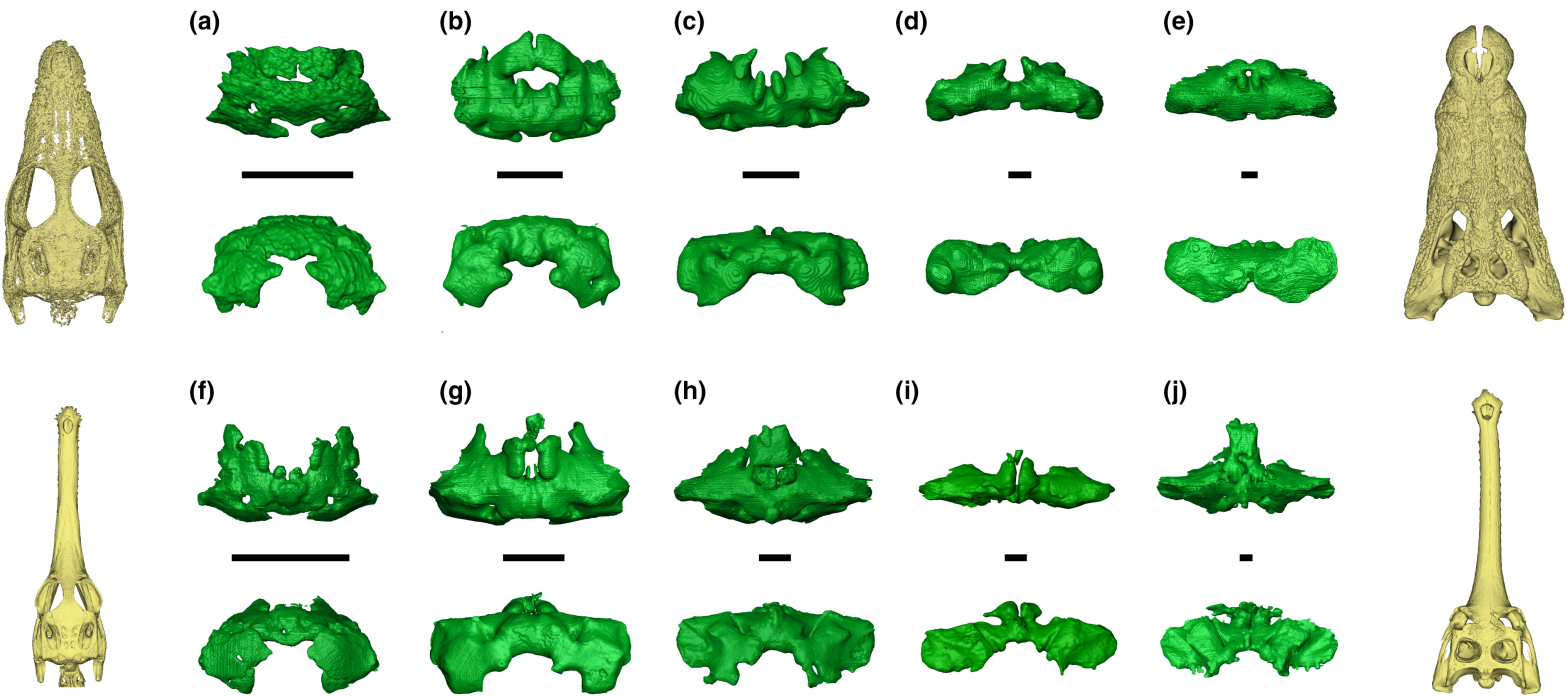
Ontogenetic variation in the intertympanic sinus system of *Crocodylus niloticus* and *Gavialis gangeticus* in dorsal (top) and posterior (bottom) views. a–e, *Crocodylus niloticus*; f–j, *Gavialis gangeticus* (a, SVSTUA 022002; b, MHNL 90001851; c, UM 2001–1756‐1‐434‐NR; d, MHNL 50001387; e, MHNL 50001405; f, YPM herr 008438; g, NHMUK 1846.1.7.3; h, NHMUK 1873; i, UF herp 118998; j, MHNL 50001407). (a, f) Hatchlings; (b) juvenile; (c, g) sub‐adults; (d, e, h, i, j) adults. Volume renderings of skulls correspond to scale‐standardised extreme sizes of the ontogenetic series. Scale bars equals 1 cm.

The intertympanic diverticulum occupies the dorsal part of the crocodylian braincase, carving a pneumatic recess through the paired prootics and the supraoccipital from one pharyngotympanic cavity to another, called the intertympanic pneumatic recess (IntPR, Figures 1 and 3). This recess is thinner in the initial stages of ontogeny and inflates dorsoventrally during growth (Figures 4 and 5). On each side, its communicating ostium with the pharyngotympanic cavity is formed by the perilymphatic loop of the prootic, situated dorsally to the lateral semi‐circular canal of the endosseous labyrinth and ventrolaterally to the internal suture between the prootic and the supraoccipital (Figure 3i). The dorsoventral thickness of the recess is greater in its lateral portion than in its medial portion (Figure 3f,h). The supraoccipital part of the intertympanic pneumatic recess communicates posteroventrally with the otoccipital pneumatic recess and anterodorsally with the parietal pneumatic recess (PPR; Figures 1d,e and 3j). The prootic part of the intertympanic pneumatic recess (propIntPR, Figure 3f,j) communicates anteroventrally with the prootic facial pneumatic recess (see Kuzmin et al., 2021, figures 11, 24) in all hatchlings and small juveniles studied. This communication is still present in adult specimens of *Alligator*, *Caiman* and *Osteolaemus*.

The otoccipital diverticulum creates a recess inside the exoccipital (called the otoccipital pneumatic recess, OtoPR; Figures 1 and 3d,h,j), which communicates with the intertympanic pneumatic recess anterodorsally by two pairs of openings located in the internal suture between the exoccipital and the supraoccipital. It is linked ventrally with the rhomboidal recess (the ventral part of the pharyngotympanic sinus; see Dufeau & Witmer, 2015; Kuzmin et al., 2021), and anteroventrally with the posteroventral part of the pharyngotympanic cavity. The otoccipital pneumatic recess develops from the posterior part of the pharyngotympanic sinus and connects with the intertympanic sinus during early ontogeny (Kuzmin et al., 2021). It is well developed in juvenile crocodylians of all genera, displaying a rounded diamond‐shape in posterior view, and is still strongly connected ventrally with the pharyngotympanic cavity (Figures 4a,b,f,g and 5a,b,f,g). During ontogeny, the size of the supraoccipital‐exoccipital foramina increases while the communications with the pharyngotympanic sinus diminish, and the otoccipital pneumatic recess coalesces with the intertympanic pneumatic recess to the point when it becomes difficult to discriminate the two recesses in large individuals (Figures 4e and 5c–e,h–j).

The parietal diverticulum hollows the ventral surface of the parietal out, forming the PPR (Figures 1 and 3b,f,h,j). It originates from the intertympanic diverticulum through several foramina in the supraoccipital‐parietal suture. The number and development of these processes invading the parietal differ depending on the taxon. Two pairs of openings are typically present: a larger anterolateral pair and a smaller posteromedial pair, called the anterolateral and posteromedial pre‐parietal processes, respectively (app & ppp, Figure 1), the latter being either reduced or fused in some taxa (Figures S1–S10). Additionally, a third pair of foramina links the anterolateral part of the PPR with the prootic part of the intertympanic pneumatic recess through the prootic‐parietal internal suture in all studied Alligatoridae. The resulting structure is called the prootic‐parietal process (Figure 4, Figures S1–S3). One specimen of our dataset (*Osteolaemus* UCBL 2019‐1‐236) lacks the development of a parietal diverticulum.

### 3.2 Alligator mississippiensis

*Alligator mississippiensis* displays an anteroposteriorly and dorsoventrally large intertympanic pneumatic recess; its prootic part connects with the prootic facial recess in juveniles and adults alike (Figure 4a–e). The intertympanic diverticulum inflates during growth, as the bony walls separating the parts of the intertympanic system are obliterated during ontogeny. The otoccipital pneumatic recess is well developed and remains voluminous and well distinct from the intertympanic pneumatic recess in adults, despite large communicating ostia and a slight lateral compression (Figure 4d,e). The posteromedial pre‐parietal processes are located posteriorly and fused in a single small cavity divided into two bulges. They are generally fused anteriorly with the anterolateral pre‐parietal processes, the latter being larger and merged in the medial portion of the parietal. The resultant parietal diverticulum is thick and expands anterodorsally during ontogeny. The anterolateral portion of the parietal bears a pair of prootic‐parietal processes that connects with the prootic part of the intertympanic diverticulum through a large ostium (Figure 4). The three diverticula of the system are greatly fused in the adult specimen (Figure 4e).

### 3.3 Caiman

*Caiman* possesses a similar intertympanic sinus system as *A. mississippiensis*. Its large intertympanic pneumatic recess displays a prootic part connected both with the prootic facial recess ventrally and with the anterolateral part of the PPR dorsally through the prootic‐parietal process. The otoccipital pneumatic recess is well‐developed and remains voluminous and distinct in all three species (Figures S2 and S3), with large communicating ostia with the intertympanic pneumatic recess. It is slightly stretched anterodorsally and compressed laterally as size increases, showing a verticalised crescent shape in posterior view in adult specimens (Figure 4h–j, Figures S2 and S3). Like in *A. mississippiensis*, the parietal diverticulum is linked to the intertympanic diverticulum by three pairs of processes, which are generally completely fused inside the parietal and form a single cavity even in younger stages (Figure 4f–j). The PPR is expanded earlier in ontogeny, and it becomes thick in later stages, while acquiring a flattened roof parallel to the skull table. In *Caiman*, the anterolateral and posteromedial pre‐parietal processes are located roughly at the same level anteroposteriorly (the larger ‘anterolateral’ processes are therefore positioned laterally, and the smaller ‘posteromedial’ processes are positioned medially). The posteromedial pre‐parietal processes are often reduced or obliterated in adult stages. Adult *C. crocodilus* and *C. yacare* display a similar shape, with a lesser anteroposterior expansion and greater verticalisation compared to *C. latirostris* (Figures S2 and S3). The latter presents a rounded sinus system and greatly expanded diverticula with a greatly developed PPR throughout all life stages (Figure 4f–j).

### 3.4 Melanosuchus

Adult *M. niger* present an intertympanic sinus system less expanded than in adult *A. mississippiensis* and *Caiman*. Like other Alligatoridae genera, the prootic part of the intertympanic pneumatic recess is connected to the prootic facial recess ventrally and to the PPR dorsomedially (Figure S3). The intertympanic recess is more compressed anteroposteriorly and the bony walls separating it from the otoccipital recess are thick, making the two recesses well differentiated. The otoccipital pneumatic recess displays a tubular aspect, protruding posteriorly, but showing a verticalised crescent shape in posterior view like adult specimens of *Caiman* (Figure S3). The posteromedial pair of pre‐parietal processes is absent while the anterolateral pair is expanded, creating large cylindric cavities that merge anteriorly with the prootic‐parietal process, forming a tubular PPR.

### 3.5 Crocodylus

The intertympanic sinus system of *Crocodylus* is anteroposteriorly short and laterally elongated in adults. In juveniles, it is rounded and bulky; it undergoes an anteroposterior and dorsoventral compression as size increases, which is more prominent in the medial part of the intertympanic diverticulum (Figure 5a–e). In this genus, the morphology of the intertympanic sinus system differs between species, with respect to the dorsoventral thickness of the intertympanic diverticulum, the development of the parietal diverticulum and the reduction of the otoccipital diverticulum at adult stages (Figures S4 and S5).

In *C. niloticus*, hatchling and juvenile specimens possess a developed parietal diverticulum formed by the two anterolateral pre‐parietal processes merged medially (Figure 5a,b). In adults, the pre‐parietal processes entering the PPR project anteromedially and are not well developed, as they rarely form a single cavity inside the parietal, except in large individuals where the tips of these processes merge (Figure 5c–e). During ontogeny, the otoccipital diverticulum is greatly reduced ventrally and merges into the intertympanic diverticulum as the bony walls containing the supraoccipital‐exoccipital ostia are obliterated. The adult specimen of *C. halli* shares a similar morphology as *C. niloticus* (Figures S4 and S5).

*Crocodylus siamensis* and *C. porosus* share a common morphology easily distinguishable from other genera. The juvenile specimen of *C. porosus* does not show a developed PPR, with only the posteromedial pre‐parietal process visible. However, adult individuals possess a more expanded and verticalised system than the other species of the genus. The intertympanic recess is thicker. Their otoccipital pneumatic recess is slightly reduced laterally but is developed ventrally and well differentiated from the intertympanic pneumatic recess, forming a butterfly shape in posterior view (Figure S5). The pre‐parietal processes forming the PPR are larger than in *C. niloticus*, and all four processes are greatly developed dorsally and anteriorly, contacting in the anterior part of the parietal to form a square‐shaped recess in frontal view.

The adult specimen of *C. palustris* shares similarities with *C. siamensis* and *C. porosus*, with dorsoventrally developed diverticula and verticalised parietal and otoccipital recesses (Figure S5). The prootic part of the intertympanic recess is voluminous, and the otoccipital recess is flattened anteroposteriorly. The parietal recess is expanded dorsally but is restricted anteriorly, not exceeding the anterior limit of the prootic part of the intertympanic recess, contrary to *C. siamensis* and *C. porosus*.

American *Crocodylus* species also possess different intertympanic morphologies. *Crocodylus acutus* possesses an intertympanic sinus system with an intermediate shape between the *C. niloticus* and *C. siamensis* patterns: the otoccipital pneumatic recess is still developed ventrally, and the PPR displays larger anterolateral processes that do not merge with each other (Figure S5). *Crocodylus rhombifer* displays an intertympanic diverticulum more similar to *C. niloticus*: the PPR has its posteromedial parietal processes oriented dorsally, and is small or nearly absent, whereas the anterolateral parietal processes are larger and merged in the medial part of the parietal (Figure S5). However, our available *C. rhombifer* specimens are internally damaged, which prevented a proper assessment of the ventral part of the sinus system.

### 3.6 Osteolaemus

*Osteolaemus tetraspis* presents a voluminous sinus system throughout all stages studied, with few differences between juveniles and adults. The intertympanic pneumatic recess is anteroposteriorly and dorsoventrally large. Its prootic part protrudes anteriorly and is generally connected with the prootic facial recess ventrally (see also Kuzmin et al., 2021). The otoccipital pneumatic recess is highly developed and connected to the intertympanic pneumatic recess through large ostia, remaining well separated from the latter when size increases. It maintains its relative volume throughout ontogeny, despite a slight compression of its ventral part. The anterolateral pair of pre‐parietal processes is large and expanded in voluminous paired parietal cavities developed anteroposteriorly and laterally. In adult stages, these paired cavities merge into a single PPR. The posteromedial pre‐parietal processes form two bulges oriented vertically and are anteriorly connected to the parietal recess (Figures 1 and 3 and Figure S6).

### 3.7 Mecistops

*Mecistops* possesses an anteroposteriorly short intertympanic sinus system which resembles that of sub‐adult *C. niloticus* (Figure S7). The intertympanic pneumatic recess is compressed anteroposteriorly as size increases. The otoccipital pneumatic recess is reduced laterally but still developed ventrally in adults and undergoes an increased anteroposterior compression and a reduction of the ventral part in large specimens. Only the anterolateral pair of pre‐parietal processes forms the PPR, which is connected through large ostia to the intertympanic recess. These processes merge in the anterior part of the parietal in adults, creating a tubular PPR, which is oval‐shaped in dorsal view. The anterior part of the PPR is compressed anteriorly in adults (Figure S7). The posteromedial pair of pre‐parietal processes is absent in sub‐adult individuals and absent or reduced in adults: it does not cross the supraoccipital‐otoccipital suture and does not participate in the parietal cavity.

### 3.8 Gavialis

**FIGURE 6:**
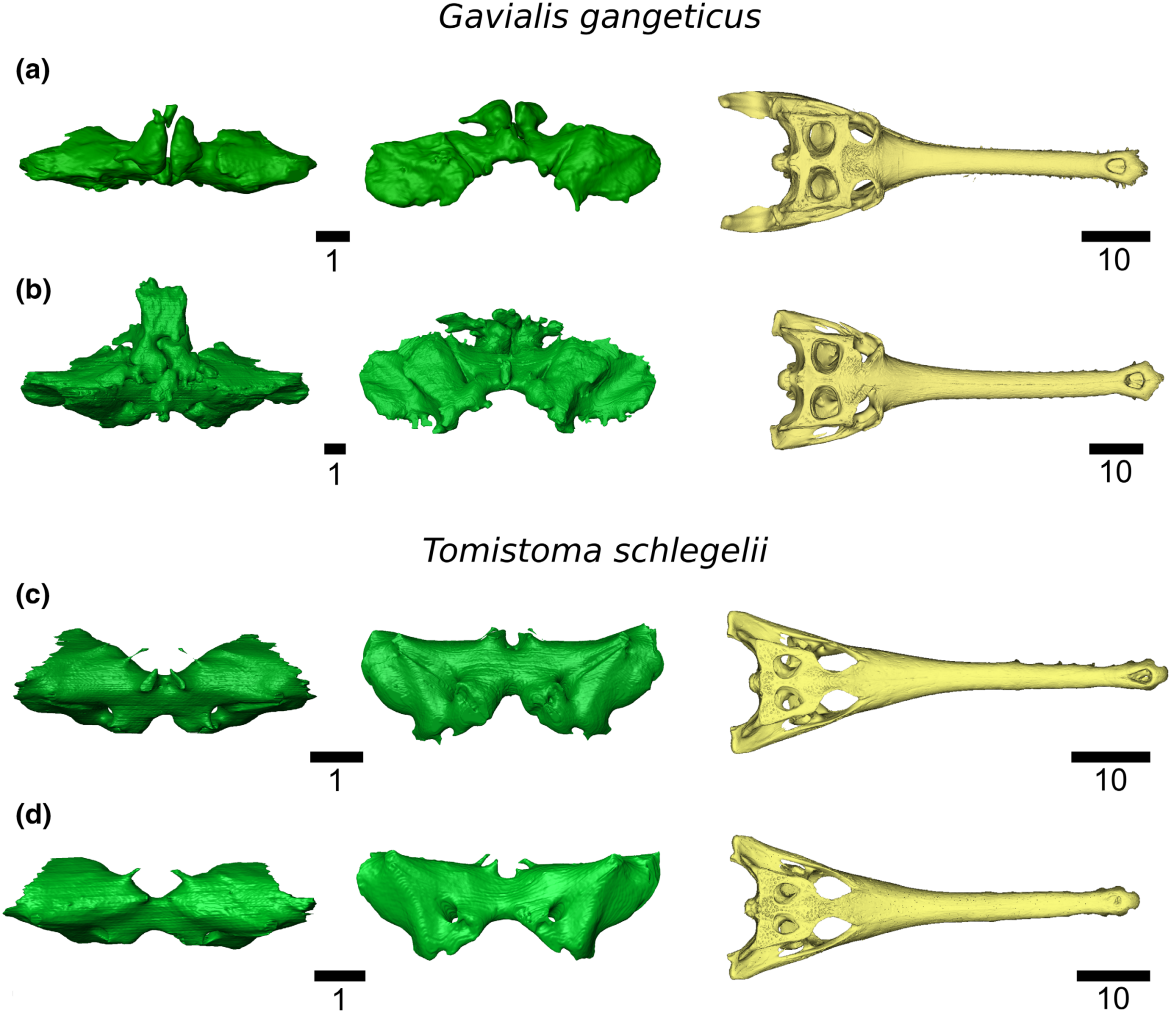
Morphological differentiation of the intertympanic sinus system between adult *Gavialis* and *Tomistoma*. From left to right: dorsal and posterior views of the intertympanic sinus; dorsal view of the 3D models of the corresponding skulls. (a) UF herp 118998; (b) MHNL 50001407; (c) MZS Cro 094; (d) NHMUK 1893.3.6.14. Scale values are given in cm.

*Gavialis gangeticus* displays a compressed intertympanic sinus system in adult stages (Figures 5i,j and 6a,b). The juvenile specimen shows the same pattern as other species at the same size, with a flat and rounded intertympanic sinus system. At this stage the two pairs of pre‐parietal processes are present but not well developed (Figure 5f). In the sub‐adult specimen, the otoccipital pneumatic recess is compressed anteroposteriorly and slightly reduced ventrally. The anterolateral pair of pre‐parietal processes is inflated, anteroposteriorly large and slightly verticalised, whereas the posteromedial pair is extremely reduced (Figure 5g). In adults, the intertympanic sinus system is highly modified: the intertympanic pneumatic recess is elongated laterally and compressed anteroposteriorly and dorsoventrally, and it is narrower in its medial part. Its lateral parts are slightly oriented ventrally, showing a convex roof in transverse view (Figures 5h–j and 6a,b, Figure S8). The otoccipital pneumatic recess is nearly completely merged with the intertympanic pneumatic recess, with little ventral expansion. The PPR is only formed by the anterolateral pre‐parietal processes. They form paired bulges projected dorsally, which then extend anteriorly, coalescing when size increases (Figure 5h–j). Large specimens show a voluminous PPR which is elongated anteroposteriorly, while the posteromedial pre‐parietal processes are completely absent (Figure 5j).

### 3.9 Tomistoma

*Tomistoma* possesses an anteroposteriorly compressed intertympanic sinus system which does not bear any developed PPR (Figure 6c,d, Figure S9). The intertympanic pneumatic recess is elongated laterally during growth and undergoes a prominent compression of its medial part when size increases, both in the anteroposterior and dorsoventral directions. The lateral parts of the intertympanic sinus are slightly oriented dorsally, showing a concave roof in transverse view (Figure 6c,d). Sub‐adult specimens still display a developed otoccipital recess, which is rapidly reduced ventrally and laterally during growth (Figure S9). This recess is thinner in adults but still distinguishable from the intertympanic pneumatic recess. The PPR is completely absent in all stages studied. In juvenile and sub‐adult specimens, the pre‐parietal processes are reduced. During ontogeny, the anteroposterior compression of the system is accompanied with a nearly complete reduction of the anterolateral pre‐parietal processes, which become thin and end at the supraoccipital‐parietal suture. The posteromedial pre‐parietal processes are present and oriented vertically but without entering the parietal (Figure 6c,d).

### 3.10 Voay

The intertympanic sinus system of *V. robustus* displays an expanded pneumatisation (Figure 11a and Figure S10). The intertympanic pneumatic recess is anteroposteriorly and dorsoventrally large in adults. Its prootic part is voluminous and developed anteriorly, and it is connected anteroventrally to the prootic facial recess like in *Osteolaemus*. The otoccipital pneumatic recess is voluminous with almost no lateral compression: it is well developed ventrally, and it is connected through large ostia to the rhomboidal sinus (Figure 11a). The anterolateral pre‐parietal processes are directed anteriorly and merged in a single bulbous cavity in two out of the three specimens studied. The posteromedial pre‐parietal processes are directed vertically and can be connected anteriorly to the anterolateral processes. The development of the PPR seems to differ in each individual studied (Figure S10). In MNHN‐F‐1908‐5 it is greatly inflated, and merges with a great pneumatic cavity oriented anteriorly that reaches the frontal. In the NHMUK specimens it is less expanded, and the anterolateral pre‐parietal processes is also developed in the posterior direction in NHMUK‐PV‐R‐36685, like some specimens of *Osteolaemus* (Figure S6).

## 4 QUANTIFICATION OF INTERTYMPANIC SINUS SHAPE

Procrustes ANOVA revealed a significant effect of both size and taxonomic grouping on sinus shape (*p* = 0.001), with size accounting for 21.1% of the overall variation, while 42.4% of the variability is linked to the species‐specific intertympanic sinus morphology. The snout shape of the specimens (brevirostrine, mesorostrine or longirostrine, see Table 1) also accounts for 14.5% of the variation (Table S2).

**FIGURE 7:**
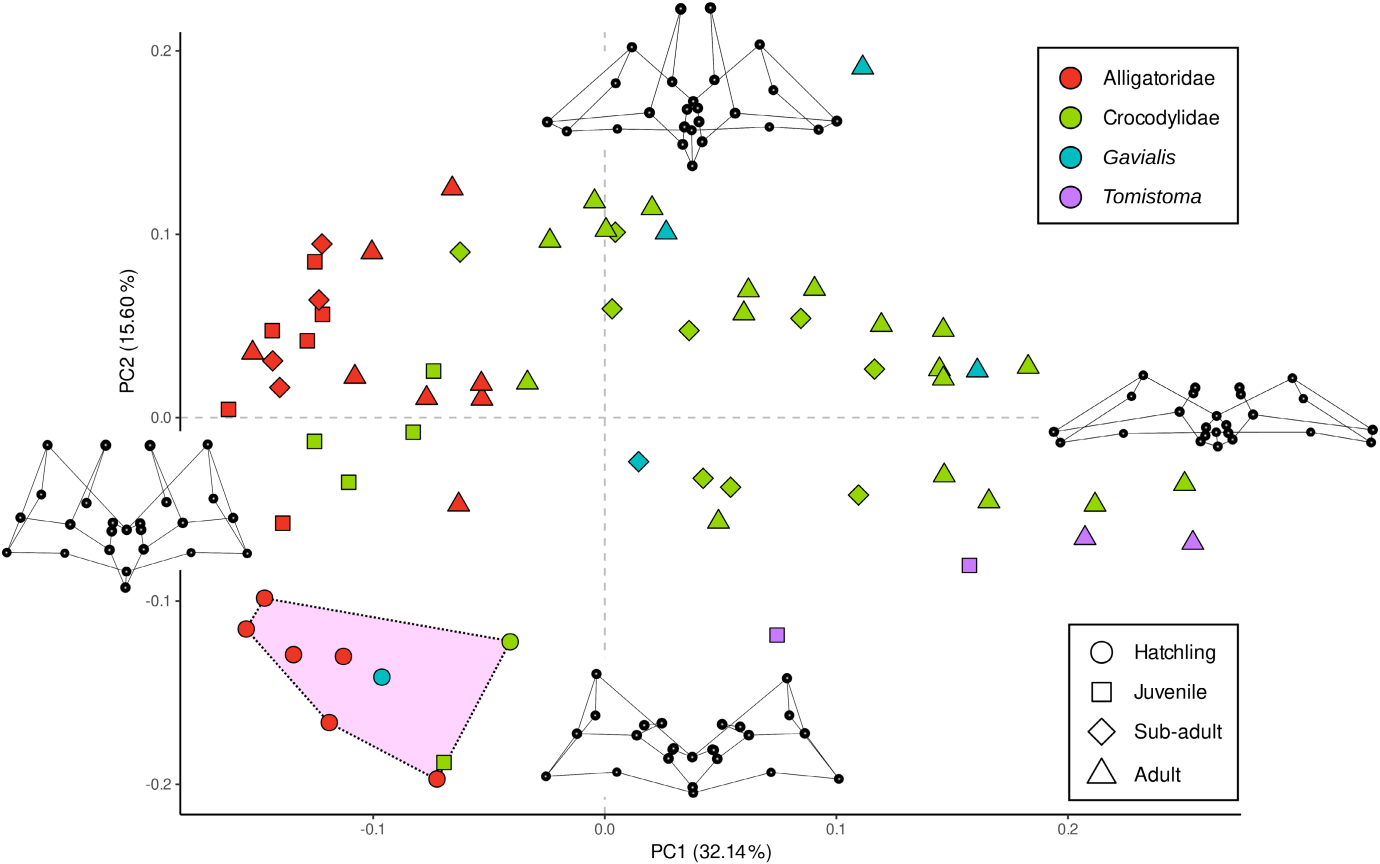
Ontogenetic morphospace of intertympanic sinus system shape showing differences between the distributions of Alligatoridae (red), Crocodylidae (green), *Tomistoma* (purple) and *Gavialis* (blue) for the four size classes in the first two principal components. Extreme shapes for each PC axis are given with wireframes in dorsal view. The zone shaded in pink is the region of the morphospace containing all studied hatchlings (CHR, ‘common hatchling region’).

The first two principal components of the complete ontogenetic morphospace explain 47.7% of the total variance, showing important morphological changes in intertympanic sinus system shape throughout ontogeny (Figure 7). The remaining components each accounted for less than 10% of the total variance. PC1 shows changes in the anteroposterior compression and lateral expansion of the intertympanic sinus system, with positive values corresponding to an anteroposteriorly compressed sinus system. PC2 characterises the development of the pre‐parietal processes, with positive values associated with an anteroposteriorly and vertically expanded parietal diverticulum. PC scores along these two principal components are both correlated with size and species groupings (score values delimiting species morphospaces). Size explains 45.9% and 39.4% of the variation, respectively (*p* = 0.001, see Table S3), with larger specimens located towards the positive ends of PC1 and PC2.

Hatchlings of all species studied are congregated in a single region of the morphospace towards the negative ends of PC1 and PC2 (pink shaded area on Figure 7), which we called the ‘common hatchling region’ (CHR). They all display an anteroposteriorly and dorsoventrally large, laterally short and rounded intertympanic sinus system with small or absent pre‐parietal processes. Generally, as size increases, the parietal recess develops, with larger specimens gathering towards PC2‐positive values, while the intertympanic and otoccipital recesses undergo a variable anteroposterior compression, with larger specimens gathering towards PC1‐positive values. During ontogeny, each family explores distinct parts of the morphospace.

Alligatoridae are confined to PC1‐negative values with little dispersion along this axis, showing only a slight anteroposterior compression in adult specimens (Figure 7). On the other hand, their parietal recess is well developed and inflates during growth, with most post‐hatching *Caiman* and all post‐hatching *A. mississippiensis* specimens dispersed along PC2‐positive values (Figure S12).

Crocodylidae are the most morphologically dispersed specimens, being distributed along a parabola ranging from the CHR to the positive end of PC1. From the common hatchling morphology, juveniles first display a development of the parietal diverticulum (displacement towards the positive values of PC2). Sub‐adult and adult specimens are then distributed along PC1 as their sinus system undergoes anteroposterior compression, with larger individuals possessing the most positive PC1 values and a heavily compressed sinus system (Figure 7). Depending on the individual development of their parietal diverticulum, adult Crocodylidae are pushed towards more or less positive PC2 values. *Crocodylus porosus* and *C. siamensis* specimens occupy the most positive values associated with a well‐developed and verticalised parietal recess, while *C. niloticus* sits in the middle of the morphospace or displays weakly negative PC2 values due to a reduced parietal recess (Figure 7, Figure S13).

The hatchling specimen of *Gavialis* falls in the CHR (Figure 7). The sub‐adult is found around the mean shape (null values), characterised by a development of the parietal processes and a greater anteroposterior compression of the sinus system. Adult specimens are dispersed in a large array of PC1‐ and PC2‐positive values, depending on the individual development of their parietal and intertympanic recess, with the largest specimen of *Gavialis* (MHNL 50001407, SW = 25.5 cm) found in extreme PC2‐positive values due to its extremely elongated parietal recess (Figure 6b). On the other hand, *Tomistoma* specimens are distributed in a completely different region of the morphospace. Due to the reduction and near obliteration of their parietal processes and an increased intertympanic recess anteroposterior compression, they are found in PC2‐negative values, with larger specimens in higher PC1‐positive values (Figure 7).

## 5 ONTOGENETIC TRAJECTORIES AND ANCESTRAL TRENDS RECONSTRUCTION

Procrustes ANOVA revealed significant differences in allometric trajectories between species (interaction term size: species, *p* = 0.001, see Table S2). Comparisons of the allometry models showed that species‐specific allometric trends were significantly better than a common allometry model. However, due to the heterogeneity in ontogenetic sampling and the scarcity of specimens in some species, the pairwise comparisons of ontogenetic trajectories in the unique allometry model did not reveal any significant pairwise differences, whereas pairwise comparisons in the common allometry model revealed significant differences between most studied trajectories (Data S6 and S7).

Only species that showed substantial positive allometry, resolved ontogenetic series and a small variance display statistically significant ontogenetic trends (Figures S17, S18, Table S5). On PC1 (anteroposterior and dorsoventral compression of the intertympanic sinus), significant relationships were retrieved for *Crocodylus*, *Mecistops* and *Gavialis*, with *C. niloticus* and *Mecistops* showing the steepest slopes. Alligatorid trends are not significant. However, *A. mississippiensis* still presents a slightly negative allometric trajectory, which is consistent with our qualitative observations that demonstrate an inflation of the intertympanic sinus system during growth rather than a compression (Figure 4a–e, Figure S17). On PC2 (growth of the PPR), *A. mississippiensis*, both *Caiman* species, *C. porosus*, *C. siamensis* and *G. gangeticus* also show a significant relationship between their shape and size. They all possess an anteriorly and dorsally developed parietal recess, with alligatorids having the steepest slopes. Significant trends were not retrieved on PC2 in species for which the development of the pre‐parietal processes is minimal and/or variable depending on the specimen, like *C. niloticus*, *O. tetraspis* and *T. schlegelii* (Figure 5c–e and Figure S18). Additionally, *Mecistops* shows a negative trend on PC2, corresponding to a flattening of the parietal recess during growth (Figure S22b). Yet, as we were not able to study hatchlings and juveniles of this genus, the complete trajectory remains unknown. The ontogenetic trends obtained on PC1 and PC2 were used to estimate the ancestral trends on each node (Figure S19, Table S6).

**FIGURE 8:**
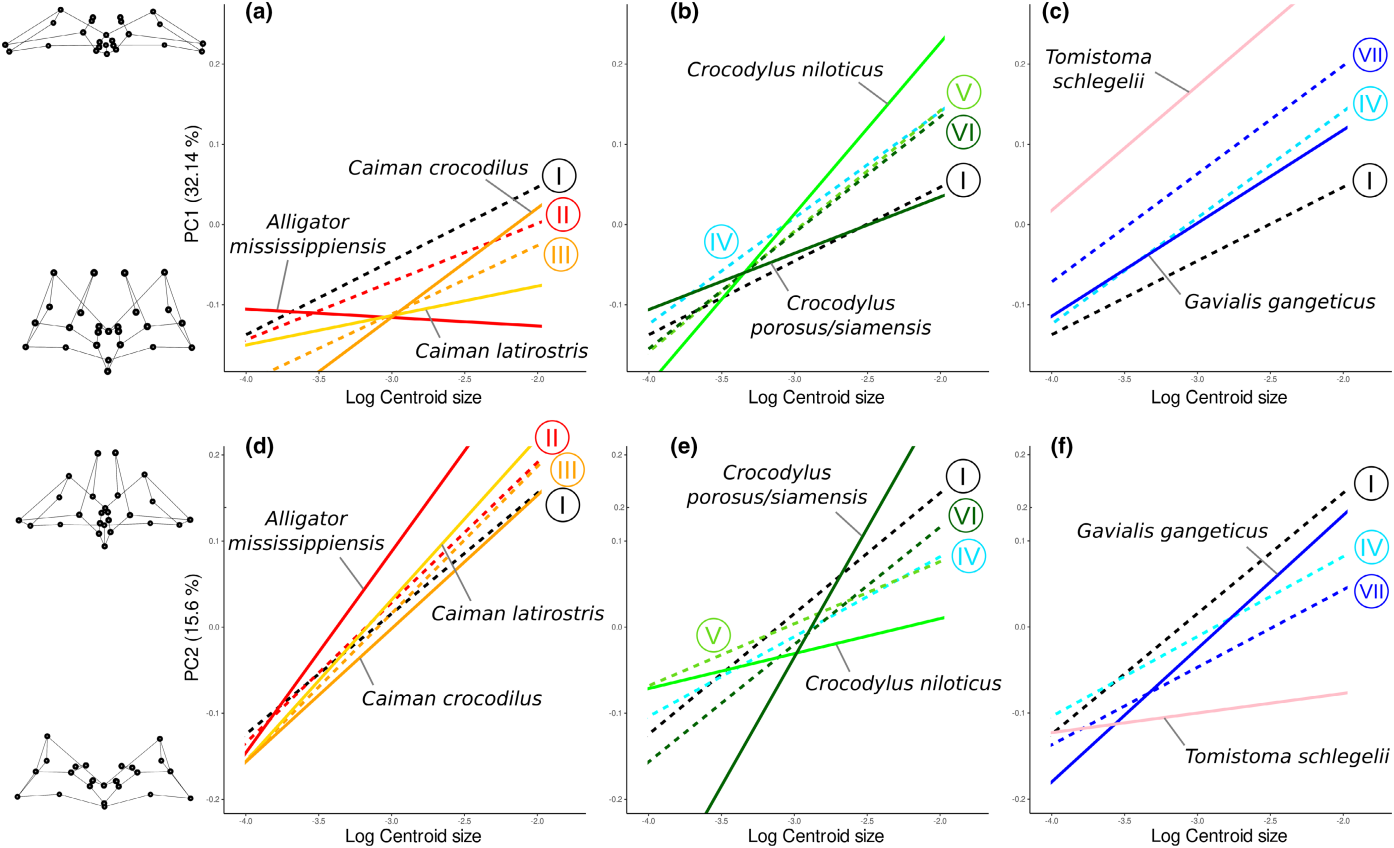
Reconstructed PC1 and PC2 ontogenetic trajectories of seven crocodylian species with estimated ontogenetic trends at ancestral nodes. Left: Alligatoridae; middle: Crocodylidae; right: Gavialidae. (a, b, c) PC1 ontogenies; (d, e, f) PC2 ontogenies. Dashed lines are hypothetical ancestral ontogenetic trajectories (roman numerals correspond to Figure 13). Extreme shapes for each PC axis are given with wireframes in dorsal view.

Hypothetical developmental patterns of ancestral nodes were obtained by maximum likelihood from previous trend parameters (Figures S20 and S21). As such, most of the confidence intervals overlap due to gaps in our ontogenetic series (Table S6). However, as the observed trajectories match the morphological observations, they can still provide some clues for interpreting the evolutionary mechanisms underlying sinus development. The estimated crocodylian ancestor displays a moderate compression of the system, close to the *Osteolaemus* ontogenetic trajectory (PC1 trajectories, Figure S22a), and a development of the parietal recess that falls within the range of the Caimaninae patterns (PC2 trajectories, Figure 8d).

On PC1, two opposite events separate the crown group from its ancestry: on the one hand, a deceleration of sinus compression in Alligatoridae; on the other hand, an acceleration of the compression in Longirostres (nodes II and IV, Figure 8a–c). In Alligatoridae, *A. mississippiensis* undergoes greater deceleration, showing even more inflated sinuses. Caimaninae are characterised by a post‐displacement of the onset of their PC1 trajectory (node III), with *C. latirostris* showing further deceleration, implying smaller changes between hatchling and adult shape in sinus anteroposterior expansion (Figure 8a). In Crocodylidae, it seems that the differences in PC1 allometric trajectories mainly result from variations in the speed of sinus development. *Crocodylus niloticus* and *Mecistops* show a prominent acceleration of the compression, whereas *C. porosus/siamensis* and *Osteolaemus* are characterised by a deceleration and retention of an expanded intertympanic recess in adults (Figure 8b, Figure S21a). In contrast, differences in the PC1 trajectories of gavialids result from variation in the onset of sinus development. Their ontogenetic trajectories are similar in slope but different in elevation. The *Gavialis* trajectory is quite close to the Longirostres ancestral trend, whereas *Tomistoma* undergoes a prominent pre‐displacement, characterised by an increased compression of its sinus system in all stages studied (Figure 8c).

On PC2, Alligatoridae and Longirostres also present opposite trends, with the former undergoing an acceleration of parietal recess development, and the latter showing a deceleration of its development. *Caiman latirostris* and *A. mississippiensis* both develop a parietal recess more rapidly than other species during growth (Figure 8d). The dichotomy between *Crocodylus* species is particularly visible in the allometric trajectory of their parietal recess: *C. niloticus* undergoes both pre‐displacement and deceleration, while *C. porosus/siamensis* undergoes a post‐displacement and a sharp acceleration (Figure 8e). Both gavialids display a post‐displacement of the onset of their parietal recess development. *Gavialis* underwent an acceleration, whereas *Tomistoma* shows a clear deceleration, with almost no development of the parietal recess (Figure 8f).

### 5.1 Sub‐family analyses

#### 5.1.1 Alligatoridae

**FIGURE 9:**
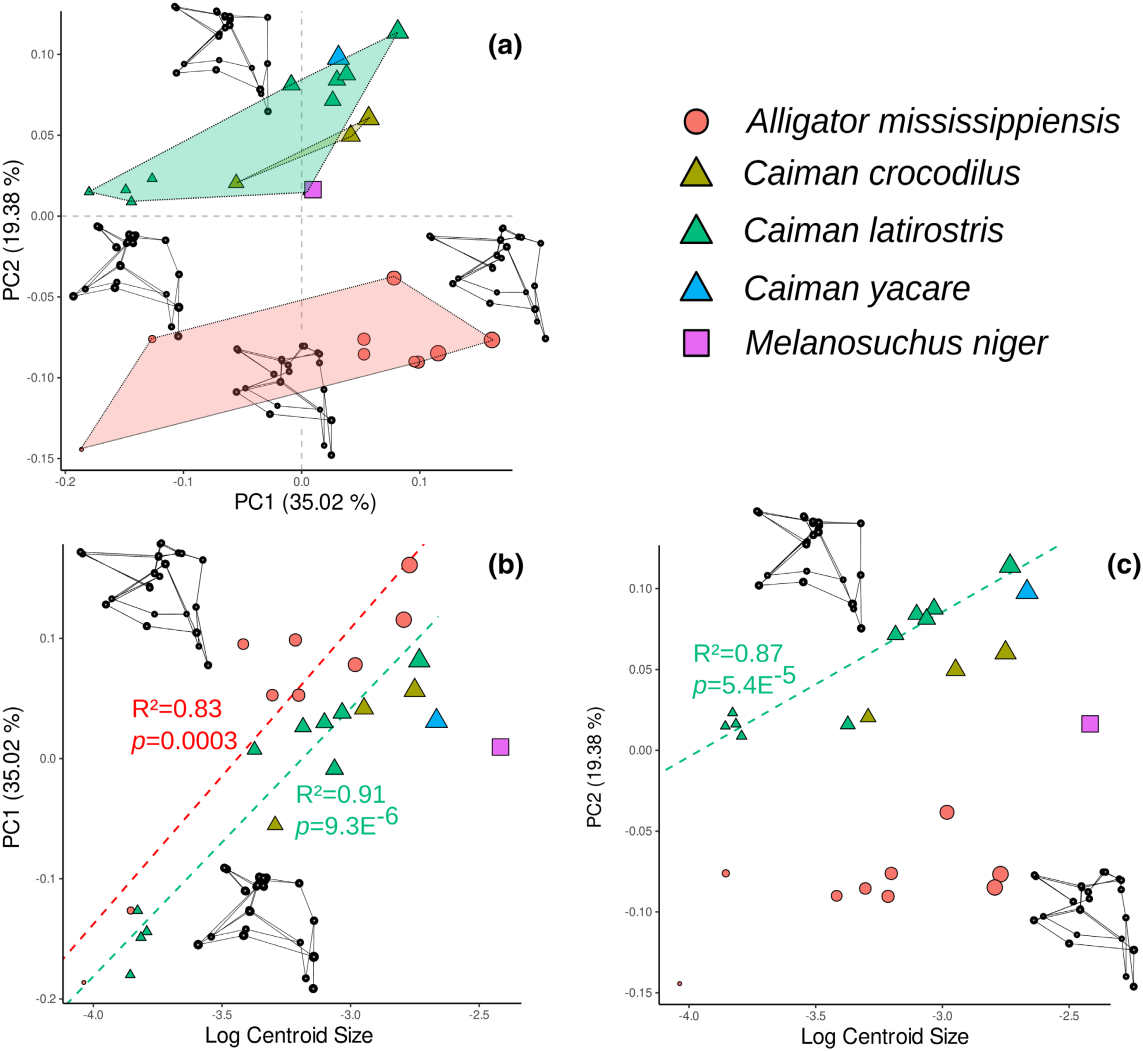
Alligatoridae ontogenetic morphospace of intertympanic sinus and relation to size. (a) PCA results; (b, c) plots of the principal components more correlated with size versus log‐transformed centroid size, associated with the significant ontogenetic trends (regression of the principal components scores vs. log centroid size for each species respectively). Extreme shapes for each PC axis are given with wireframes in left lateral view.

The Alligatoridae‐only PCA allows a clear discrimination between Caimaninae and *A. mississippiensis* in the morphospace created by the two first components, explaining 54.4% of their morphological variation (Figure 9a). PC1 scores are significantly correlated with size (*p* = 0.001, *R* ^2^ = 0.65, see Table S4), and to a lesser extent with taxonomic grouping (*p* = 0.001, *R* ^2^ = 0.25). Ontogenetic series are thus distributed along PC1 from negative to positive values and are associated with the anterior expansion of the parietal recess during growth (Figure 9b). The shape variation along PC2 highlights differences in Alligatoridae, as it is mainly explained by the species‐specific morphology (*p* = 0.001, *R* ^2^ = 0.82). It is correlated with the position of the posteromedial pre‐parietal processes that discriminates the two subfamilies: *A. mississippiensis* are confined to negative values of PC2 and characterised by processes that develop dorsally, while Caimaninae are found in positive values, showing processes that develop anteriorly (Figure 9c).

The allometric trajectories in this morphospace showed that the main changes that occur during alligatorid growth mainly involve the PPR. PC1 trajectories show that species undergo a continuous anteroposterior development of the parietal recess, which is strongly correlated with size (Figure 9b and Table S7). *Alligator mississippiensis* shows a displacement towards PC1‐positive values, implying a larger parietal recess compared to Caimaninae of similar size. Both genera clearly span different range of PC2 scores, so that *A. mississippiensis* and *Caiman* start their post‐hatching growth with marked differences in the shape and orientation of the posteromedial pre‐parietal processes. Then, both genera show a slight anterior displacement of these processes, with adult *Caiman* displaying anteriorly displaced posteromedial pre‐parietal processes, which clearly contrast with the morphology observed in adult *A. mississippiensis*.

#### 5.1.2 Crocodylidae

**FIGURE 10:**
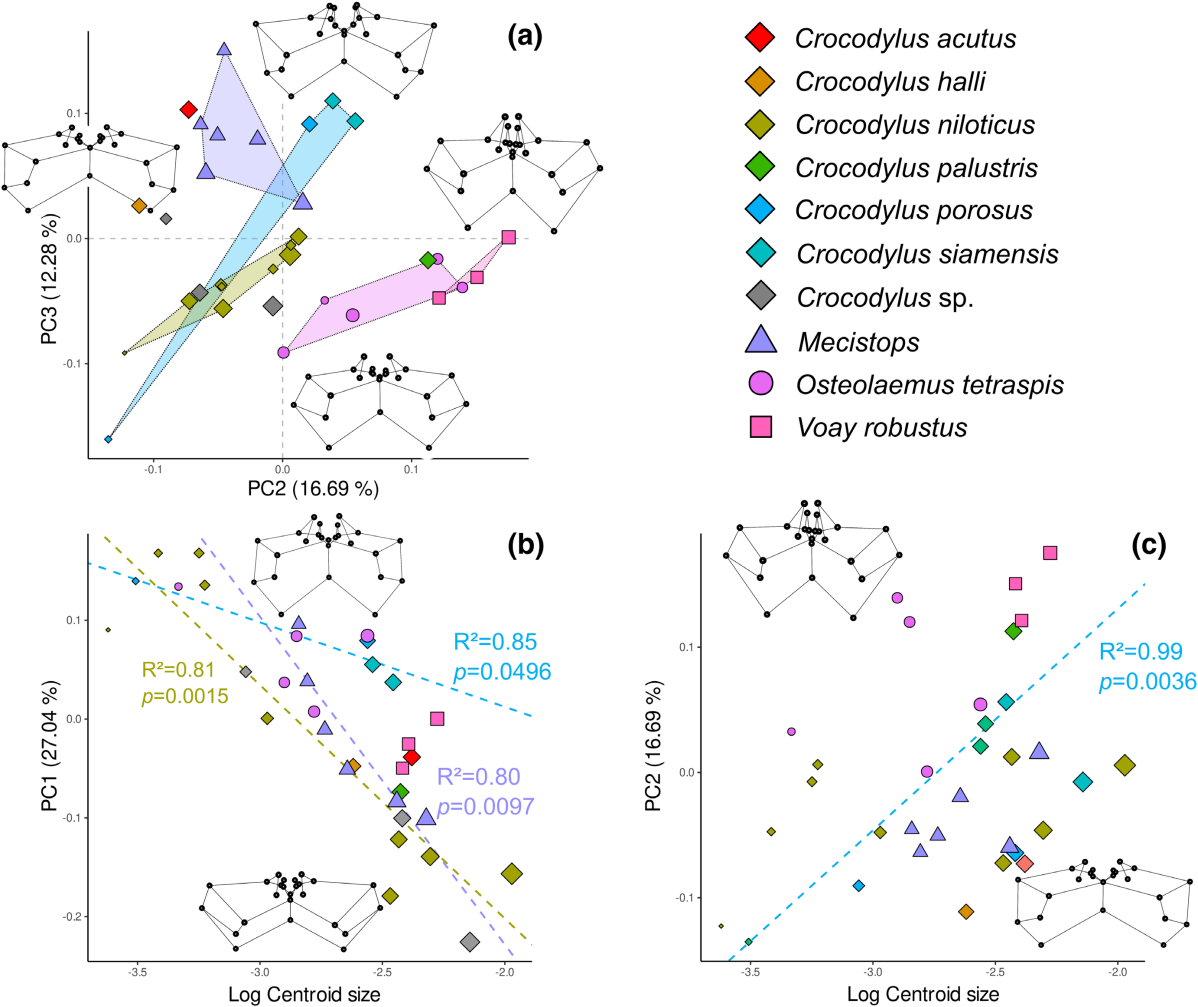
Crocodylidae ontogenetic morphospace of intertympanic sinus and relation to size. (a) PCA results; (b, c) plots of the principal components more correlated with size versus log‐transformed centroid size, associated with the significant ontogenetic trends (regression of the principal components scores vs. log centroid size for each species respectively). Extreme shapes for each PC axis are given with wireframes in anterior view.

Each crocodylid genus displays a distinct morphology, which can be discriminated, for the most part, in the total ontogeny (Figures S12 and S13) and in the Crocodylidae‐only morphospaces (Figure 10, Figure S14). In the latter, the first principal component is only significantly correlated with size (*p* = 0.001, *R* ^2^ = 0.65, Table S4). As such, the morphospace defined by PC2 and PC3, which are both correlated with the species‐specific morphology (*p* = 0.001 and 0.003, *R* ^2^ = 0.68 and 0.51 respectively), better describes the morphological differences between crocodylid species (Figure 10a). PC2‐negative values correspond to small pre‐parietal processes, a dorsoventral reduction of the otoccipital recess and an anteroposterior compression of the prootic part of the intertympanic recess. PC2‐positive values are linked with the development of the parietal recess, a verticalised otoccipital recess and an anteroposteriorly large prootic part of the intertympanic recess. PC3 captures changes in the development of the posteromedial pre‐parietal processes and the verticalisation of the parietal recess, with higher dorsal expansion towards positive values.

We observe that hatchlings cluster in the same region of the morphospace with similar scores on PC1, PC2 and PC3 (Figure 10a, Figure S14). *Mecistops* clusters in PC2‐negative and PC3‐positive values, next to the sub‐longirostrine crocodile *C. acutus*, showing verticalised pre‐parietal processes that flatten with age (displacement towards PC2 and PC3 null values). Furthermore, adult morphology within the genus *Crocodylus* can even be differentiated at the species level (Figure 10a). *Crocodylus niloticus* sits in PC2 and PC3 negative to zero values, in the middle of the morphospace. *Crocodylus porosus* and *C. siamensis* are found close together towards PC2‐ and PC3‐positive values, reflecting their verticalised sinus system and well‐developed parietal recess, which is different from *C. niloticus*. *Crocodylus palustris* is located towards PC2‐positive values, as it displays a greatly developed parietal recess associated with an anteriorly developed prootic part of the intertympanic recess. *Osteolaemus tetraspis* and *V. robustus* are located in PC2‐positive values and PC3 negative to zero values, both displaying a vertical otoccipital recess, an anteriorly protruding prootic part of the intertympanic recess and an anteriorly developed, flat parietal recess.

**FIGURE 11:**
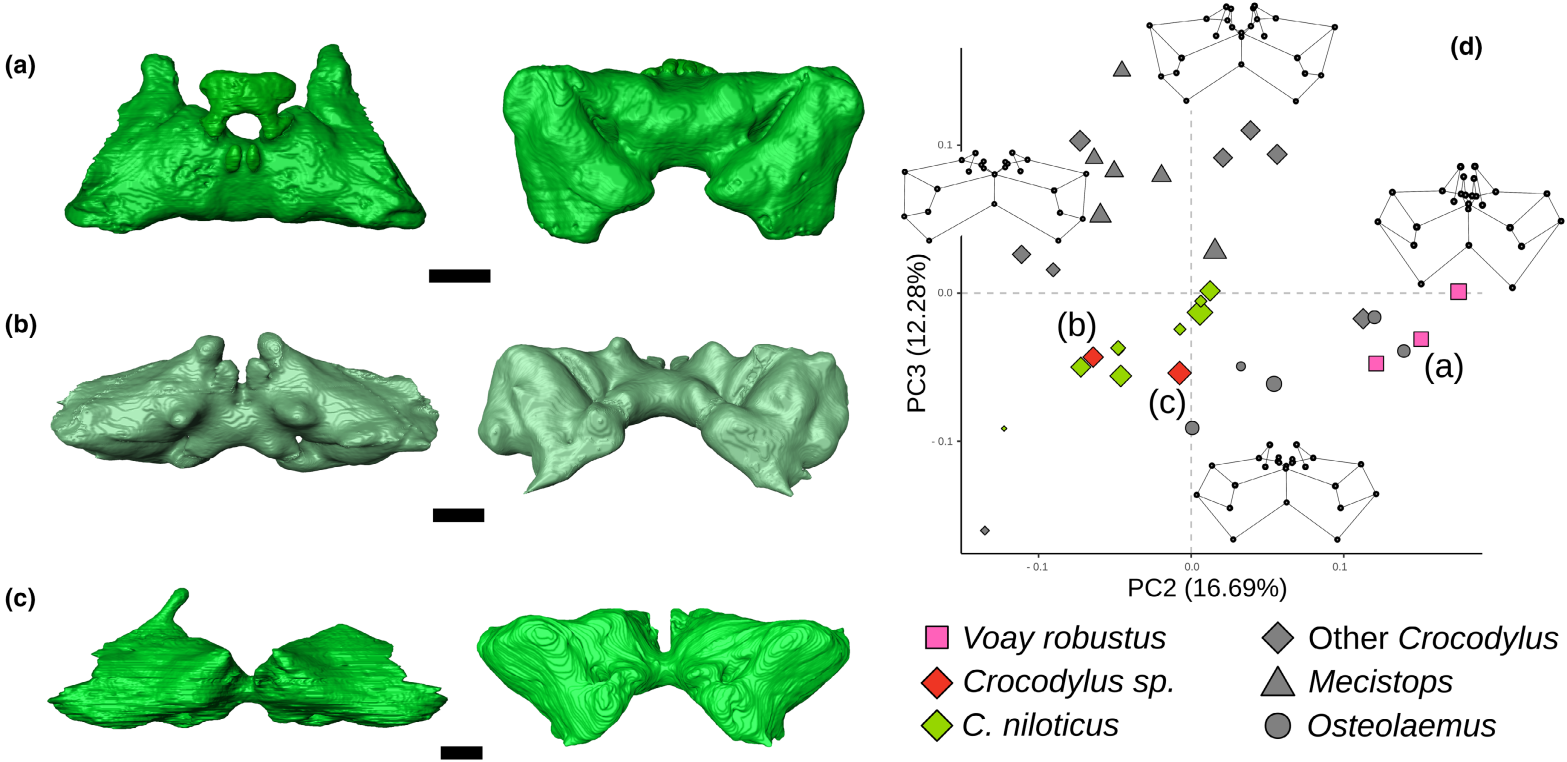
Morphological differentiation of the intertympanic sinus system between the subfossil specimens of *Voay robustus* and *Crocodylus* sp. from Madagascar. (a) *Voay robustus* NHMUK PV R 36684; (b) *Crocodylus* sp. MHNL QV14; (c) *Crocodylus* sp. MNHN 1908‐5‐2, in dorsal (left) and posterior view (right). (d) Ontogenetic morphospace of Crocodylidae from Figure 10. Scale bars = 1 cm.

The two specimens of *Crocodylus* sp. from Madagascar include MHNL QV14, that was already attributed to *Crocodylus* (dated between 7670 and 7510 years cal. BP, Martin et al., 2022), and a new specimen, MNHN 1908‐5‐2 (not yet dated), formerly attributed to *Voay*. Both fall within the *C. niloticus* morphospace area, displaying an anteroposteriorly compressed intertympanic sinus system, a reduced otoccipital recess and poorly developed parietal recess, clearly different from the expanded pneumatic system of *Voay* (Figure 11).

In Crocodylidae, size increase is strongly correlated with the anteroposterior and dorsoventral compression of the intertympanic sinus system captured on PC1, especially in *C. niloticus* and *Mecistops* (Figure 10b). Species allometric modifications behave differently regarding the development of the parietal and otoccipital recesses (Figure 10c and Figure S15). *Crocodylus niloticus* and *Osteolaemus tetraspis* show few morphological changes during the post‐hatching development. *Crocodylus porosus* displays a clearly positive allometric trend on both axes, with a continuous dorsoventral development of the system during growth. *Mecistops* possesses a slightly positive trend on PC2 and a negative trend on PC3, showing a verticalised parietal recess that develops anteriorly but flattens throughout ontogeny. Finally, adult specimens of *C. palustris* and *Voay* are both found in high PC2 values due to the increased anteroposterior development of their intertympanic recess (which is also found in *Osteolaemus* at smaller absolute sizes).

#### 5.1.3 Gavialidae

**FIGURE 12:**
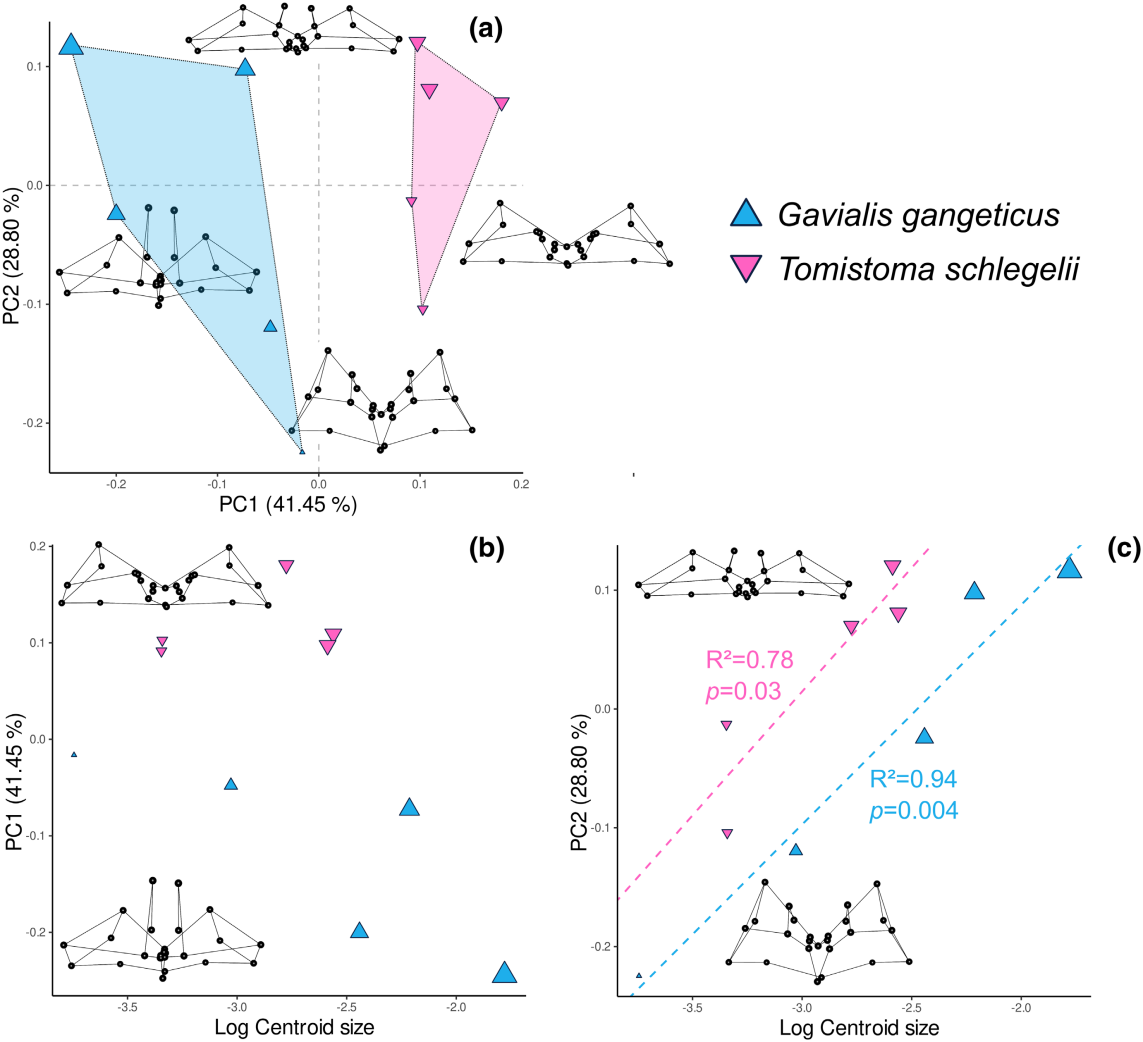
Gavialidae ontogenetic morphospace of intertympanic sinus and relation to size. (a) PCA results; (b, c) plots of the principal components more correlated with size versus log‐transformed centroid size, associated with the significant ontogenetic trends (regression of the principal components scores vs. log centroid size for each species respectively). Extreme shapes for each PC axis are given with wireframes in dorsal view.

The Gavialidae‐only PCA highlights the characters that discriminate *Gavialis* and *Tomistoma* in intertympanic sinus shape (Figure 12a). PC1 is correlated with the species‐specific morphology (*p* = 0.005, *R* ^2^ = 0.58, see Table S4), and separates the small cluster of *Tomistoma* in positive values, characterised by a nearly complete obliteration of the parietal processes, from the more dispersed cluster containing all *Gavialis* specimens in negative values, characterised by an anteriorly and dorsally developed parietal recess.

There is a significant relationship between shape and both size and species individually. However, the interaction term (size: species) was not significant (Table S4), showing that it is not possible to statistically differentiate the ontogenetic trajectories of the two species with only a few specimens, despite their clear separation in the morphospace. Yet, we can see that their difference becomes even more obvious during ontogeny, leading to an easily distinguishable adult sinus morphology (Figures 6, 7, 8, and 12). The two genera display divergent trends on PC1, with *Gavialis* showing a development of the parietal recess, whereas it is reduced in *Tomistoma* (Figure 12b). Size strongly influences the anteroposterior compression of the intertympanic sinus system (*R* ^2^ = 0.67), with both genera possessing a positive allometric trend on PC2. However, for a given size, *Tomistoma* specimens display higher values on PC2, showing a slightly more anteroposteriorly compressed intertympanic sinus than *Gavialis* specimens (Figure 12c).

## 6 DISCUSSION

### 6.1 Development of the intertympanic sinus system during ontogeny

Crocodylians undergo continuous growth throughout most of their life, while their cranial bony features modify as size increases (Kuzmin et al., 2021; Morris et al., 2019, 2021). The growth of the intertympanic sinus system follows this development, as it undergoes shape changes throughout all post‐hatching ontogeny. Important shape variations occur between hatchlings and sub‐adults (about 15 cm in SW, up to 10–25 cm in SL depending on the genus), mostly in the parietal and otoccipital recesses. This is followed by slower changes as absolute size continues to increase, especially the anteroposterior compression of the intertympanic recess.

At minimum size, all hatchlings studied share a similar morphology (shaded area in Figure 7), clustering in the ‘common hatchling region’ (CHR) of the morphospace. Regardless of the species, hatchlings display an undifferentiated intertympanic sinus system with rounded, voluminous intertympanic and otoccipital recesses and a poorly developed parietal recess (Figures 4a,f, 5a,f and 7). The hatchling specimen of *Gavialis* (YPM herr 008438, SW = 2.4 cm) falls in the CHR, sharing a similar morphology with Alligatoridae and Crocodylidae specimens of the same ontogenetic stage. Additionally, when looking at the ontogenetic trends of *Tomistoma* (for which we did not have a hatchling specimen available), we can hypothesise that its origin may be close to the gharial's hatchling position (Figures 7, 8, and 12), or even within the CHR (Figure 7). Further analyses of the *Tomistoma* embryonic and hatching sinus anatomy are needed to test this hypothesis. This observation differs from the ontogenetic course of external cranial and rostral morphology: embryos and hatchlings of *Tomistoma* and *Gavialis* were an exception to the cranial morphospace area containing non‐Gavialidae neonates (the ‘conserved embryonic region’, Morris et al., 2019, 2021), as they both display distinct rostral shapes apart from other Crocodylia early in embryonic development. Here, our results rather echo the developmental pattern of the skull table, which presents a common embryonic trajectory across all extant species, leading to a common morphology of their skull table upon hatching (Morris et al., 2021). Being enclosed in the upper part of the braincase, the intertympanic sinus may follow its pre‐hatching developmental trajectories, leading to shared characteristics and less variability than rostral morphology upon hatching. However, post‐hatching ontogenetic trajectories diverge morphologically.

We observed a common developmental change between hatchlings and juveniles in all species: a dorsoventral thickening of the intertympanic recess and a lateral and posterior flattening of the otoccipital recess (Figures 4a,b,f,g and 5a,b,f,g). These rapid post‐hatching modifications represent a threshold to differentiate neonates and young juveniles from later stages, regardless of absolute size. These changes are likely to be linked with the ‘cranial metamorphosis’ (termed in Tarsitano, 1985; Tarsitano et al., 1989; Kuzmin et al., 2021). This term defines the profound osteological changes occurring rapidly in the post‐hatching development of the crocodylian braincase. It results in the verticalisation of the braincase elements, especially affecting the basisphenoid, basioccipital and pterygoid bones, and to a lesser extent, the quadrate and the exoccipital. Such modifications would necessarily have an impact on the structures encapsulated in the bony elements (Dufeau & Witmer, 2015; Hu et al., 2021). During this developmental event, the ventral part of the exoccipital is slightly affected by the verticalisation while the quadrates develop laterally, causing the changes in orientation of the otoccipital recess after hatching (Figures 4a,b,f,g and 5a,b,f,g). The prootic, parietal and supraoccipital bones are barely impacted by this verticalisation, which would allow the intertympanic and parietal recesses to thicken dorsoventrally while retaining their own ontogenetic trajectories independently of dorsoventral constraints.

Arising from this shared intertympanic sinus shape, post‐hatching individuals of the three families follow different growth trajectories. In Alligatoridae, intertympanic sinus morphology is mainly driven by the anterodorsal development of the parietal recess (Figures 7 and 9). This is remarkably similar to the allometric trend for the hypothetical crocodylian ancestor (Figure 8d). Intertympanic recess shape is weakly modified in *A. mississippiensis* and *C. latirostris* between juveniles and adults, due to the prominent deceleration in compression and the continuous expansion of their diverticula (Figure 8a), resulting in minimal changes in anteroposterior thickness (Figure 4; low dispersion on PC1, Figure 7). This retention of a juvenile morphology is in line with the recognised paedomorphic nature of alligatorid cranial features (Monteiro & Soares, 1997; Morris et al., 2019).

**FIGURE 13:**
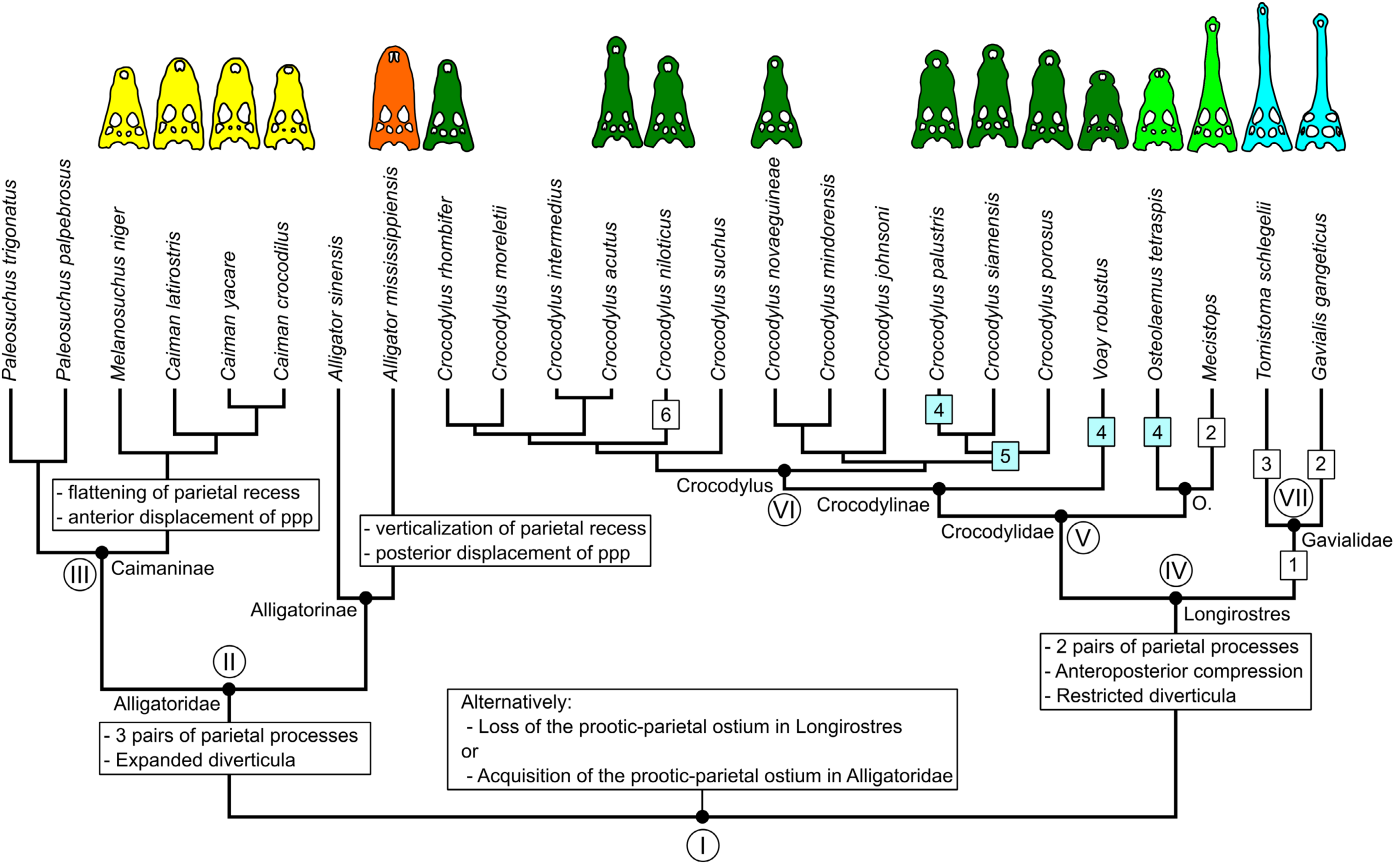
Molecular phylogenetic tree of extant Crocodylia, including *Voay robustus*, associated with skull outlines of each studied species. The tree was constructed as a consensus of the topologies of Oaks & Dudley, 2011; Hekkala et al., 2021; Pan et al., 2021. O., Osteolaeminae; ppp, posteromedial parietal processes; (1) Pre‐displacement of intertympanic sinus anteroposterior compression and reduction of otoccipital recess; (2) Reduction of the posteromedial pre‐parietal processes; (3) Reduction of the anterolateral pre‐parietal processes and absence of parietal recess; (4) Expanded diverticula; (5) Developed and verticalised parietal recess; (6) Reduction of parietal and otoccipital recesses. Characters highlighted in blue correspond to conflicts between the molecular and morphological topologies (Figure S16).

In Longirostres, the intertympanic sinus system displays more profound changes after the cranial metamorphosis, being much less expanded in adults. In Crocodylidae, there is a high disparity in ontogenetic trajectories and resulting adult morphologies. However, the crocodylid sinus ontogeny seems to be partitioned in two phases. From hatchlings to juveniles, they first develop the pre‐parietal processes, overlapping with Alligatoridae morphology (displacement towards PC2‐positive values, Figure 7). Then, from juveniles to adults, they undergo an anteroposterior compression and lateral expansion of the system, which increases with absolute size, constraining the pneumatic cavities (displacement towards PC1‐positive values, Figure 7). This compression mechanism has a different intensity depending on the genus and species: it heavily impacts *C. niloticus* and *Mecistops*, while *C. siamensis* and *C. porosus* are moderately impacted (Figure 8b, Figure S22). *Crocodylus palustris*, *Osteolaemus* and *Voay* are weakly impacted, showing thicker diverticula in adults (Figure 10a). In Indomalayan *Crocodylus* species *C. siamensis*, *C. porosus* and *C. palustris*, the parietal recess also displays an increased rate of development, being greatly developed vertically in adults (Figure 8e, Figure S5) and revealing a common developmental event in this lineage (character 5, Figure 13). Finally, the anteroposterior compression seems to start earlier during the ontogeny of Gavialidae (pre‐displacement at node VII, Figure 8c). This would result in the simultaneous development of the parietal and intertympanic recesses; however, the shift in the calculated trajectory at node VII could be mainly driven by the shift in the intercept of the *Tomistoma* trajectory, which must be taken cautiously in the absence of a hatchling in our dataset. Although they show this common sinus compression pattern, the two genera display a major developmental difference: the acceleration of the parietal recess growth in *Gavialis*, and its deceleration (or even resorption) in *Tomistoma* (Figures 8f and 12b).

While the developmental patterns of the parietal diverticulum seem to be clade‐specific, intertympanic sinus compression seems to be correlated with the variation in skull shape to some extent. Indeed, species characterised by blunt skulls with short and broad rostra, tend to possess anteroposteriorly large and expanded diverticula. Such characteristics are likely to be an ancestral feature of Alligatoridae, but were likely independently acquired by *Osteolaemus* and *Voay*. This tendency is also present to a lesser extent in *C. palustris* (Figure 13). On the other hand, mesorostrine and longirostrine species, that develop more slender skulls with longer and narrower rostra, tend to possess anteroposteriorly compressed and restricted diverticula. This is the case in *C. niloticus*, *C. acutus*, *Mecistops* and the Gavialidae. We hypothesise that these convergences are linked to space availability inside the braincase: during crocodylian cranial growth, the posterior part of the skull table enlarges laterally while the supratemporal fenestrae (STF) become bigger, which reduces room between the latter and the posterior wall of the braincase (Cossette et al., 2021). The size of the STF is linked to the development of the adductor muscles attached to the jaw (i.e. the ‘musculus adductor mandibulae externus profundus’, Holliday & Witmer, 2007), which presents different shapes depending on skull morphotypes. Brevirostrine taxa possess small semi‐circular adductor muscles associated with small STF, while longirostrine taxa possess large circular adductor muscles, associated with larger STF (Holliday & Witmer, 2007). Furthermore, the STF increases in size with snout length in most species: STF development would thus imply an anteroposterior constraint on the intertympanic sinus system, weak in brevirostrine taxa due to smaller STF, but increased in mesorostrine and longirostrine taxa due to the presence of larger STF. The correlation between rostrum and STF sizes, partly controlled by adductor muscle attachments, would thus influence the space available in the braincase for sinus development, linking indirectly rostrum shape and sinus expansion.

In Alligatoridae and *Osteolaemus*, the shape of the skull table remains rectangular during ontogeny, and the STF size remains small (Cossette et al., 2021), reflecting the deceleration of their rostrum‐to‐braincase relative growth (Morris et al., 2019), which concurs with the retention of juvenile characters. The same bone and sinus developmental patterns seem to characterise *Voay*, which displays both a blunt skull and large diverticula (Figure 11). *Crocodylus palustris* also displays an anteriorly developed sinus system, which explains its differentiation from *C. siamensis* and *C. porosus* in the morphospace despite having similar otoccipital and parietal recess characteristics (Figures 10a and 13, Figure S5). In contrast, the already elongated rostrum in *Gavialis* and *Tomistoma* embryos and hatchlings (Morris et al., 2019) may partly explain the earlier onset of sinus compression observed in Gavialidae (Figure 8c). Furthermore, in extant slender‐snouted species (*Mecistops*, *Tomistoma* and *Gavialis*), we observe a substantial reduction of one pair of pre‐parietal processes during their development, in addition to the sinus compression already present in mesorostrine taxa. This reduction could result from further space accommodation in the braincase, due to the longirostrine morphology that indirectly constrains some developmental aspects of the parietal. In extinct taxa, a similar pattern can be observed: the Dyrosauridae possess elongated snouts and very large STF, and a small intertympanic sinus that is greatly reduced anteroposteriorly (Erb & Turner, 2021). As an extreme, the Teleosauridae do not possess any intertympanic sinus (Brusatte et al., 2016; Herrera et al., 2018; Wilberg et al., 2021). These Mesozoic aquatic crocodylomorphs were fully aquatic and characterised by long slender snouts, extremely large STF and an anteroposteriorly restricted braincase.

However, as noted by Cossette et al., 2021, the direct link between longirostry and larger STF is likely to be overstated in some clades, as longirostrine osteolaemines make an exception to this rule. Despite their elongated snout, *Mecistops* and *Euthecodon* both display moderate STF sizes comparable to mesorostrine species. Yet, they are still larger than their osteolaemine counterparts, showing that rostrum shape probably has some influence on STF size, but somehow decoupled from intertympanic sinus shape (adult *Mecistops* having similar compression scores on PC1 as *C. niloticus* or *Gavialis*). This implies that potential additional constraints influence the volume and expansion of intertympanic sinus diverticula.

### 6.2 Is intertympanic sinus shape influenced by ecology?

The sinus system of crocodylians plays an important role in the interaction between the animal and its environment. It is a key part of the hearing process (Carr et al., 2016; Dufeau & Witmer, 2015). Additionally, thanks to its pneumatic nature, it lightens the skull, and may potentially be useful for controlling the buoyancy of the head in an aquatic environment. However, all extant species of Crocodylia share roughly similar ecological preferences, living in semi‐aquatic environments, which makes it difficult to assess environmental‐based differences, and few studies have investigated the behaviour of wild crocodylian species. There are only two genera that represent extremes in behavioural patterns. Among the taxa we sampled, *Osteolaemus* shows the most ‘terrestrial’ lifestyle, living in swamps, secluded pools or stagnant water and was reported to take long walks far from water (Eaton, 2010; Waitkuwait, 1986). In contrast, *Gavialis* shows the most aquatic lifestyle of all extant crocodylians, spending most of its time in water and being the sole extant species to not be able to walk in a semi‐upright stance on land (Stevenson & Whitaker, 2010).

The impact of lifestyle on intertympanic sinus shape was thus tested by partitioning the crocodylian species in three classes (semi‐aquatic for most genera, sub‐aquatic for *Gavialis* and semi‐terrestrial for *Osteolaemus*), revealing a significant correlation (*p* = 0.003), but accounting for only 7.5% of the overall variation (Table S2). Thus, it is likely that intertympanic sinus characteristics were modulated by ecological parameters during the evolution of the crocodylian lineages. However, we were not able to precisely assess which shape changes could be linked to the living environment with our dataset.

In comparison with other crocodylian clades, we observe that the sinus system bears considerable differences between fossil forms from different living environments. For instance, the Sebecidae, a group of Cretaceous–Cenozoic crocodylomorphs that are interpreted to be fully terrestrial, possesses a sinus system that is impressively large and inflated, with numerous diverticula (Pochat‐Cottilloux et al., 2021). On the other hand, in the marine thalattosuchians, the sinus system is compact and reduced, with a complete obliteration of the intertympanic sinus pneumatic recesses (Brusatte et al., 2016; Herrera et al., 2018; Wilberg et al., 2021). We therefore hypothesise that the overall volume of the sinus system, especially the dorsal pneumatic cavities, could be linked to living environments, albeit weakly. In addition to the brevirostry/longirostry constraints mentioned earlier, functional constraints such as head weight and buoyancy could be drivers explaining the selection of sinus volume expansion in more terrestrial species or volume reduction in more aquatic species.

Even so, the shape and arrangement of the pneumatic diverticula, as shown in the present work, are also highly correlated with the species‐specific morphology of the specimens studied, making it useful to assess morphological endocranial characters for studying the crown group.

### 6.3 Relevance of intertympanic sinus morphology for taxonomic determination and phylogenetic analyses

The developmental study of the intertympanic sinus system enables us to examine the ontogenetic trends of different crocodylian genera, leading to different adult sinus morphologies. It now becomes possible to identify characters linked with ontogenetic stages, and highlight the potential diagnostic characters for each studied taxon at adult stage, and eventually, map adult intertympanic sinus morphology in phylogenetic framework (Figure 13, Figure S16). Here, we discuss several key features of crocodylian intertympanic sinuses which could help refine phylogenies in the future or ease the taxonomic attribution of fossil specimens, by including observations based on the sinus morphology as a complementary tool to osteological characters. This is a first step towards the inclusion of endocranial characters in phylogenetic character matrices.

In recent morphological phylogenies, only one character related to the intertympanic sinus system has been used since the works of Brochu: the presence/absence of a parietal recess communicating with the paratympanic pneumatic system, based on longitudinal cutting or low‐resolution CT‐scans (Brochu, 1997, 1999). However, we found that the coding of this character was dependent on ontogenetic variability, and often proved erroneous and inconsistent between publications. Indeed, in the matrices of Brochu (1997, 1999), it corresponds to character 154, stated as present in Alligatoridae and *Gavialis* (0) and absent in *Crocodylus*, *Osteolaemus* and *Tomistoma* (1). Thirteen years later, it corresponds to the character 165 in Brochu et al., 2012, stated as present in *Gavialis*, *Tomistoma* and Crocodylidae (0) while absent in Alligatoridae (1). This confusion affected the subsequent phylogenetic analyses of Rio and Mannion (2021), who used part of Brochu matrices of 2012, coding their character 86 in the same manner but with a correction: *A. mississippiensis* and *C. yacare* are both coded as present (0). However, we have demonstrated that the presence of a recess carving the parietal is a character that is both related to ontogeny and taxonomy, making its use as a discrete character ambiguous. In some species, this character is stable during ontogeny, like in Alligatoridae where it is always present (Figures 4 and 9, Figures S1–S3) and in *Tomistoma*, where it is always absent (Figure 6 and Figure S9). However, in other species, its development depends on the ontogenetic stage. In *Mecistops*, *Osteolaemus*, *Gavialis* and *C. siamensis*, *C. porosus*, *C. palustris*, it is developed from at least the sub‐adult stage. In *C. niloticus*, hatchlings possess a developed parietal recess that resorbs during growth and inflates again in very large specimens (Figure 5a–e). This example highlights the necessity of having a complete understanding of the 3D arrangement of the sinus system with a good insight on the intraspecific and ontogenetic variability, and the need to develop methods to account for ontogenetic changes in phylogenetic matrices.

Despite the changes occurring during ontogeny, intertympanic sinus structures in adult crocodylian skulls show precise morphological differences at the species level. Several of those features corroborate the current phylogenetic framework of the crown clade (Figure 13). First, all Alligatoridae share a third pair of parietal ostia that links the parietal recess anterolaterally to the prootic part of the intertympanic recess (Figure 4). This feature is shared with the three other alligatorid species not studied here (*P. trigonatus*, *P. palpebrosus* and *A. sinensis*), as confirmed in Kuzmin et al. (2021). This additional ostium is absent in all other extant members of Crocodylia; all Longirostres (sensu Harshman et al., 2003) possess only two pairs of pre‐parietal processes, with one pair being reduced during ontogeny in *Mecistops*, *Gavialis* and *Tomistoma* (Figure 13). Thus, it concurs with the recovery of Gavialidae and Crocodylidae as sister‐groups in both molecular (Oaks & Dudley, 2011; Pan et al., 2021; Poe, 1996; Roos et al., 2007; Willis et al., 2007) and recent morphological topologies (Lee & Yates, 2018; Rio & Mannion, 2021). If this pattern is verified in fossil specimens of the three lineages, the presence/absence of the prootic‐parietal ostium might be a key character to discriminate alligatoroids from crocodyloids in the fossil record, if the prootic‐parietal suture is preserved. Whether this character is an apomorphy of Alligatoridae or simply a character loss in Longirostres lineages (Figure 13, Figure S16) could be investigated by examining basal eusuchians and stem Crocodylia representatives.

### 6.4 Alligatorid characters

Alligatoridae are characterised by possessing a third pair of ostia through the prootic‐parietal suture. Within the lineage, Caimaninae and *A. mississippiensis* can be clearly differentiated using intertympanic sinus morphology (Figure 9). Regardless of ontogenetic stage, *A. mississippiensis* possesses a verticalised parietal recess, with posteriorly displaced posteromedial pre‐parietal processes which are oriented dorsally (Figure 4d,e). Caimaninae possesses a flat parietal recess, with anteriorly displaced posteromedial pre‐parietal processes which are oriented anteriorly. The latter are sometimes obliterated in adult *C. yacare*, *C. crocodilus* and *M. niger*, which makes it an ontogenetically sensitive character (Figure 4i,j, Figure S3). The shape of the parietal recess, as well as the relative position of the pre‐parietal processes can still be coded accordingly.

Additionally, Caimaninae species bear further differences with *A. mississippiensis*. Adult *Caiman crocodilus* and *C. yacare* share a remarkably similar morphology, as expected for sister‐taxa (Figure S3). *Caiman latirostris* sinus shape is close to the latter, with a more expanded parietal recess and an inflated intertympanic recess (Figure S2). *Melanosuchus niger* morphology falls close to *Caiman* species in the morphospace, but displays more differentiated diverticula, with more restricted, tubular and well‐separated recesses (Figure 9a, Figure S3). Intertympanic sinus morphology within Alligatoridae thus matches molecular‐based interspecific relationships (Figure 13).

### 6.5 Crocodylid characters

Investigation of the intertympanic sinus system was especially relevant to resolve the issues concerning specific determination within *Crocodylus* species. Indeed, in the absence of provenance information, many curated specimens can be subject to misidentification as their cranial features are very similar, especially regarding *C. niloticus*, *C. porosus* and *C. siamensis* sub‐adult individuals that do not yet possess developed cranial ornamentation. Now, this similarity can partly be overcome by examining internal structures, as implied from the distinctive morphospace occupation of adult taxa (Figure 10, Figure S13). *Crocodylus niloticus* possesses a restricted sinus system showing high anteroposterior compression, poorly developed pre‐parietal processes, and a near complete fusion of the otoccipital recess with the intertympanic recess. *Crocodylus siamensis*, *C. porosus* and *C. palustris* all possess a more expanded sinus system, with a verticalised parietal recess and a differentiated otoccipital recess, including in large individuals. An endocranial morphological differentiation thus occurs between the Indomalayan species and their African counterpart, concurring with molecular‐based biogeographical scenarios (Nicolaï & Matzke, 2019). This partition matches the molecular phylogenetic framework (Figure 13), as Indomalayan species *C. siamensis* and *C. palustris* are retrieved as sister taxa in molecular topologies, followed by *C. porosus* (Pan et al., 2021). Here, adult *C. siamensis* and *C. porosus* form a single cluster; however, as the taxonomic determinations of these specimens may be uncertain, it is impossible to separate the two species without additional specimens. In any case, the biogeographic distribution of these two species greatly overlaps (Nicolaï & Matzke, 2019), and cases of hybridisation between the two species have been reported, and a common sinus morphology is plausible (Fitzsimmons et al., 2002; Lapbenjakul et al., 2017; Simpson & Bezuijen, 2010; Srikulnath et al., 2012). Neotropical crocodiles, on the other hand, are for now insufficiently sampled to draw conclusions on the evolution of their sinus system after their separation from their African ancestors (Delfino et al., 2020; Milián‐García et al., 2020; Nicolaï & Matzke, 2019).

The Madagascar subfossil crocodylian material was also subject to taxonomic misinterpretations. Indeed, the Holocene crocodylian fauna was primarily thought to be only represented by the brevirostrine endemic species *V. robustus*, while the arrival of *Crocodylus* on the island was assumed to be very recent and following the extinction of *Voay* around 2000 years ago (Bickelmann & Klein, 2009; Brochu, 2007; Martin et al., 2022). Consequently, much ancient crocodile material was attributed by default to *V. robustus*. However, Martin et al. (2022) showed, using a combination of external and internal cranial characters, that the occurrence of *Crocodylus* on the island was at least contemporaneous with the endemic species, and that Malagasy crocodylian material needed to be re‐evaluated (Martin et al., 2022). Here, we confirm the distinctive morphological gap between ‘true’ *V. robustus* specimens and two subfossil *Crocodylus* sp. from Madagascar (Figure 11). Agreeing with the absence of *Voay* diagnostic traits (pronounced squamosal horns and verticalised snout), the intertympanic sinus of these subfossils of *Crocodylus* sp. share the same morphology as *C. niloticus* (Figure 11), that is, a compressed intertympanic sinus, an absence of a developed prootic part, and reduced otoccipital and parietal recesses. This shows that the Malagasy *Crocodylus* internal morphology is easily distinguishable from that of *V. robustus*. Furthermore, it implies that the oldest *Crocodylus* populations of Madagascar were probably morphologically associated with African *C. niloticus* populations coming from the African mainland (Hekkala et al., 2021).

Additionally, *V. robustus* displays a sinus morphology that is close to *Osteolaemus*: an anteroposteriorly developed intertympanic recess with a protruding prootic part, linked ventrally to the prootic facial recess; an expanded and verticalised otoccipital recess with no lateral compression; and a flat parietal recess developed anteriorly, laterally and posteriorly (Figures 10 and 11, Figures S6 and S10). These shared characters are in line with their proposed close relationship based on morphological phylogenies (Figure S16; Brochu, 2007; Rio & Mannion, 2021), but contrast with molecular results which retrieve *Voay* as a stem‐taxon to the *Crocodylus* lineage (Hekkala et al., 2021). On the other hand, *Mecistops*, which is considered as the sister‐taxon to *Osteolaemus* in the molecular framework, possess a different and compressed morphology similar to that of Crocodylinae (especially to the sub‐longirostrine *C. acutus*), which rather reflects once again phylogenetic topologies derived from morphological characters, where *Mecistops* often lies at the base of the *Crocodylus* lineage (Figure S16). *Voay*, *Osteolaemus* and *Mecistops* are an example of high disparity in rostrum shape in a putatively single lineage, associated with two very different types of sinus system. In any case, even if the influence of cranial shape on endocranial features is different in Osteolaeminae, it has not overwritten shared sinus characters in *Voay* and *Osteolaemus*. This reinforces the problematic status of osteolaemines, a group that is still phylogenetically debated and composed of morphologically distant taxa. In the future, the investigation of juveniles and fossil osteolaemine relatives may help clarify such questions.

### 6.6 Gavialoid characters

The similarity in intertympanic sinus ontogeny in *Tomistoma* and *Gavialis* may corroborate their shared ancestry, shown by a common pre‐displacement event of sinus compression occurring in the Gavialidae lineage (Figures 8c and 13, Figure S16). This is congruent with the evolution of their cranial ontogeny, because their cranial development also underwent a pre‐displacement of both snout elongation and braincase size reduction sometime before the *Gavialis*‐*Tomistoma* evolutionary split (Morris et al., 2019). Both aspects thus reinforce the phylogenetic placement of *Tomistoma* and *Gavialis* as sister‐taxa. Furthermore, their seemingly divergent ontogenetic trajectories are consistent with the rapid evolutionary rates retrieved for the gharial lineage, which draws *Gavialis* morphology far away from its sister‐taxon *Tomistoma* in less than 10 Ma after their separation, around 38 Ma according to tip‐dated molecular‐clock estimates (Lee & Yates, 2018). However, most morphology‐based phylogenetic methods still retrieve divergence time between Tomistominae and Gavialinae around 100 Ma. This is mainly due to the ‘thoracosaur’ issue, which involves longirostrine taxa that are historically considered as stem‐gavialoids and are still retrieved as such in the recent morphological topologies (Rio & Mannion, 2021). Along with other longirostrine fossils, ‘thoracosaurs’ are convergent with modern gharials due to atavism and reversion of cranial characters in the latter (Gatesy et al., 2003; Lee & Yates, 2018). Their age (from Late Cretaceous to early Palaeocene) indicates the presence of tens of millions of years‐long ghost lineages in the branches leading to extant Gavialidae in morphological‐based topologies (Rio & Mannion, 2021). Therefore, it would be extremely useful to have an insight on thoracosaur internal structures to investigate whether their gharial‐like external morphology is independent of their sinus structure, the latter being a potential new morphological proxy to separate these ancient forms from modern gharials.

Finally, the morphological differentiation observed between *Gavialis* and *Tomistoma* bears implications among fossil relatives. If the intertympanic morphological distinctiveness is conserved in the braincase of extinct gavialines and tomistomines, important taxonomic implications are expected within longirostrine forms, circumventing the phylogenetic convergence in snout shape. Supporting this view, the neuroanatomy of *Gryposuchus neogaeus*, a Miocene longirostrine crocodylian considered part of the gavialid lineage, represents an interesting case (Bona et al., 2015). Many differences in the shape and extension of the paratympanic sinuses were reported between the specimen and *Gavialis gangeticus*. Notably, the intertympanic sinus system was well preserved, and showed several recognisable features: a considerable compression of the medial part of the intertympanic recess; a ventral reduction of the otoccipital recess; and the absence of any parietal recesses. This morphology is more reminiscent of *Tomistoma* than *Gavialis*, which could potentially point towards a closer relationship of *Gryposuchus* with Tomistominae, or a basal position before the gavialid split. Indeed, many longirostrine specimens display a mosaic of longirostrine characters which complicates the phylogenetic attributions, such as *Thoracosaurus*, *Eosuchus* or younger taxa like *Gavialosuchus* (Lee & Yates, 2018; Rio & Mannion, 2021). The examination of their neuroanatomy and the establishment of endocranial‐based character matrices thus becomes mandatory to refine their phylogenetic attributions. Other examples include *Hanyusuchus sinensis*, *Toyotamaphimeia machikanensis* and *Penghusuchus pani*, East Asian longirostrine fossil taxa (respectively, from the Bronze Age, the Pleistocene and the Miocene). They were described as displaying a combination of tomistomine and gavialine characters, bringing further support for the sister taxon relationships of the two lineages (Iijima et al., 2022; Iijima & Kobayashi, 2019). Therefore, the investigation of the intertympanic sinus of such longirostrine taxa holds important promises to understand the evolution of the internal organs in Gavialidae and longirostrine crocodylians in general.

## 7 CONCLUSION

The investigation of crocodylian intertympanic sinus systems revealed that endocranial structures bear species‐specific differences, which corroborate molecular data at high‐ and low‐level phylogenetic relationships in extant taxa. Alligatoridae are clearly differentiated from Longirostres by the absence of a prootic‐parietal ostium in the latter. Sinus post‐hatching development begins with a common hatchling shape shared by all studied species, from which ontogenetic trajectories diverge, following different heterochronic modifications resulting in distinct adult characteristics. Sinus anteroposterior compression seems to have been accelerated and pre‐displaced in mesorostrine and longirostrine species, which we link to cranial constraints due to enlarged STF in taxa with an elongated rostrum. In adults, species are mainly separated by differences in the shape of the otoccipital and parietal recesses, which can now be used to differentiate *Caiman* from *Alligator*, Indomalayan from African *Crocodylus*, *C. niloticus* from *Voay*, as well as *Tomistoma* from *Gavialis*. These characteristics can be used to accurately discuss the evolution of endocranial sinuses in extinct crocodylians with respect to extant species, and eventually bring more material for the taxonomic attributions of fossil eusuchian representatives by integrating sinus characters in morphological matrices. This could help resolve some debates regarding the phylogenetic attributions of basal crocodylians or fossil representatives of the Gavialidae lineage. Further investigations are however still needed on juveniles and fossil forms to untangle the cranial and ecological constraints influencing sinus shape throughout Crocodylia evolution.

## AUTHOR CONTRIBUTIONS

JM, GP: Conceptualisation. GP, CS, BD, YP, JM, IR, NR: Investigation. GP, YP, JM, CS, NR: Data Curation. GP, LH: Formal Analysis. GP, JM, YP, LH: Methodology. JA, JL, VF, IR: Resources. GP: Visualisation. GP, YP, JM, VF: Validation. JM and LH: Supervision. GP: Writing—original draft. GP, JM, LH, YP, IR, VF: Writing—review and editing.

## Supporting information


Data S1.

Data S2.

Data S3.

Data S4.

Data S5.

Data S6.

Data S7.

Data S8.

Data S9.

Data S10.

Data S11.

Data S12.

Figure S1.
Figure S2.
Figure S3.
Figure S4.
Figure S5.
Figure S6.
Figure S7.
Figure S8.
Figure S9.
Figure S10.
Figure S11.
Figure S12.
Figure s13.
Figure S14.
Figure S15.
Figure S16.
Figure S17.
Figure S18.
Figure S19.
Figure S20.
Figure S21.
Figure S22.

Table S1.
Table S2.
Table S3.
Table S4.
Table S5.
Table S6.
Table S7.

## Data Availability

All reconstructed volumes coming from French and UK curated specimens can be accessed on the online repository MorphoMuseum (Perrichon et al., 2022). Raw data of specimens from the SMNK were provided by Irena Raselli and will be published elsewhere. The rest of the raw data can be obtained from the online repositories Morphosource or Digimorph. R scripts are provided in the supplementary materials.
