# Supplementary material for: Ontogenetic variability of the intertympanic sinus distinguishes lineages within Crocodylia
Source: J Anat. 2023 Jan 29;242(6):1096–123. doi: 10.1111/joa.13830 (PMC10184552; doi:10.1111/joa.13830)
Supplement: Supplementary file 1 — Data S1. [file JOA-242-1096-s001.pdf]

CT information

Specimen number	Taxonomic identification	instrument	voltage	current	filtration	exposure time	number of projections	frame averaging	Voxel size	Post treatment
OUVC 10606	*Alligator mississippiensis*	GE eXplore Locus in vivo	80 kV	450 µA		not provided	1200	7	45^3 µm^3	ImageJ: 16bit to 8bit; contrast; scale 90.55 to 181.1µm
OUVC 11415	*Alligator mississippiensis*	GE eXplore Locus in vivo	120 kV	32000 µA	Al	0.020 s	1200	4	49^3 µm^3	ImageJ: 16bit to 8bit; contrast; crop
OUVC 9761	*Alligator mississippiensis*	GE Hi Speed FX-I Helical CT scanner	120 kV	160000 µA	bowtie	*not provided*	*not provided*	*not provided*	500 x 500 x 1000 µm^3	ImageJ: 16bit to 8bit; contrast
SMNK-REP 164	*Alligator mississippiensis*	*not provided*	*not provided*	*not provided*	*not provided*	*not provided*	*not provided*	*not provided*	29^3 µm^3	ImageJ: 16bit to 8bit; contrast
SMNK-REP 308	*Alligator mississippiensis*	*not provided*	*not provided*	*not provided*	*not provided*	*not provided*	*not provided*	*not provided*	79^3 µm^3	ImageJ: 16bit to 8bit; contrast; crop
SMNK-REP 309	*Alligator mississippiensis*	*not provided*	*not provided*	*not provided*	*not provided*	*not provided*	*not provided*	*not provided*	79^3 µm^3	ImageJ: 16bit to 8bit; contrast; crop
SMNK-REP 311	*Alligator mississippiensis*	*not provided*	*not provided*	*not provided*	*not provided*	*not provided*	*not provided*	*not provided*	79^3 µm^3	ImageJ: 16bit to 8bit; contrast; crop
TMM M-983	*Alligator mississippiensis*	Scientific Measurement Systems 101	*not provided*	*not provided*	*not provided*	*not provided*	*not provided*	*not provided*	250 x 250 x 480 µm^3	ImageJ: 16bit to 8bit; contrast
UCBL-W835	*Alligator mississippiensis*	GE Phoenix v|tome|x s	140 kV	80 µA	none	0.667 s	1500	4	70^3 µm^3	ImageJ: 16bit to 8bit; contrast
ummz herps 128024	Caiman crocodilus	Nikon Metrology XT H 225 ST	85 kV	200 µA	none	0.250 s	1601	2	121^3 µm^3	ImageJ: 16bit to 8bit; contrast; 121µm to 242µm; crop; rotation
ummz herps 155282	Caiman crocodilus	Nikon Metrology XT H 225 ST	85 kV	200 µA	none	0.250 s	1601	2	68^3 µm^3	ImageJ: 16bit to 8bit; contrast; crop
ummz herps 46112	Caiman crocodilus	Nikon Metrology XT H 225 ST	85 kV	200 µA	none	0.250 s	1601	2	121^3 µm^3	ImageJ: 16bit to 8bit; contrast; rotation; crop
SMNK-REP 314	*Caiman latirostris*	*not provided*	*not provided*	*not provided*	*not provided*	*not provided*	*not provided*	*not provided*	29^3 µm^3	ImageJ: 16bit to 8bit; contrast; crop
SMNK-REP 315	*Caiman latirostris*	*not provided*	*not provided*	*not provided*	*not provided*	*not provided*	*not provided*	*not provided*	29^3 µm^3	ImageJ: 16bit to 8bit; contrast; crop
SMNK-REP 316	*Caiman latirostris*	*not provided*	*not provided*	*not provided*	*not provided*	*not provided*	*not provided*	*not provided*	29^3 µm^3	ImageJ: 16bit to 8bit; contrast; crop
SMNK-REP 317	*Caiman latirostris*	*not provided*	*not provided*	*not provided*	*not provided*	*not provided*	*not provided*	*not provided*	29^3 µm^3	ImageJ: 16bit to 8bit; contrast; crop
ummz herps 155283	*Caiman latirostris*	Nikon Metrology XT H 225 ST	85 kV	200 µA	none	0.250 s	1601	2	90^3 µm^3	ImageJ: 16bit to 8bit; contrast; crop
ummz herps 155284	*Caiman latirostris*	Nikon Metrology XT H 225 ST	85 kV	200 µA	none	0.250 s	1601	2	48^3 µm^3	ImageJ: 16bit to 8bit; contrast; 47,5µm to 95µm
ummz herps 155285	*Caiman latirostris*	Nikon Metrology XT H 225 ST	85 kV	200 µA	none	0.250 s	1601	2	66^3 µm^3	ImageJ: 16bit to 8bit; contrast; decoupe
ummz herps 155286	*Caiman latirostris*	Nikon Metrology XT H 225 ST	85 kV	200 µA	none	0.250 s	1601	2	109^3 µm^3	ImageJ: 16bit to 8bit; contrast; crop
ummz herps 155287	*Caiman latirostris*	Nikon Metrology XT H 225 ST	85 kV	200 µA	none	0.250 s	1601	2	104^3 µm^3	ImageJ: 16bit to 8bit; contrast; suppression slide; 103,8µm to 207,6 µm
ummz herps 155288	*Caiman latirostris*	Nikon Metrology XT H 225 ST	85 kV	200 µA	none	0.250 s	1601	2	100^3 µm^3	ImageJ: 16bit to 8bit; contrast
ummz herps 155289	*Caiman yacare*	Nikon Metrology XT H 225 ST	85 kV	200 µA	none	0.250 s	1601	2	121^3 µm^3	ImageJ: 16bit to 8bit; contrast; suppression slide; 121µm to 242µm
MZS Cro055	*Crocodylus acutus*	GE Phoenix v|tome|x s	140 kV	80 µA	none	0.667 s	1500	3	87^3 µm^3	ImageJ: 16bit to 8bit; contrast.
UCBL 2019-1-237	*Crocodylus cf porosus*	GE Phoenix v|tome|x s	140 kV	80 µA	none	1.000 s	1500	4	75^3 µm^3	ImageJ: 16bit to8bit; contrast
uf herp 145927	*Crocodylus halli*	GE Phoenix v|tome|x m 240	150 kV	200 µA	Cu 0.5 mm	0.250 s	2200	3	112^3 µm^3	ImageJ: 16bit to 8bit; contrast; 112 µm to 224 µm
ag SVSTUA 022002	*Crocodylus niloticus*	RX Solutions Double Tomographe Haute Energie (DTHE)	280 kV	301 µA	none	0.111 s	1504	7	90^3 µm^3	ImageJ: 16 bit to 8 bit; contrast; crop; Res=90µm
MHNL 50001387	*Crocodylus niloticus*	GE Phoenix v|tome|x s	140 kV	80 µA	none	0.667 s	1500	3	86^3 µm^3	ImageJ: 16bit to8bit; contrast; 86 to 172µm
MHNL 50001388	*Crocodylus niloticus*	GE Phoenix v|tome|x s	140 kV	80 µA	none	1.000 s	1500	4	82^3 µm^3	ImageJ: 16bit to 8bit;contrast
MHNL 50001397	*Crocodylus niloticus*	RX Solutions Double Tomographe Haute Energie (DTHE)	290 kV	294 µA	none	0.111 s	2240	3	90^3 µm^3	ImageJ: 16bit to8bit; contrast; 90µm to 180µm
MHNL 50001405	*Crocodylus niloticus*	RX Solutions Double Tomographe Haute Energie (DTHE)	290 kV	340 µA	none	0.167 s	2240	3	102^3 µm^3	ImageJ: 16bit to8bit; contrast; 102µm to 178.5µm
UCBL-FSL 532077	*Crocodylus niloticus*	GE Phoenix v|tome|x s	120 kV	120 µA	none	0.667 s	720	3	86^3 µm^3	ImageJ: 16bit to8bit; contrast
UM 2001-1756-1-434NR	*Crocodylus niloticus*	RX Solutions EasyTom 150	80 kV	380 µA	Al 1 mm	*not provided*	1440	7	78^3 µm^3	ImageJ: 16bit to 8bit; contrast; crop 78µm to 156µm
MHNL 90001850	*Crocodylus niloticus/suchus*	GE Phoenix v|tome|x s	120 kV	120 µA	none	0.500 s	1200	3	50^3 µm^3	ImageJ: 16bit to 8bit;contrast
MHNL 90001851	*Crocodylus niloticus/suchus*	GE Phoenix v|tome|x s	140 kV	80 µA	none	0.500 s	1200	3	50^3 µm^3	NA
MHNL 90001855	*Crocodylus niloticus/suchus*	GE Phoenix v|tome|x s	140 kV	80 µA	none	0.500 s	1200	3	60^3 µm^3	NA
MHNL 50001398	*Crocodylus palustris*	RX Solutions Double Tomographe Haute Energie (DTHE)	290 kV	329 µA	*not provided*	*not provided*	2240	3	102^3 µm^3	ImageJ: 16bit to 8bit; contrast; scale 102.368 µm to 204.736 µm
OUVC 10899	*Crocodylus porosus*	GE eXplore Locus in vivo	120 kV	32000 µA	Al	0.020 s	1200	7	47^3 µm^3	ImageJ: 16bit to 8bit; contrast; crop
MHNL 42006506	*Crocodylus rhombifer*	GE Phoenix v|tome|x s	120 kV	120 µA	none	0.500 s	1200	5	86^3 µm^3	ImageJ: 16bit to8bit; contrast
MHNL 42006507	*Crocodylus rhombifer*	GE Phoenix v|tome|x s	120 kV	120 µA	none	0.500 s	1200	5	86^3 µm^3	ImageJ: 16bit to8bit; contrast
MHNL 50001389	*Crocodylus siamensis*	GE Phoenix v|tome|x s	140 kV	80 µA	*not provided*	1.000 s	1500	4	72^3 µm^3	ImageJ: 16bit to 8bit;contrast; 72 to 144µm
UCBL-W841	*Crocodylus siamensis*	GE Phoenix v|tome|x s	140 kV	80 µA	none	1.000 s	1500	4	86^3 µm^3	ImageJ: 16bit to 8bit; contrast; 86µm to 172µm
MHNL QV14	*Crocodylus* sp	GE Phoenix v|tome|x s	140 kV	80 µA	*not provided*	0.667 s	1000	3	80^3 µm^3	ImageJ:16bit to 8bit; contrast; 80µm to 160µm; scan assembly and reorientation
MNHN-F.1908-5-2	*Crocodylus* sp	RX Solutions Double Tomographe Haute Energie (DTHE)	290 kV	319 µA	none	0.333 s	2240	3	102^3 µm^3	ImageJ:16bit to 8bit; contrast; 102µm to 178.5µm
MHNL 50001407	*Gavialis gangeticus*	RX Solutions Double Tomographe Haute Energie (DTHE)	290 kV	319 µA	none	0.167 s	2240	3	102^3 µm^3	ImageJ: 16bit to 8bit;contrast; 102µm to 178.5µm
NHMUK 1846.1.7.3	*Gavialis gangeticus*	Nikon XT H 225 ST - rotating target	120 kV	415 µA	Cu 0.5 mm	0.250 s	4401	2	31^3 µm^3	ImageJ: 16bit to 8bit; contrast; scale 31.25 to 62.5µm
NHMUK 1873	*Gavialis gangeticus*	Nikon XT H 225 ST - rotating target	200 kV	650 µA	Cu 2 mm	0.250 s	4476	2	52^3 µm^3	ImageJ: 16bit to 8bit; contrast; scale 52.44 to 104.87µm
uf herp 118998	*Gavialis gangeticus*	GE Phoenix v|tome|x m 240	120 kV	200 µA	Cu 0.1 mm	0.200 s	2000	3	147^3 µm^3	ImageJ: 16bit to 8bit; contrast; 146,5 µm to 293 µm
ypm herr 008438	*Gavialis gangeticus*	Research, Inc) ACTIS CT scanner with FeinFocus X-ray source	200 kV	180 µA	none	*not provided*	1400	2	25.39 x 55.73 µm^3	ImageJ: 16bit to 8bit;contrast
ag SVSTUA 022001	*Mecistops* sp.	GE Phoenix v|tome|x s	140 kV	80 µA	none	0.667 s	900	3	86^3 µm^3	ImageJ: 16bit to 8bit, constrast; 86µm to 172µm; rotation 180°
AMU-Zoo-04721	*Mecistops* sp.	GE Phoenix v|tome|x s	140 kV	80 µA	none	0.667 s	900	3	86^3 µm^3	ImageJ: 16bit to 8bit, constraste; 86µm to 172µm; rotation 180°
MHNL 50001393	*Mecistops* sp.	GE Phoenix v|tome|x s	120 kV	120 µA	none	0.500 s	1500	3	72^3 µm^3	ImageJ: 16bit to 8bit;contrast
MZS Cro083	*Mecistops* sp.	GE Phoenix v|tome|x s	140 kV	80 µA	none	0.665 s	1500	5	80^3 µm^3	ImageJ: 16bit to8bit; contrast
NHMUK 1924.5.10.1	*Mecistops* sp.	Nikon XT H 225 ST - rotating target	200 kV	800 µA	Cu 2 mm	0.250 s	4476	2	91^3 µm^3	ImageJ: 16bit to 8 bit,; contrast; scale 67.4 to 134.8µm
UM N89	*Mecistops* sp.	GE Phoenix v|tome|x s	140 kV	80 µA	none	1.000 s	1200	3	86^3 µm^3	ImageJ: 16bit to 8bit, constraste
MZS Cro073	*Melanosuchus niger*	GE Phoenix v|tome|x s	140 kV	80 µA	none	0.667 s	1500	3	87^3 µm^3	ImageJ: 16bit to 8bit; contrast
fmnh 98936 M63578-114918	*Osteolaemus tetraspis*	Research, Inc) ACTIS CT scanner with FeinFocus X-ray source	180 kV	133 µA	none	*not provided*	1400	2	54.7 x 54.7 x 110.8 µm^3	ImageJ: 16bit to 8bit; contrast
MHNM.9095.0	*Osteolaemus tetraspis*	RX Solutions Double Tomographe Haute Energie (DTHE)	180 kV	427 µA	none	0.167 s	2000	3	85^3 µm^3	ImageJ: 16bit to 8bit; contrast
MZS Cro040	*Osteolaemus tetraspis*	GE Phoenix v|tome|x s	120 kV	120 µA	none	0.500 s	1500	3	55^3 µm^3	ImageJ: 16bit to8bit; contrast
NHMUK 1862.6.30.5	*Osteolaemus tetraspis*	Nikon XT H 225 ST - rotating target	200 kV	800 µA	Cu 2 mm	0.250 s	4476	2	54^3 µm^3	ImageJ: 16bit to 8bit; contrast; scale 31.25 to 62.5µm
UCBL-2019-1-236	*Osteolaemus tetraspis*	GE Phoenix v|tome|x s	140 kV	80 µA	none	0.667 s	1500	4	70^3 µm^3	ImageJ: 16bit to8bit; contrast
fmnh 98874	*Tomistoma schlegelii*	Research, Inc) ACTIS CT scanner with FeinFocus X-ray source	180 kV	133 µA	none	*not provided*	1400	2	65.4 x 65.4 x 140.8 µm^3	ImageJ: 16bit to 8bit;contrast
MZS Cro094	*Tomistoma schlegelii*	GE Phoenix v|tome|x s	140 kV	80 µA	none	0.665 s	1500	3	86^3 µm^3	ImageJ: 16bit to 8bit; contrast
NHMUK 1893.3.6.14	*Tomistoma schlegelii*	Nikon XT H 225 ST - rotating target	200 kV	800 µA	Cu 2 mm	0.250 s	4476	2	67^3 µm^3	ImageJ: 16bit to 8 bit; contrast; rotation; scale 67.4 to 134.8µm
TMM M6342	*Tomistoma schlegelii*	*not provided*	420 kV	1800 µA	none	0.032 s	1000		165 x 165 x 1 460 µm^3	ImageJ: 16bit to 8bit; contrast
UM1097	*Tomistoma schlegelii*	RX Solutions EasyTom 150	100 kV	290 µA	Al 1 mm	*not provided*	1440	8	100^3 µm^3	ImageJ: 16bit to 8bit; contrast; crop; scale 100 to 200 µm
MNHN-F.1908-5	*Voay robustus*	*not provided*	150 kV	500 µA	*not provided*	*not provided*	*not provided*	*not provided*	133^3 µm^3	ImageJ: 16bit to 8bit; contrast
NHMUK-PV-R-36684	*Voay robustus*	Nikon XT H 225 ST - rotating target	180 kV	467 µA	Sn 1 mm	0.354 s	3699	2	91^3 µm^3	ImageJ:16bit to 8bit; contrast; scale to 181µm
NHMUK-PV-R-36685	*Voay robustus*	Nikon XT H 225 ST - rotating target	180 kV	889 µA	Sn 1 mm	0.500 s	3699	2	91^3 µm^3	ImageJ:16bit to 8bit; contrast; scale to 181µm
