# Supplementary material for: Ontogenetic variability of the intertympanic sinus distinguishes lineages within Crocodylia
Source: J Anat. 2023 Jan 29;242(6):1096–123. doi: 10.1111/joa.13830 (PMC10184552; doi:10.1111/joa.13830)
Supplement: Supplementary file 13 — Figure S1. Figure S2. Figure S3. Figure S4. Figure S5. Figure S6. Figure S7. Figure S8. Figure S9. Figure S10. Figure S11. Figure S12. Figure s13. Figure S14. Figure S15. Figure S16. Figure S17. Figure S18. Figure S19. Figure S20. Figure S21. Figure S22. [file JOA-242-1096-s002.pdf]

Ontogenetic variability of the intertympanic sinus distinguishes lineages within Crocodylia

Gwendal PERRICHON, Lionel HAUTIER, Yohan POCHAT-COTTILLOUX, Irena RASELLI, Céline SALAVIALE, Benjamin DAILH, Nicolas RINDER, Vincent FERNANDEZ, Jérôme ADRIEN, Joël LACHAMBRE, Jeremy E. MARTIN

Supplementary Figures

Fig. S1. 3D renderings of the intertympanic sinus system of all studied specimen of *Alligator mississippiensis*.

Fig. S2. 3D renderings of the intertympanic sinus system of all studied specimen of *Caiman latirostris*.

Fig. S3. 3D renderings of the intertympanic sinus system of all studied specimen of *Caiman crocodilus*, *Caiman yacare*, and *Melanosuchus niger*.

Fig. S4. 3D renderings of the intertympanic sinus system of all studied specimen of *Crocodylus niloticus*.

Fig. S5. 3D renderings of the intertympanic sinus system of studied specimen of *Crocodylus* species apart from *C. crocodylus*.

Fig. S6. 3D renderings of the intertympanic sinus system of all studied specimen of *Osteolaemus tetraspis*.

Fig. S7. 3D renderings of the intertympanic sinus system of all studied specimen of *Mecistops* sp.

Fig. S8. 3D renderings of the intertympanic sinus system of all studied specimen of *Gavialis gangeticus*.

Fig. S9. 3D renderings of the intertympanic sinus system of all studied specimen of *Tomistoma schlegelii*.

Fig. S10. 3D renderings of the intertympanic sinus system of all studied specimen of *Voay robustus*.

Fig. S11. Ontogenetic morphospace of Crocodylia with subfamilies represented.

Fig. S12. Ontogenetic morphospace of Crocodylia with genera represented.

Fig. S13. Ontogenetic morphospace of Crocodylia with species represented.

Fig. S14. Morphospace of the first two principal components of the Crocodylidae-only PCA.

Fig. S15. PC3 of the Crocodylidae-only PCA versus Log Centroid Size of specimens.

Fig. S16. Morphological phylogenetic tree of extant Crocodylia, including *Voay robustus*, associated with skull outlines of each studied species.

Fig. S17. PC1 scores of the total ontogeny morphospace against Log Centroid Size.

Fig. S18. PC2 scores of the total ontogeny morphospace against Log Centroid Size.

Fig. S19. Molecular tree topology used for ancestral state estimation of ontogenetic trajectory coefficients.

Fig. S20. Ancestral state reconstruction of PC1 ontogenetic trajectories mapped on the molecular topology.

Fig. S21. Ancestral state reconstruction of PC2 ontogenetic trajectories mapped on the molecular topology.

Fig. S22. Reconstructed PC1 and PC2 ontogenetic trajectories of Osteolaeminae with estimated ontogenetic trends at ancestral nodes.

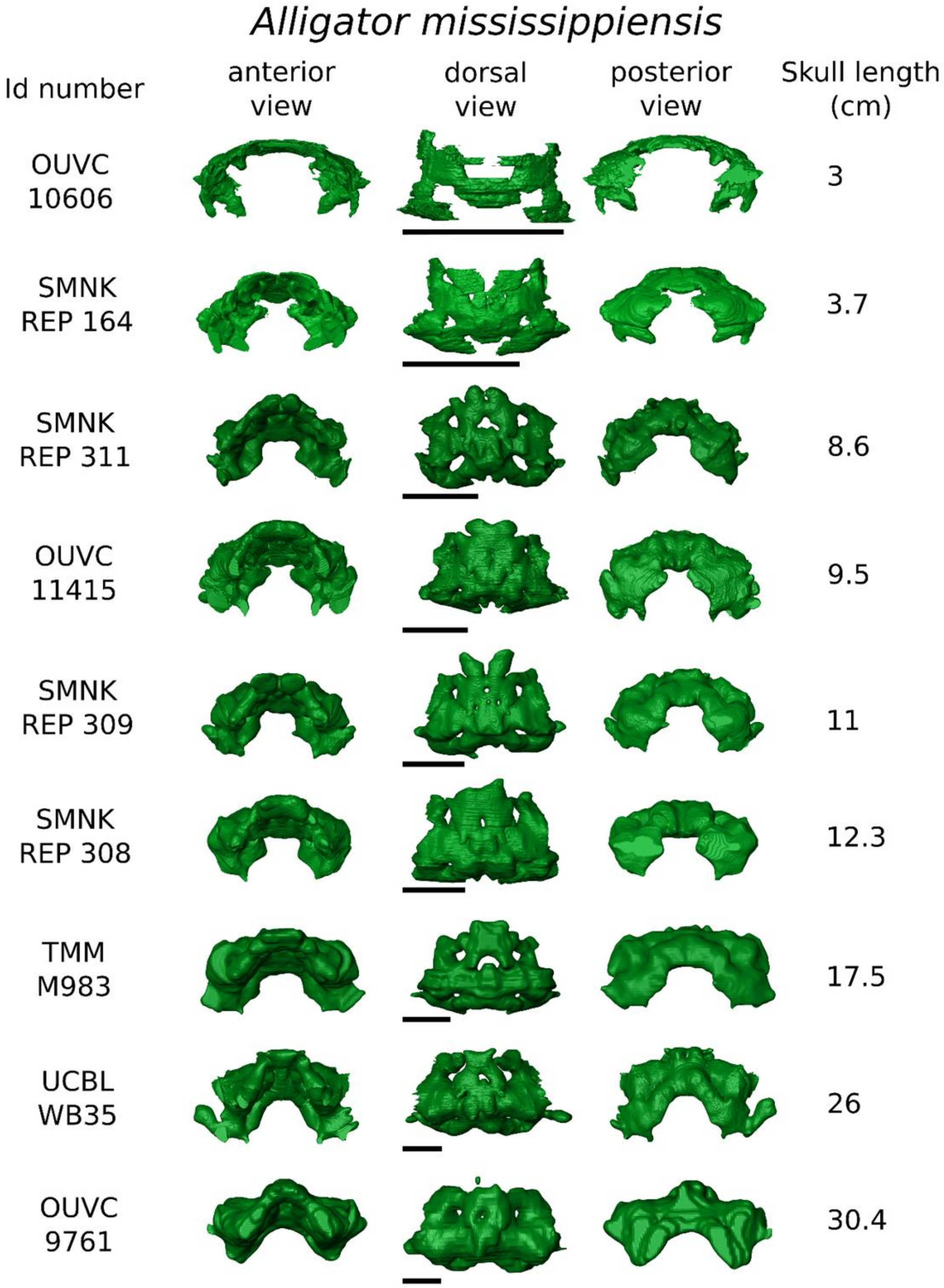

Fig. S1. 3D renderings of the intertympanic sinus system of all studied specimen of *Alligator mississipiensis*. Scale bars = 1 cm.

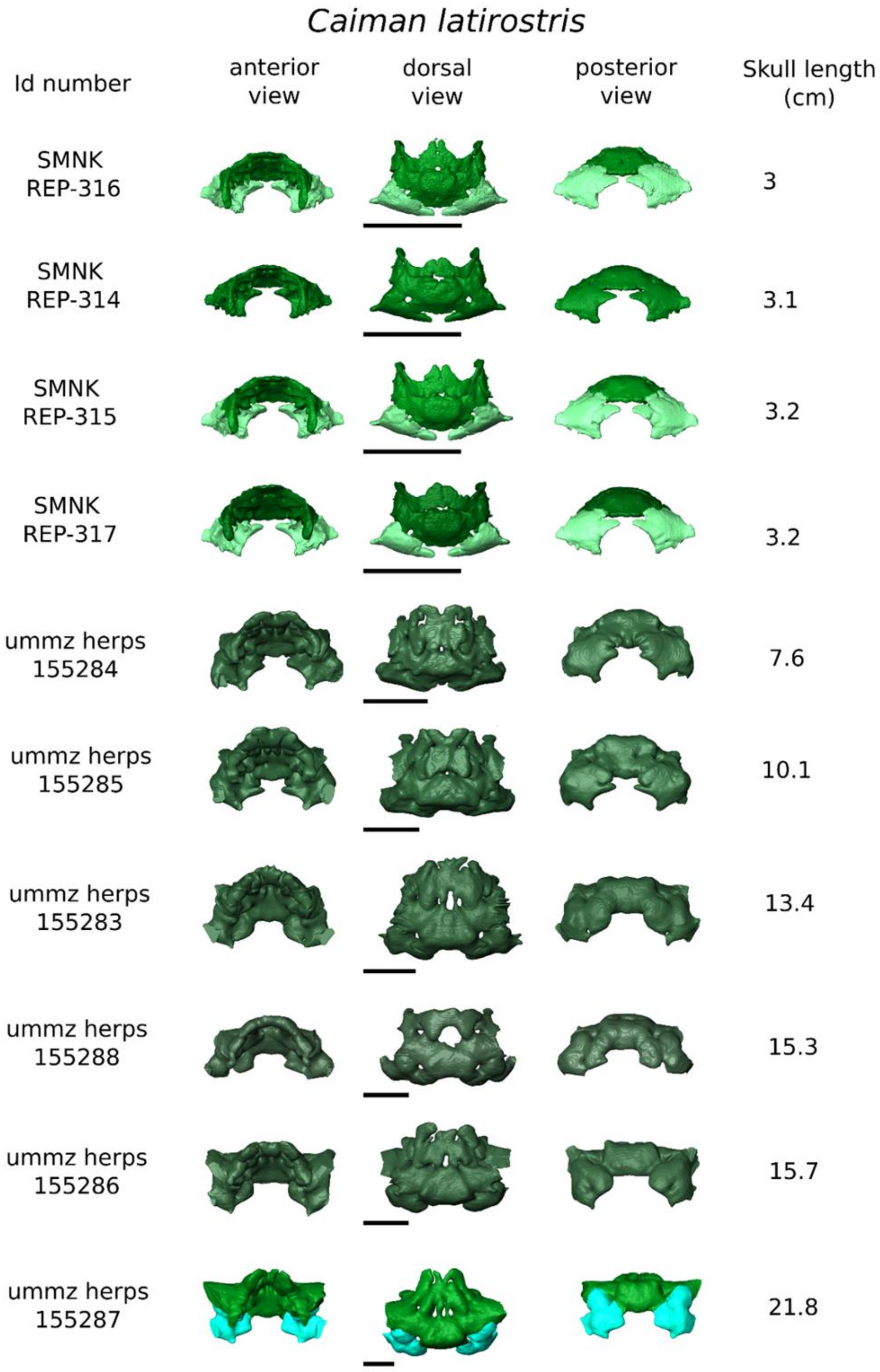

Fig. S2. 3D renderings of the intertympanic sinus system of all studied specimen of *Caiman latirostris*. Light green or blue colours indicate that the otoccipital recess could be segmented separately. Scale bars = 1 cm.

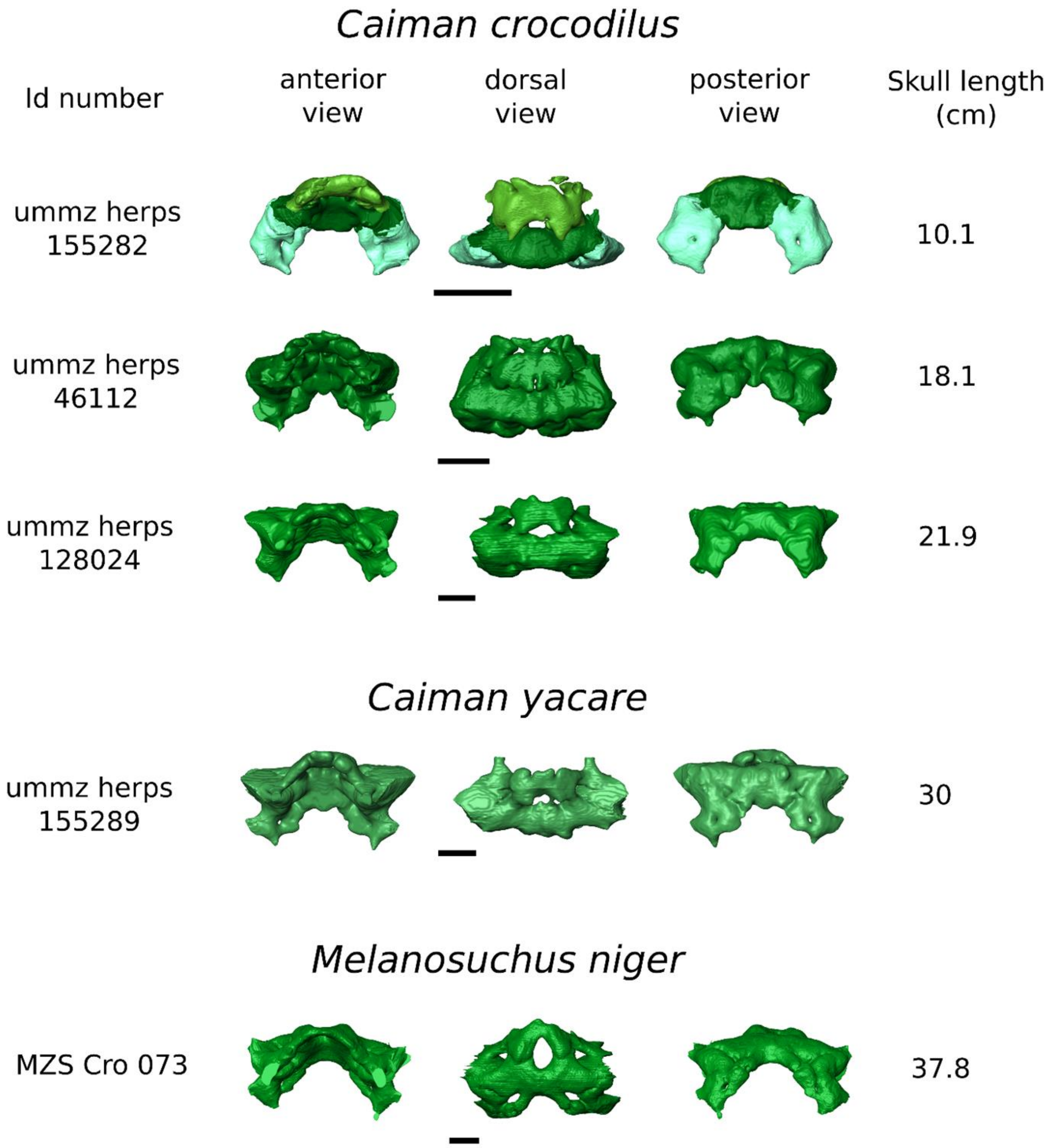

Fig. S3. 3D renderings of the intertympanic sinus system of all studied specimen of *Caiman crocodilus*, *Caiman yacare*, and *Melanosuchus niger*. Light green or blue colours indicate that the otoccipital recess and parietal recess could be segmented separately. Scale bars = 1 cm.

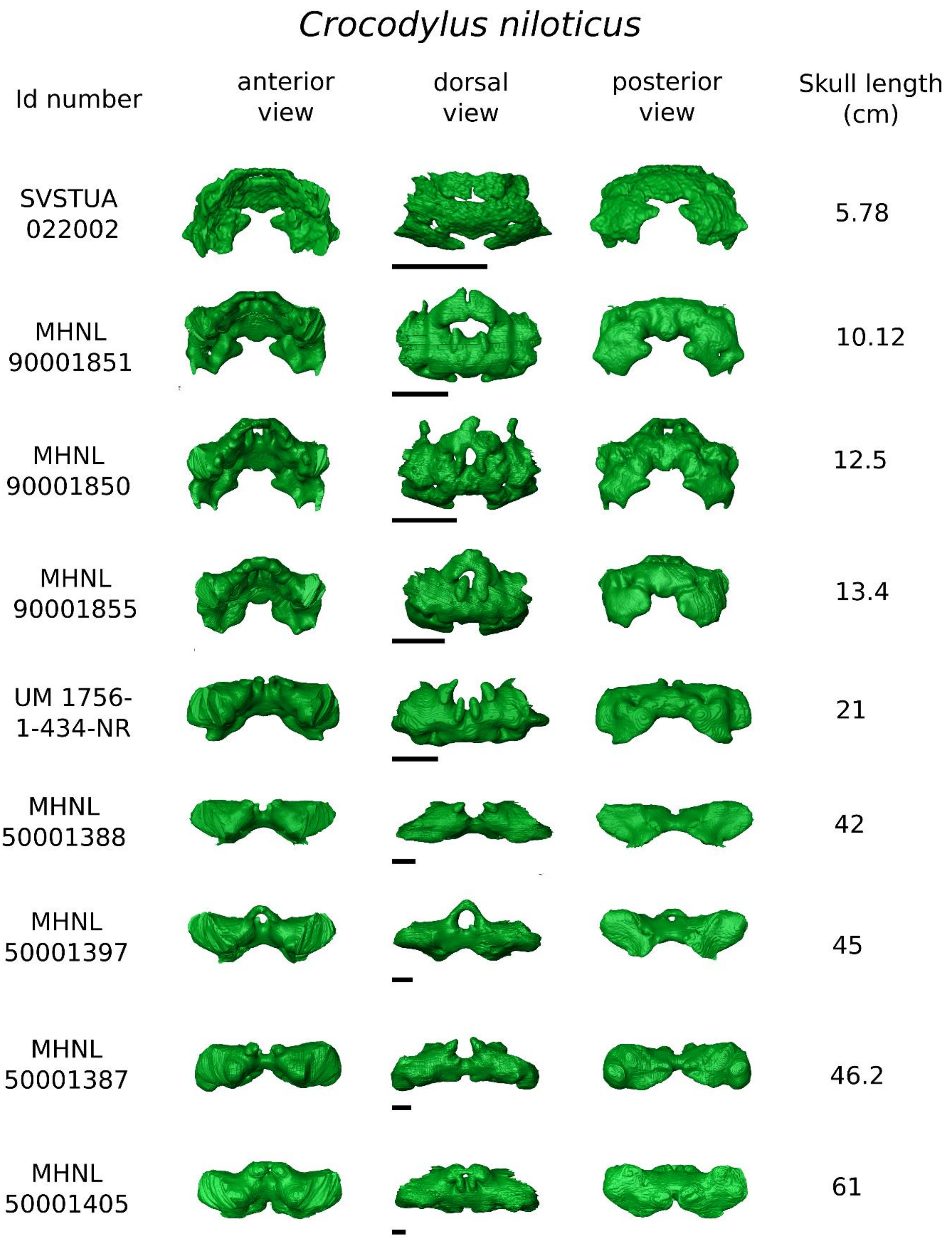

Fig. S4. 3D renderings of the intertympanic sinus system of all studied specimen of *Crocodylus niloticus*. Scale bars = 1 cm.

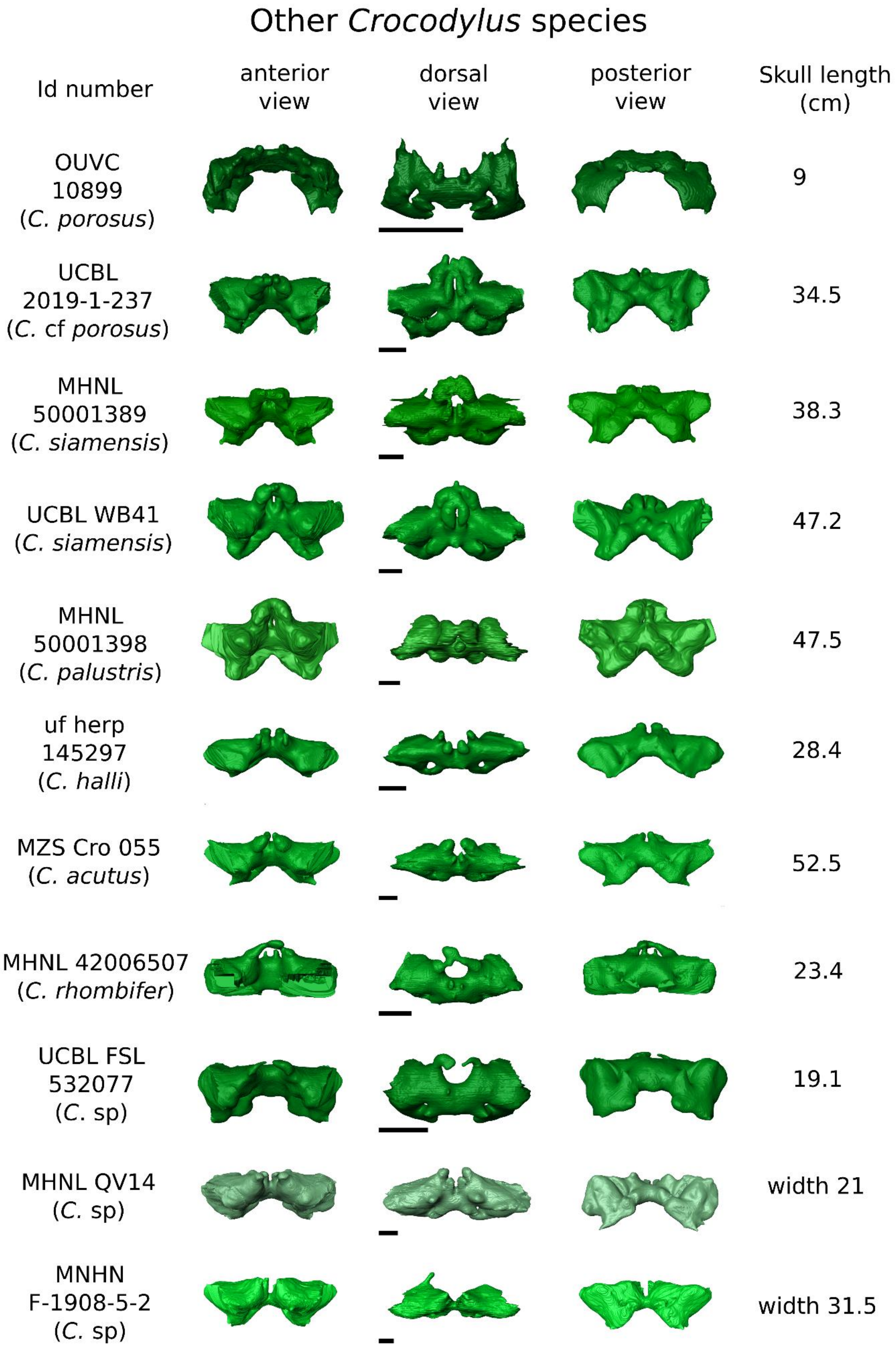

Fig. S5. 3D renderings of the intertympanic sinus system of studied specimen of *Crocodylus* species apart from *C. niloticus.* Scale bars = 1 cm.

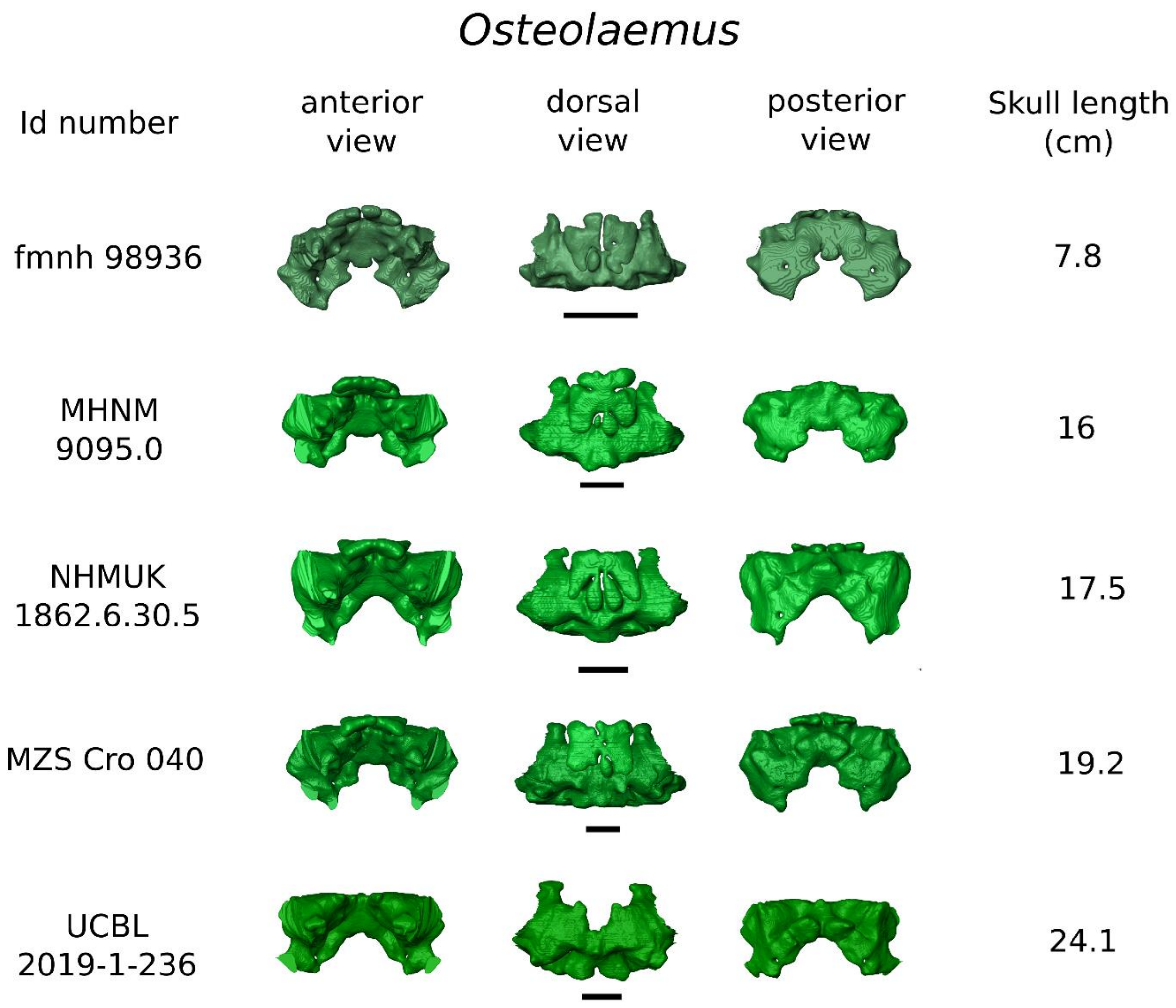

Fig. S6. 3D renderings of the intertympanic sinus system of all studied specimen of *Osteolaemus tetraspis*. Scale bars = 1 cm.

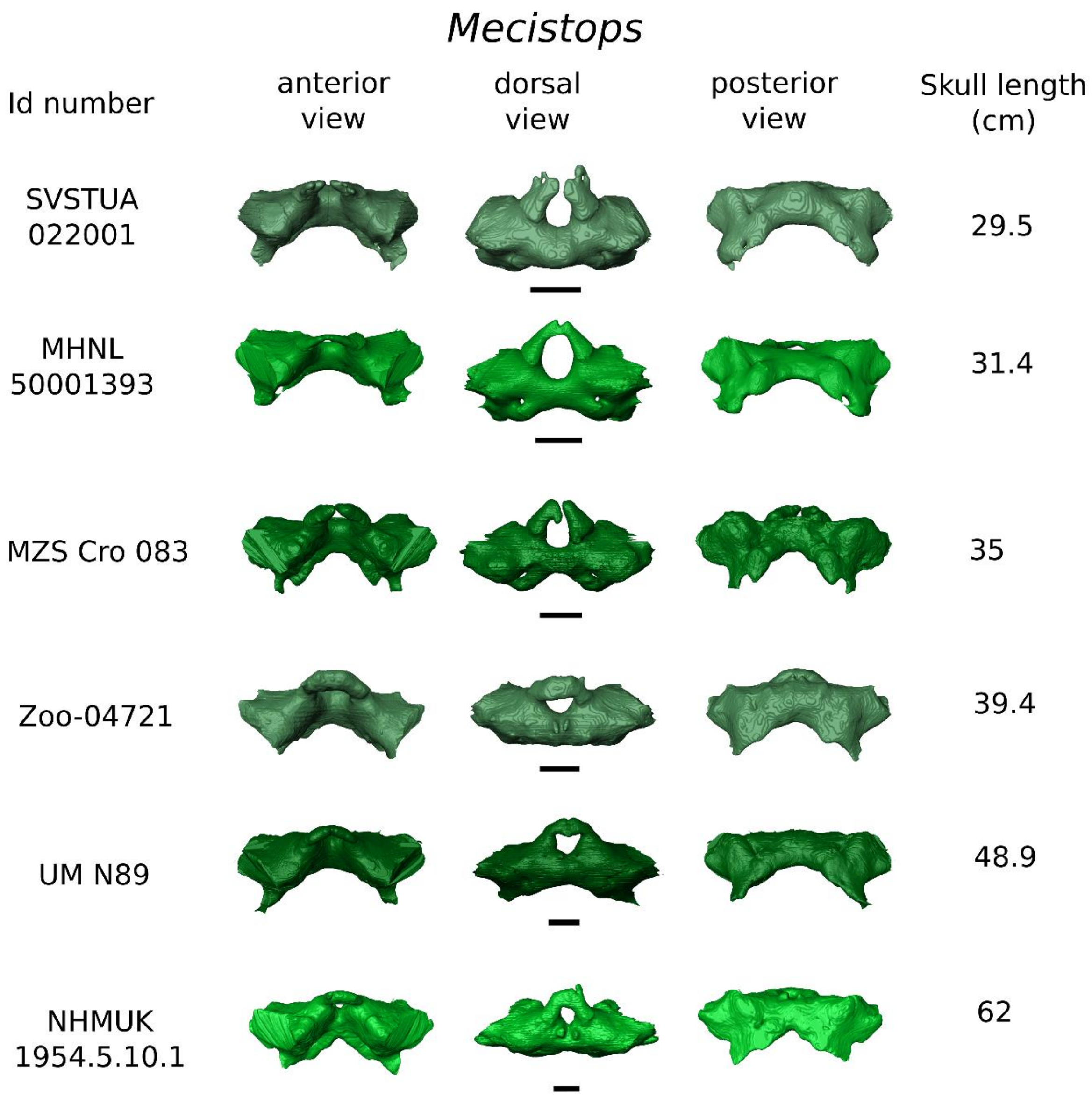

Fig. S7. 3D renderings of the intertympanic sinus system of all studied specimen of *Mecistops* spp. Scale bars = 1 cm.

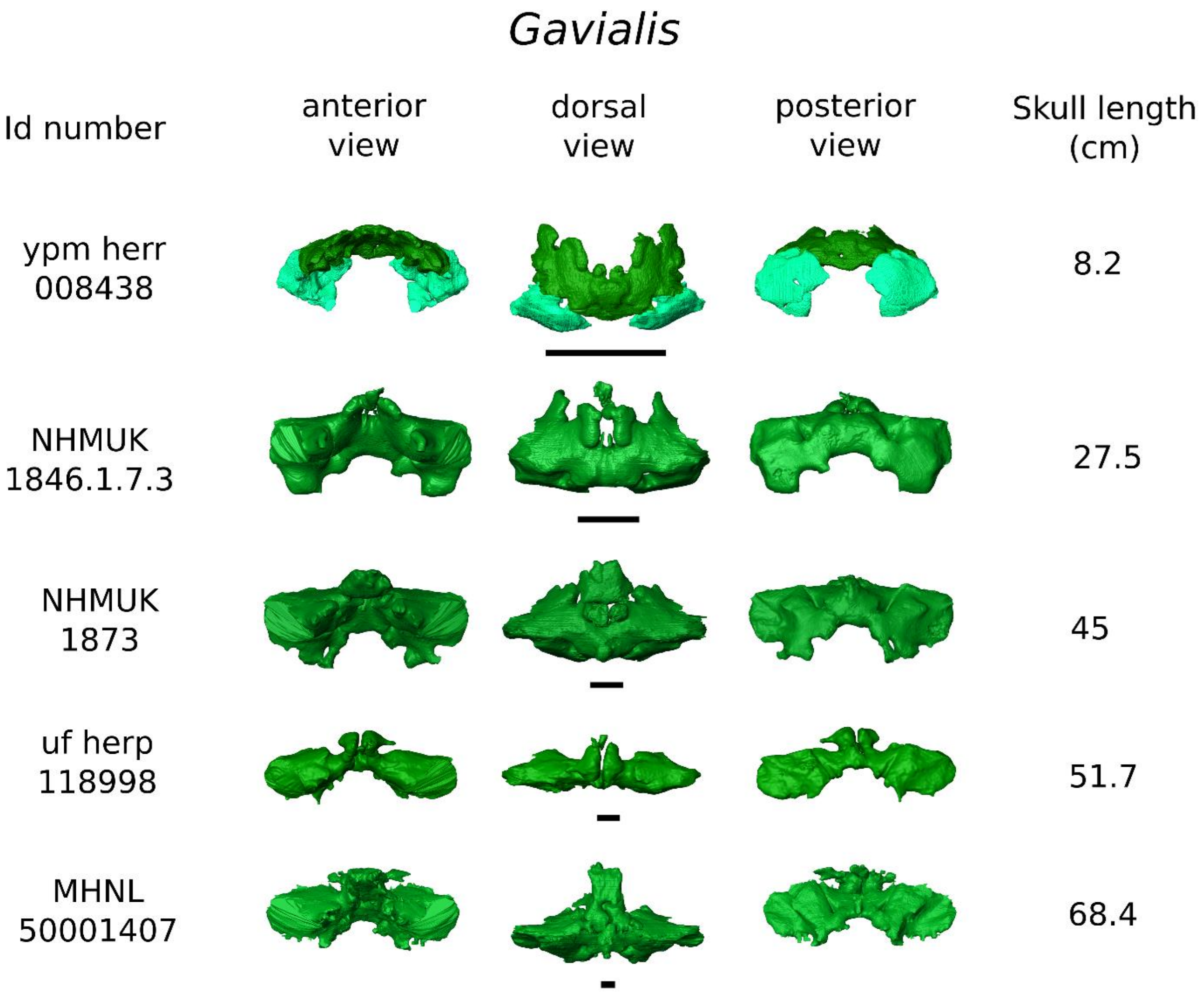

Fig. S8. 3D renderings of the intertympanic sinus system of all studied specimen of *Gavialis gangeticus*. Light green colour indicates that the otoccipital recess could be segmented separately. Scale bars = 1 cm.

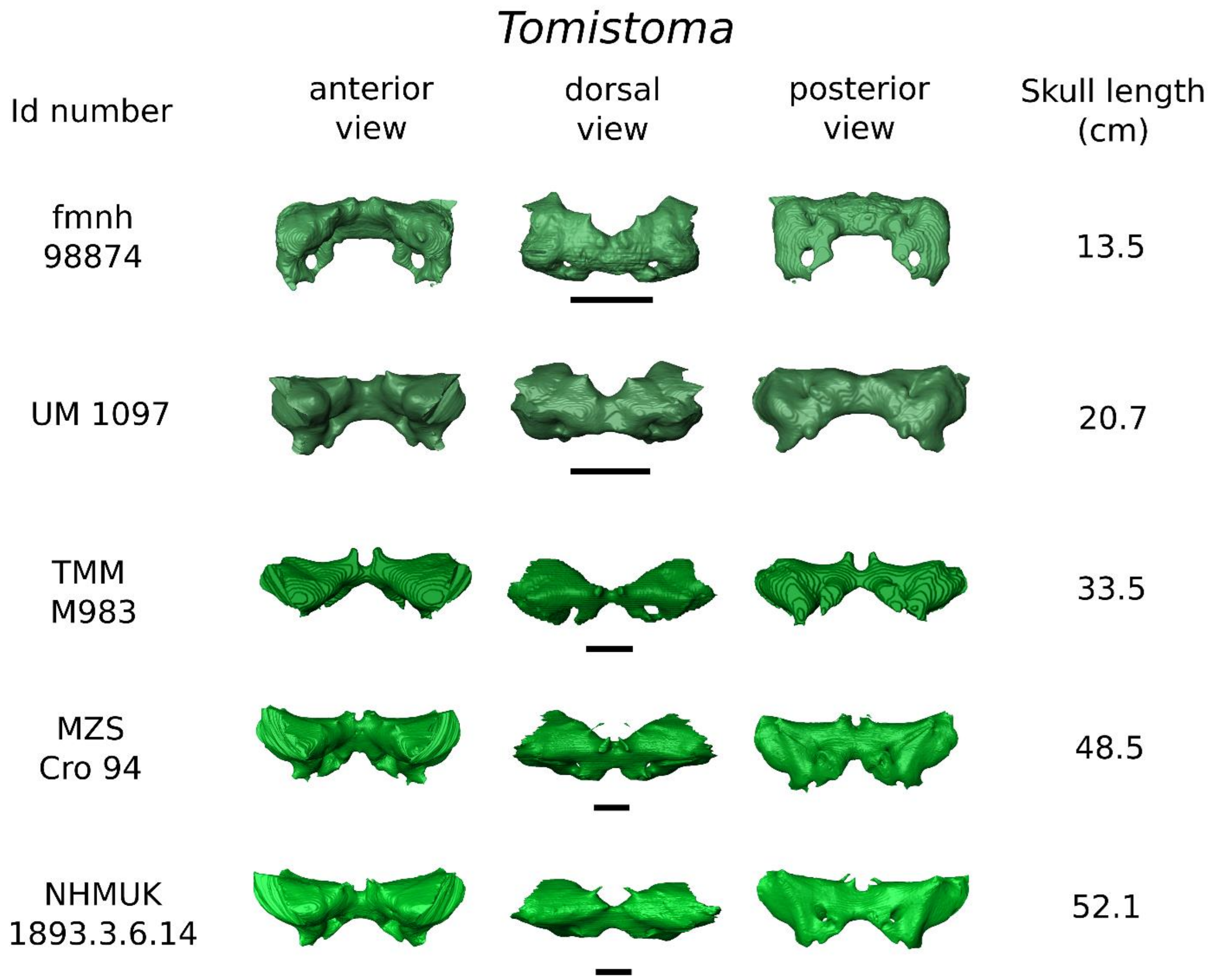

Fig. S9. 3D renderings of the intertympanic sinus system of all studied specimen of *Tomistoma schlegelii*. Scale bars = 1 cm.

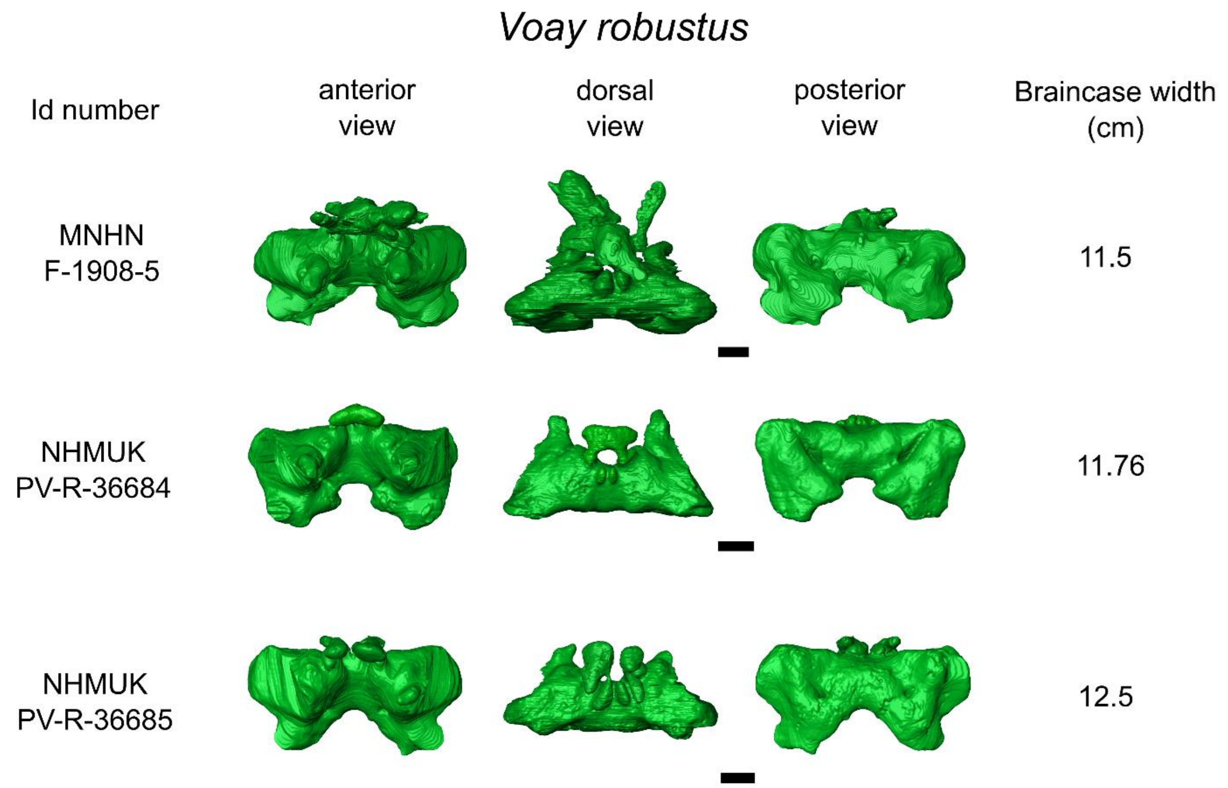

Fig. S10. 3D renderings of the intertympanic sinus system of all studied specimen of *Voay robustus*. Scale bars = 1 cm.

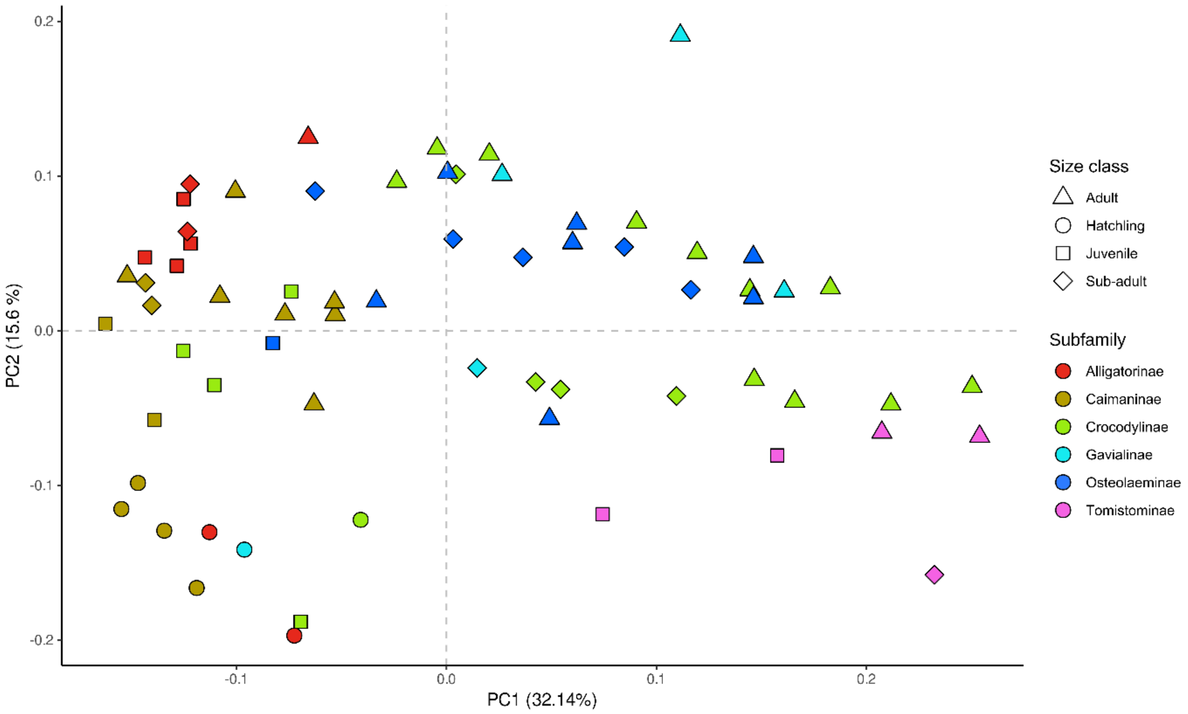

Fig. S11. Ontogenetic morphospace of Crocodylia with subfamilies represented.

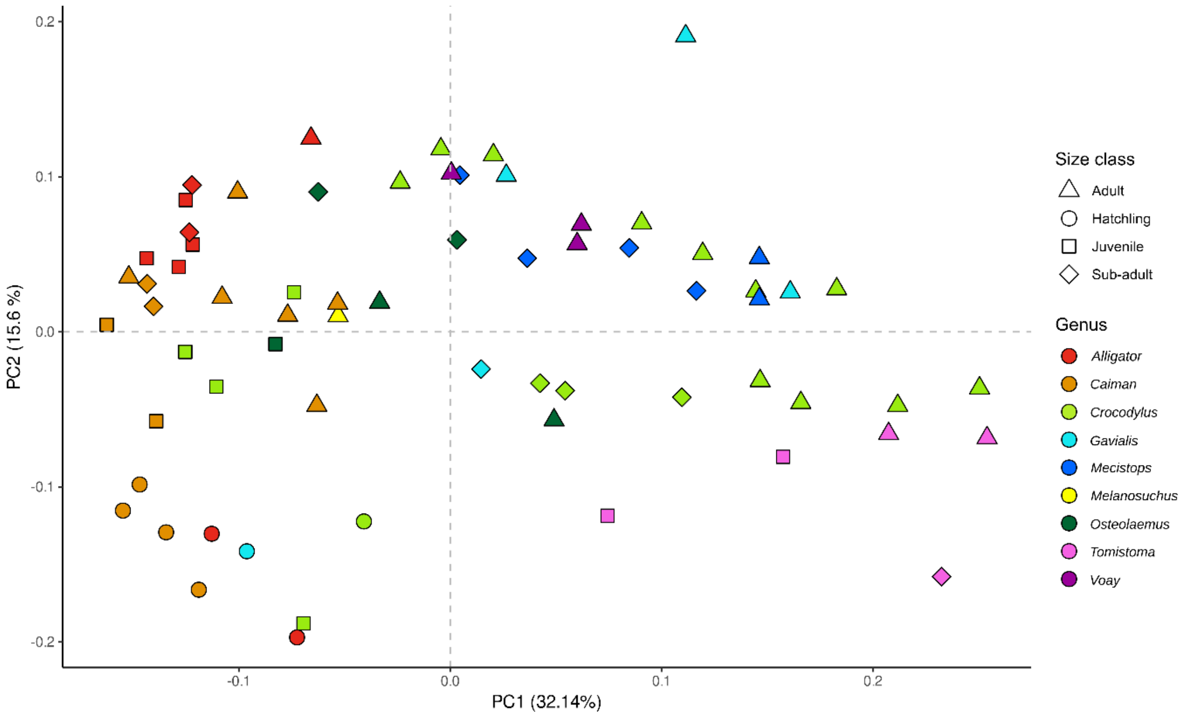

Fig. S12. Ontogenetic morphospace of Crocodylia with genera represented.

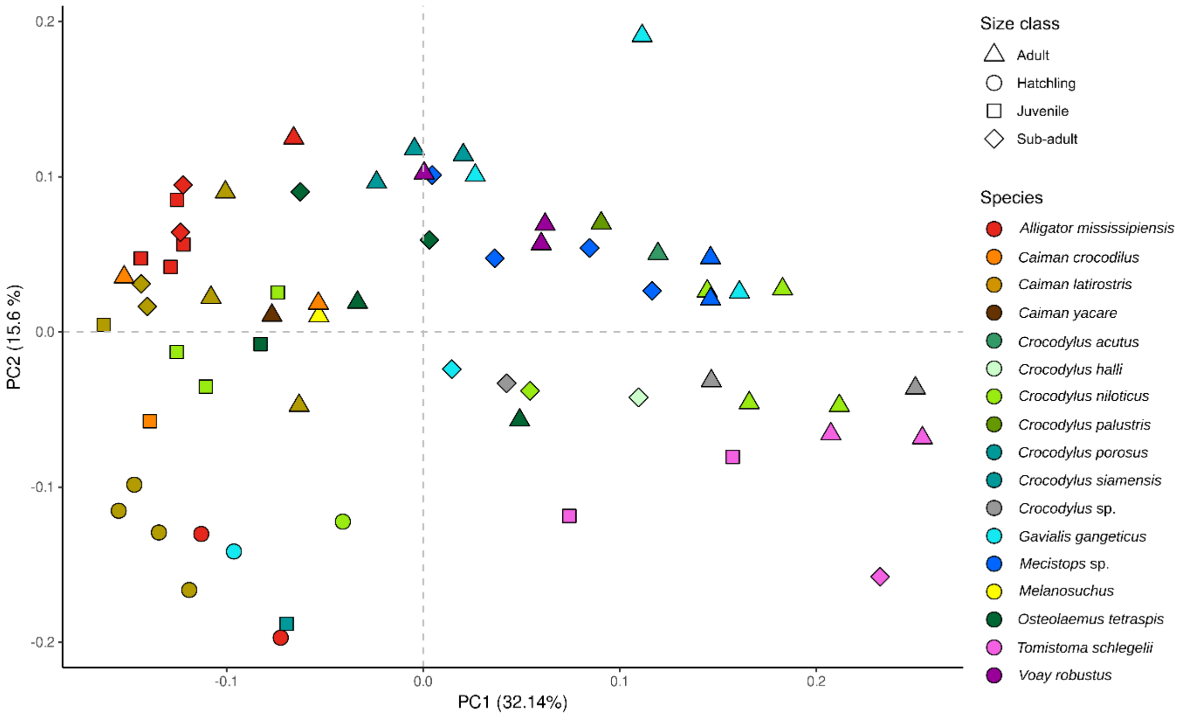

Fig. S13. Ontogenetic morphospace of Crocodylia with species represented.

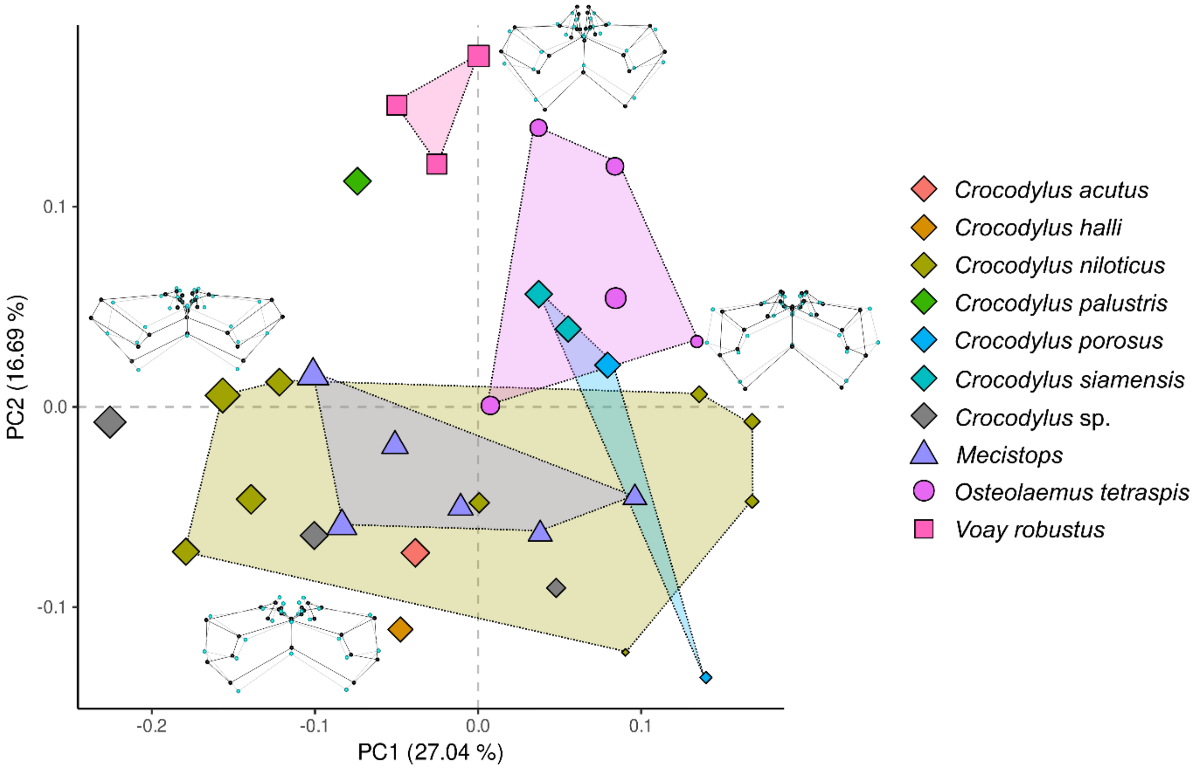

Fig. S14. Morphospace of the first two principal components of the Crocodylidae-only PCA.
Extreme shapes for each PC axis are given with wireframes in dorsal view (average shape in cyan).

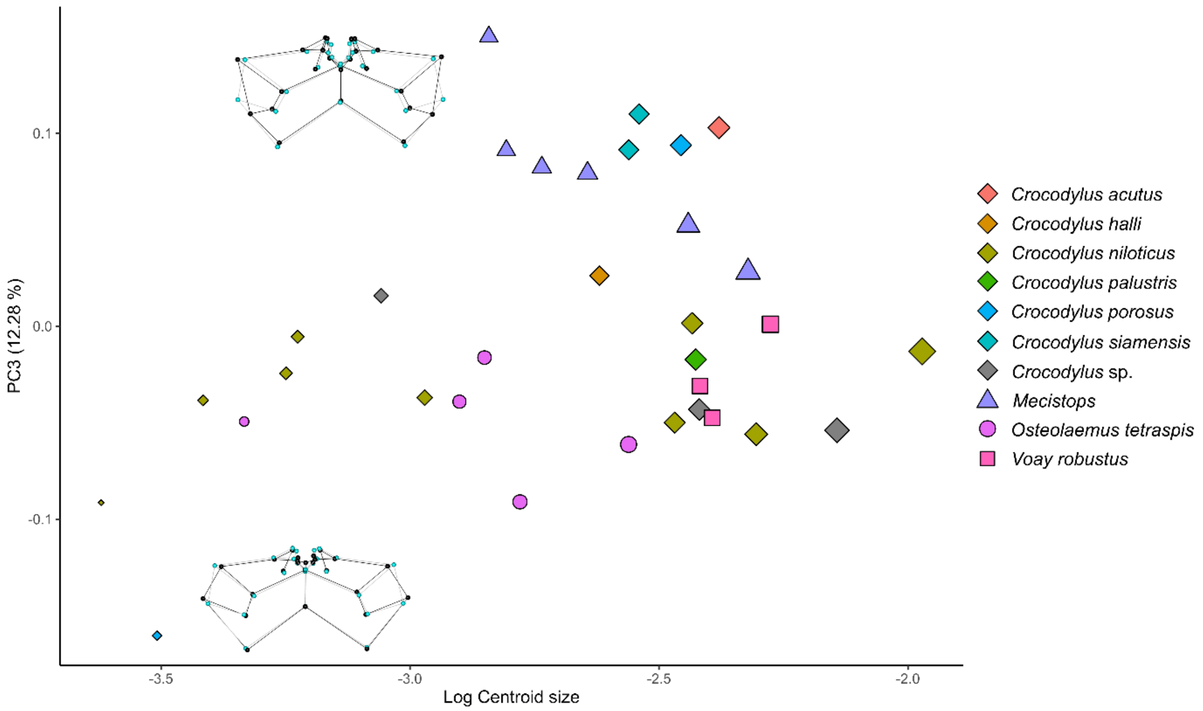

Fig. S15. PC3 of the Crocodylidae-only PCA versus Log Centroid Size of specimens. Extreme shapes for each PC axis are given with wireframes in dorsal view (average shape in cyan).

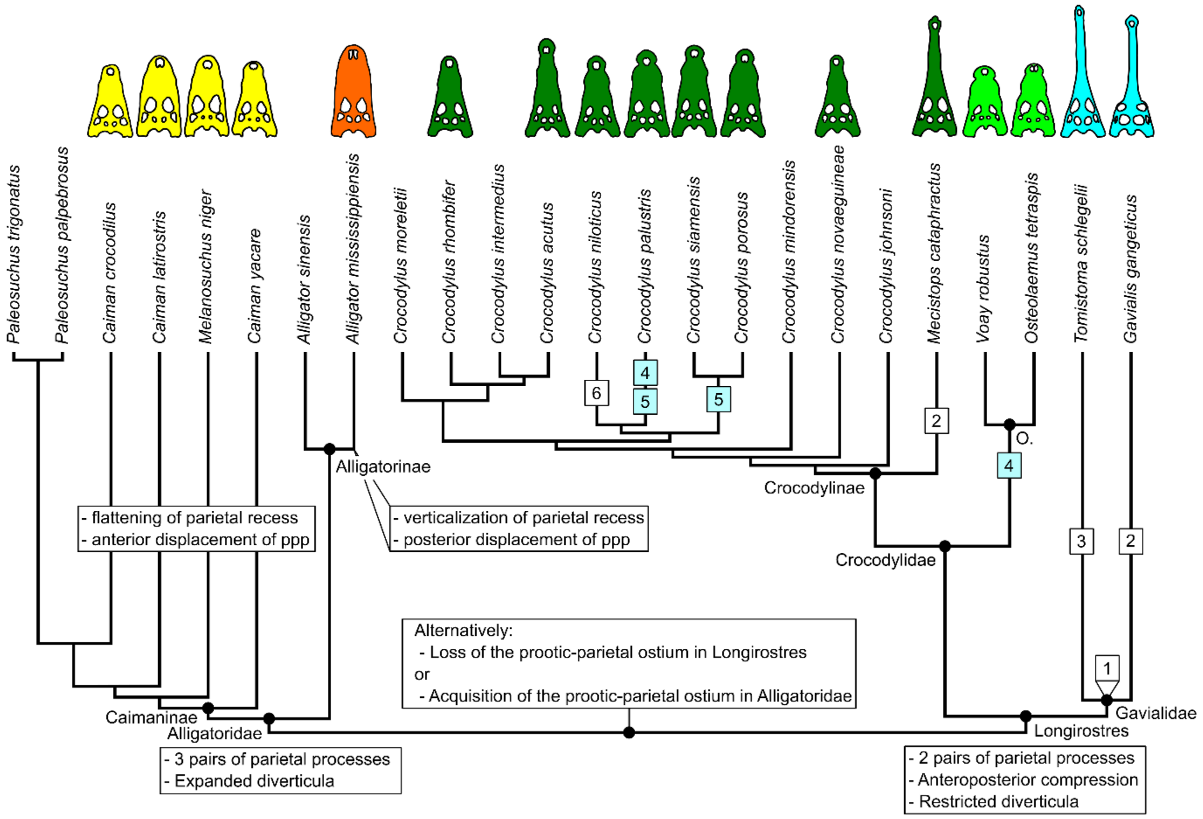

Fig. S16. Morphological phylogenetic tree of extant Crocodylia, including *Voay robustus*, associated with skull outlines of each studied species. Tree was constructed based on the topology of Rio & Mannion (*7*). O, Osteolaeminae; ppp, posteromedial parietal processes. (1) One-step post-hatching developmental sequence and reduction of otoccipital recess; (2) Reduction of the posteromedial parietal processes; (3) reduction of the anterolateral parietal processes and absence of parietal recess; (4) Expanded otoccipital and parietal recesses; (5) Vertical development of parietal recess; (6) reduction of parietal recess and reduction of otoccipital recess. Characters highlighted in light blue corresponds to conflicts between molecular (Figure 13) and morphological topologies.

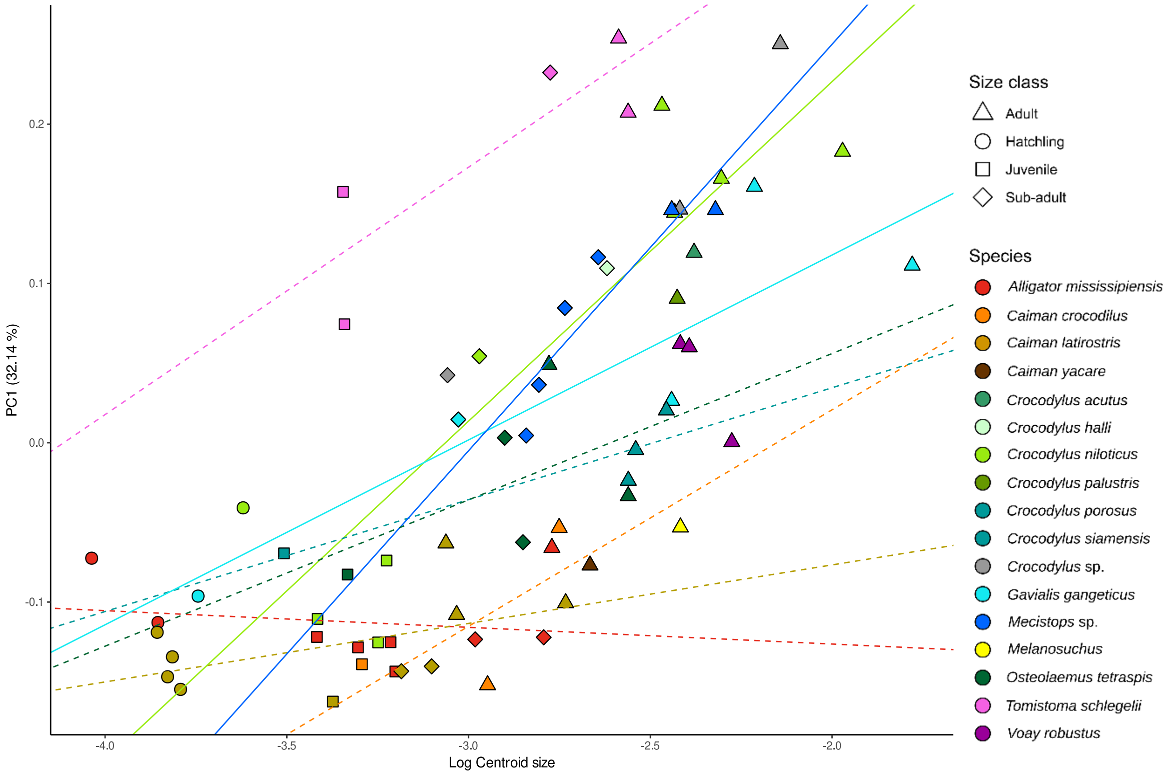

Fig. S17. PC1 scores of the total ontogeny morphospace against Log Centroid Size. Lines show linear regressions of the species listed in fig. S19. Dashed lines indicate the linear model that are not significant. Coefficients and regression parameters can be found in Supp. Table S5.

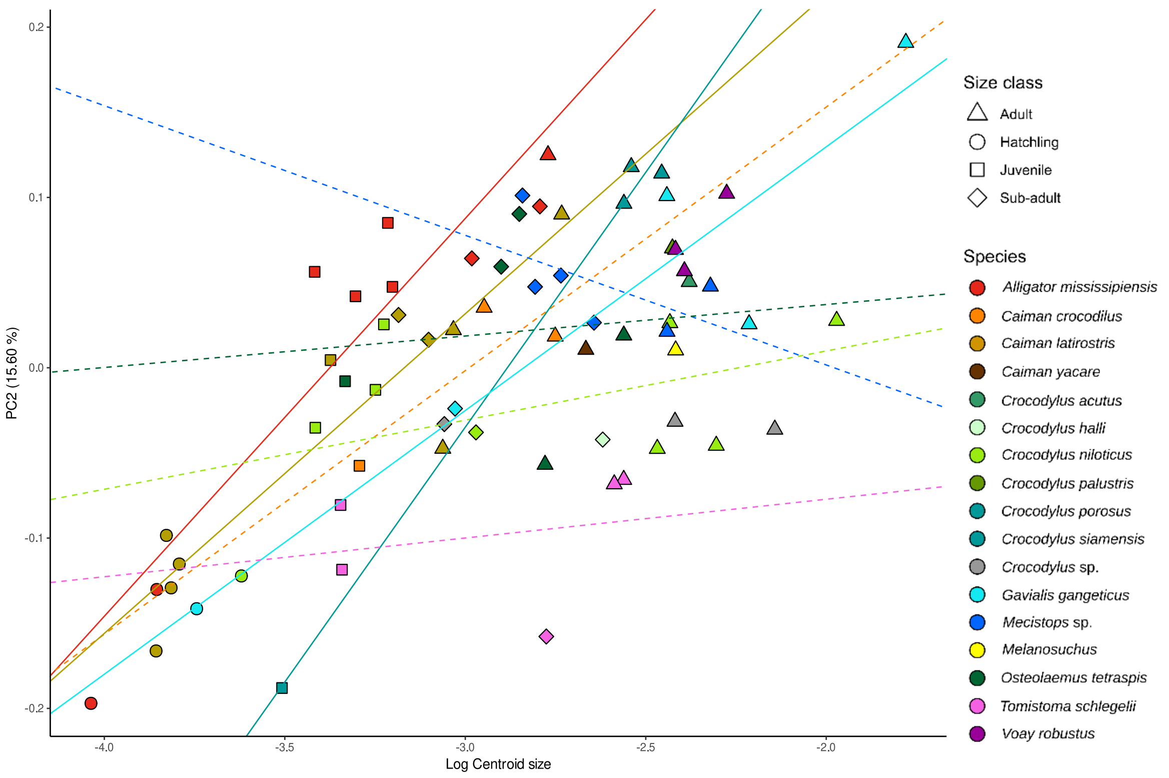

Fig. S18. PC2 scores of the total ontogeny morphospace against Log Centroid Size. Lines show linear regressions of the species listed in fig. S19. Dashed lines indicate the linear model that are not significant. Coefficients and regression parameters can be found in Supp. Table S5.

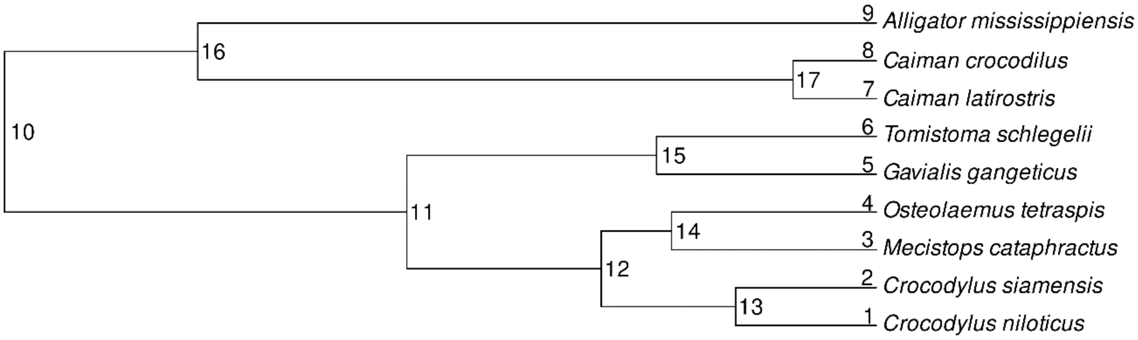

Fig. S19. Molecular tree topology used for ancestral state estimation of ontogenetic trajectory coefficients. Obtained from Timetree 5 (Kumar *et al*. 2022).

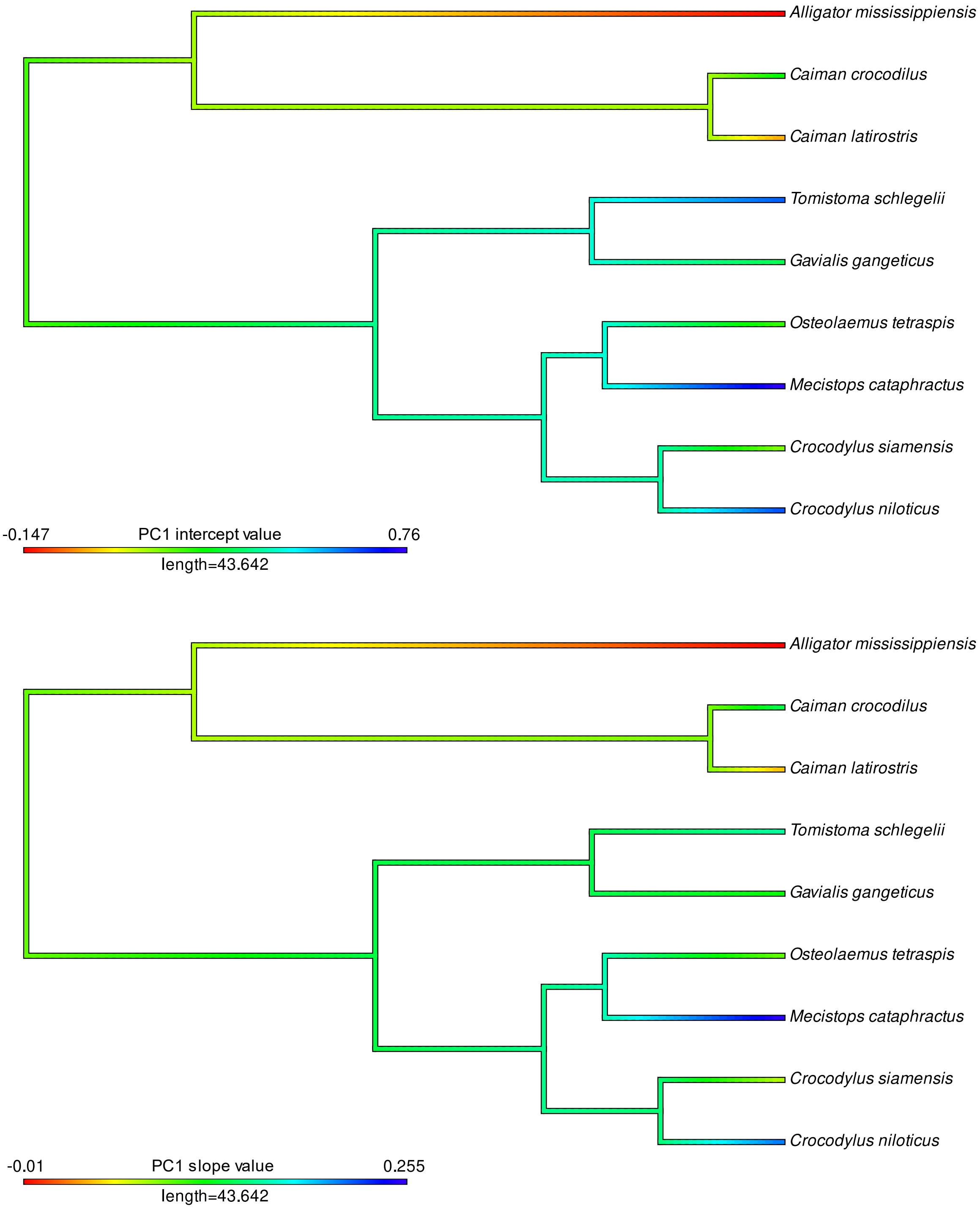

Fig. S20. Ancestral state reconstruction of PC1 ontogenetic trajectories mapped on the molecular topology.

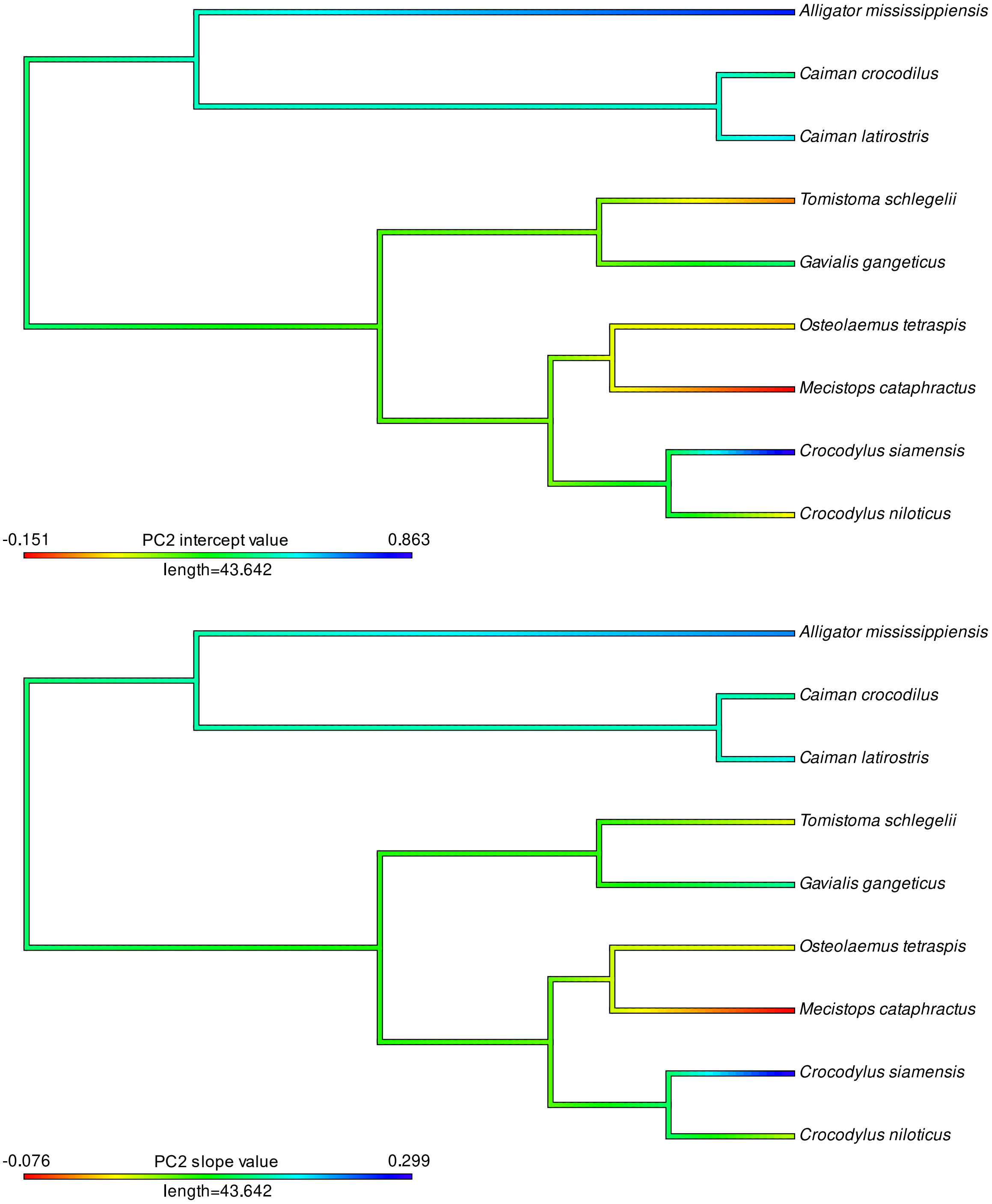

Fig. S21. Ancestral state reconstruction of PC2 ontogenetic trajectories mapped on the molecular topology.

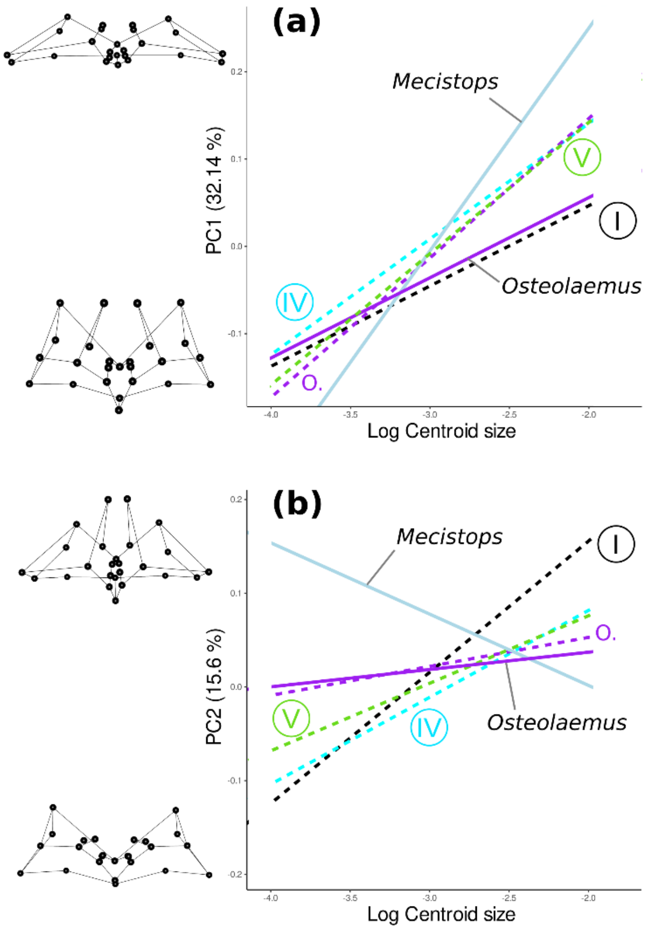

Fig. S22. Reconstructed PC1 and PC2 ontogenetic trajectories of Osteolaeminae with estimated ontogenetic trends at ancestral nodes. (a) PC1 ontogenies; (b) PC2 ontogenies. Dashed lines are hypothetical ancestral ontogenetic trajectories (roman numerals correspond to Fig. 13 in the main text). Extreme shapes for each PC axis are given with wireframes in dorsal view.
