# Supplementary material for: Ontogenetic variability of the intertympanic sinus distinguishes lineages within Crocodylia
Source: J Anat. 2023 Jan 29;242(6):1096–123. doi: 10.1111/joa.13830 (PMC10184552; doi:10.1111/joa.13830)
Supplement: Supplementary file 14 — Table S1. Table S2. Table S3. Table S4. Table S5. Table S6. Table S7. [file JOA-242-1096-s012.docx]

**Ontogenetic variability of the intertympanic sinus distinguishes lineages within Crocodylia**

Gwendal PERRICHON, Lionel HAUTIER, Yohan POCHAT-COTTILLOUX, Irena RASELLI, Céline SALAVIALE, Benjamin DAILH, Nicolas RINDER, Vincent FERNANDEZ, Jérôme ADRIEN, Joël LACHAMBRE, Jeremy E. MARTIN*

**Supplementary Tables**

Table S1. Total skull length classes for each genera used to infer ontogenetic stages of all specimens studied.

Table S2. Results of the Procrustes ANOVA on total shape and homogeneity of slopes test.

Table S3. Results of the Procrustes ANOVA on PC scores in the total ontogeny morphospace.

Table S4: Results of the Procrustes ANOVA in the restricted sub-clade analyses

Table S5: Results of the linear regressions of principal components versus size in the total ontogeny analysis.

Table S6: Ancestral state estimation of ontogenetic trajectory coefficients on PC1 and PC2.

Table S7: Results of the linear regression of Principal components versus size in the restricted sub-clade analyses.

1. Supplementary Table S1: Total skull length classes for each genera used to infer ontogenetic stages of all specimens studied.

| **Genus** | **Hatchling** | **Juvenile** | **Sub-adult** | **Adult** |
| --- | --- | --- | --- | --- |
| *Alligator* | < 5 | [5-15[ | [15-30[ | ≥ 30 |
| *Caiman* | < 5 | [5-10[ | [10-15[ | ≥ 15 |
| *Melanosuchus* | < 5 | [5-15[ | [15-30[ | ≥ 30 |
| *Osteolaemus* | < 5 | [5-10[ | [10-15[ | ≥ 15 |
| *Crocodylus* | < 6 | [5-15[ | [15-30[ | ≥ 30 |
| *Mecistops* | < 8 | [8-25[ | [25-40[ | ≥ 40 |
| *Gavialis* | < 10 | [10-25[ | [25-40[ | ≥ 40 |
| *Tomistoma* | < 10 | [10-25[ | [25-40[ | ≥ 40 |

1. Supplementary Table S2: Results of the Procrustes ANOVA on total shape and homogeneity of slopes test.

Crocodylia Procrustes shape coordinates vs Size (vector) and Species (factor)

|  | Df | SS | MS | **R sq** | F | Z | **Pr(>F)** |
| --- | --- | --- | --- | --- | --- | --- | --- |
| Log(CS) | 1 | 0.63184 | 0.63184 | **0.21111** | 37.1698 | 5.4572 | **0.001** |
| Species | 16 | 1.26924 | 0.07933 | **0.42407** | 4.6667 | 9.4031 | **0.001** |
| Log(CS):Species | 10 | 0.42894 | 0.04289 | **0.14331** | 2.5233 | 5.9485 | **0.001** |
| Residuals | 39 | 0.66295 | 0.01700 | 0.22150 |  |  |  |
| Total | 66 | 2.99296 |  |  |  |  |  |

Homogeneity of slopes test

|  | ResDf | Df | RSS | SS | MS | **R sq** | F | Z | **P** |
| --- | --- | --- | --- | --- | --- | --- | --- | --- | --- |
| Common allometry | 49 | 1 | 1.09189 |  |  | **0.00000** |  |  |  |
| Unique allometry | 39 | 10 | 0.66295 | 0.42894 | 0.042894 | **0.14331** | 2.5233 | 5.9485 | **0.001** |
| Total | 66 |  | 2.99296 |  |  |  |  |  |  |

Crocodylia Procrustes shape coordinates vs Size (vector) and Snout shape (factor: Brevirostrine / Longirostrine / Mesorostrine)

|  | Df | SS | MS | **R sq** | F | Z | **Pr(>F)** |
| --- | --- | --- | --- | --- | --- | --- | --- |
| Log(CS) | 1 | 0.63184 | 0.63184 | **0.21111** | 21.5995 | 4.9642 | **0.001** |
| Snout shape | 2 | 0.43276 | 0.21638 | **0.14459** | 7.3970 | 4.7112 | **0.001** |
| Log(CS):Snout shape | 2 | 0.14396 | 0.07198 | **0.04810** | 2.4607 | 3.0584 | **0.001** |
| Residuals | 61 | 1.78440 | 0.02925 | 0.59620 |  |  |  |
| Total | 66 | 2.99296 |  |  |  |  |  |

Crocodylia Procrustes shape coordinates vs Ecology (factor: Semi-aquatic / Sub-aquatic / Sub-terrestrial)

|  | Df | SS | MS | **R sq** | F | Z | **Pr(>F)** |
| --- | --- | --- | --- | --- | --- | --- | --- |
| Ecology | 2 | 0.22297 | 0.111484 | **0.0745** | 2.5758 | 2.5791 | **0.003** |
| Residuals | 64 | 2.76999 | 0.043281 | 0.9255 |  |  |  |
| Total | 66 | 2.99296 |  |  |  |  |  |

1. Supplementary Table S3: Results of the Procrustes ANOVA on PC scores in the total ontogeny morphospace.

Crocodylia PC1 (32.14%) coordinates vs Size (vector) and Species (factor)

|  | Df | SS | MS | **R sq** | F | Z | **Pr(>F)** |
| --- | --- | --- | --- | --- | --- | --- | --- |
| Log(CS) | 1 | 0.44110 | 0.44110 | **0.45853** | 255.182 | 6.9726 | **0.001** |
| Species | 16 | 0.37919 | 0.02370 | **0.39417** | 13.710 | 7.7020 | **0.001** |
| Log(CS):Species | 10 | 0.07429 | 0.00743 | **0.07723** | 4.298 | 3.3806 | **0.001** |
| Residuals | 39 | 0.06741 | 0.00173 | 0.07008 |  |  |  |
| Total | 66 | 0.96200 |  |  |  |  |  |

Crocodylia PC2 (15.6%) coordinates vs Size (vector) and Species (factor)

|  | Df | SS | MS | **R sq** | F | Z | **Pr(>F)** |
| --- | --- | --- | --- | --- | --- | --- | --- |
| Log(CS) | 1 | 0.18032 | 0.180323 | **0.38619** | 104.8846 | 5.7032 | **0.001** |
| Species | 16 | 0.12790 | 0.007993 | **0.27391** | 4.6494 | 4.1106 | **0.001** |
| Log(CS):Species | 10 | 0.09166 | 0.009166 | **0.19631** | 5.3315 | 4.1364 | **0.001** |
| Residuals | 39 | 0.06705 | 0.001719 | 0.14360 |  |  |  |
| Total | 66 | 0.46693 |  |  |  |  |  |

1. Supplementary Table S4: Results of the Procrustes ANOVA in the restricted sub-clade analyses.

**Alligatoridae-only PCA**

Alligatoridae Procrustes shape coordinates vs Size (vector) and Species (factor)

|  | Df | SS | MS | **R sq** | F | Z | **Pr(>F)** |
| --- | --- | --- | --- | --- | --- | --- | --- |
| Log(CS) | 1 | 0.17422 | 0.174223 | **0.26454** | 12.9338 | 4.1319 | **0.001** |
| Species | 4 | 0.22105 | 0.055264 | **0.33565** | 4.1026 | 4.9830 | **0.001** |
| Log(CS):Species | 2 | 0.04778 | 0.023889 | **0.07255** | 1.7735 | 1.7765 | **0.039** |
| Residuals | 16 | 0.21553 | 0.013470 | 0.32726 |  |  |  |
| Total | 23 | 0.65858 |  |  |  |  |  |

Alligatoridae PC1 (35.02%) coordinates vs Size (vector) and Species (factor)

|  | Df | SS | MS | **R sq** | F | Z | **Pr(>F)** |
| --- | --- | --- | --- | --- | --- | --- | --- |
| Log(CS) | 1 | 0.149464 | 0.149464 | **0.64808** | 107.3433 | 4.9005 | **0.001** |
| Species | 4 | 0.058418 | 0.014604 | **0.25330** | 10.4887 | 3.3585 | **0.001** |
| Log(CS):Species | 2 | 0.000465 | 0.000232 | 0.00202 | 0.1669 | -0.9090 | 0.810 |
| Residuals | 16 | 0.022278 | 0.001392 | 0.09660 |  |  |  |
| Total | 23 | 0.230625 |  |  |  |  |  |

Alligatoridae PC2 (19.38%) coordinates vs Size (vector) and Species (factor)

|  | Df | SS | MS | **R sq** | F | Z | **Pr(>F)** |
| --- | --- | --- | --- | --- | --- | --- | --- |
| Log(CS) | 1 | 0.016266 | 0.0162657 | **0.12742** | 48.872 | 4.3041 | **0.001** |
| Species | 4 | 0.104087 | 0.0260217 | **0.81539** | 78.184 | 9.2699 | **0.001** |
| Log(CS):Species | 2 | 0.001975 | 0.0009875 | 0.01547 | 2.967 | 1.3661 | 0.087 |
| Residuals | 16 | 0.005325 | 0.0003328 | 0.04172 |  |  |  |
| Total | 23 | 0.127653 |  |  |  |  |  |

**Crocodylidae-only PCA**

Crocodylidae Procrustes shape coordinates vs Size (vector) and Species (factor)

|  | Df | SS | MS | **R sq** | F | Z | **Pr(>F)** |
| --- | --- | --- | --- | --- | --- | --- | --- |
| Log(CS) | 1 | 0.26828 | 0.268276 | **0.21103** | 14.5393 | 6.0044 | **0.001** |
| Species | 9 | 0.51818 | 0.057576 | **0.40761** | 3.1203 | 6.3212 | **0.001** |
| Log(CS):Species | 5 | 0.17114 | 0.034227 | **0.13462** | 1.8550 | 2.5286 | **0.005** |
| Residuals | 17 | 0.31368 | 0.018452 | 0.24674 |  |  |  |
| Total | 32 | 1.27127 |  |  |  |  |  |

Crocodylidae PC1 (27.04%) coordinates vs Size (vector) and Species (factor)

|  | Df | SS | MS | **R sq** | F | Z | **Pr(>F)** |
| --- | --- | --- | --- | --- | --- | --- | --- |
| Log(CS) | 1 | 0.22412 | 0.224124 | **0.65192** | 91.2982 | 5.4508 | **0.001** |
| Species | 9 | 0.05201 | 0.005779 | 0.15128 | 2.3539 | 1.5145 | 0.067 |
| Log(CS):Species | 5 | 0.02593 | 0.005186 | 0.07542 | 2.1124 | 1.1850 | 0.116 |
| Residuals | 17 | 0.04173 | 0.002455 | 0.12139 |  |  |  |
| Total | 32 | 0.34379 |  |  |  |  |  |

Crocodylidae PC2 (16.69%) coordinates vs Size (vector) and Species (factor)

|  | Df | SS | MS | **R sq** | F | Z | **Pr(>F)** |
| --- | --- | --- | --- | --- | --- | --- | --- |
| Log(CS) | 1 | 0.025883 | 0.0258828 | **0.12202** | 14.1878 | 2.7113 | **0.003** |
| Species | 9 | 0.143953 | 0.0159948 | **0.67865** | 8.7676 | 4.4342 | **0.001** |
| Log(CS):Species | 5 | 0.011267 | 0.0022533 | 0.05312 | 1.2352 | 0.3304 | 0.371 |
| Residuals | 17 | 0.031013 | 0.0018243 | 0.14621 |  |  |  |
| Total | 32 | 0.212116 |  |  |  |  |  |

Crocodylidae PC3 (12.28%) coordinates vs Size (vector) and Species (factor)

|  | Df | SS | MS | **R sq** | F | Z | **Pr(>F)** |
| --- | --- | --- | --- | --- | --- | --- | --- |
| Log(CS) | 1 | 0.013827 | 0.0138274 | **0.08856** | 21.533 | 3.2915 | **0.010** |
| Species | 9 | 0.078874 | 0.0087638 | **0.50515** | 13.648 | 4.9353 | **0.001** |
| Log(CS):Species | 5 | 0.052522 | 0.0105044 | **0.33638** | 16.358 | 3.4970 | **0.001** |
| Residuals | 17 | 0.010917 | 0.0006421 | 0.06991 |  |  |  |
| Total | 32 | 0.156140 |  |  |  |  |  |

**Gavialidae-only PCA**

Gavialidae Procrustes shape coordinates vs Size (vector) and Species (factor)

|  | Df | SS | MS | **R sq** | F | Z | **Pr(>F)** |
| --- | --- | --- | --- | --- | --- | --- | --- |
| Log(CS) | 1 | 0.13414 | 0.134143 | **0.30791** | 6.0428 | 2.91821 | **0.001** |
| Species | 1 | 0.14092 | 0.140925 | **0.32347** | 6.3483 | 2.50976 | **0.003** |
| Log(CS):Species | 1 | 0.02740 | 0.027399 | 0.06289 | 1.2343 | 0.61304 | 0.267 |
| Residuals | 6 | 0.13319 | 0.022199 | 0.30573 |  |  |  |
| Total | 9 | 0.43566 |  |  |  |  |  |

Gavialidae PC1 (41.45%) vs Size (vector) and Species (factor)

|  | Df | SS | MS | **R sq** | F | Z | **Pr(>F)** |
| --- | --- | --- | --- | --- | --- | --- | --- |
| Log(CS) | 1 | 0.048800 | 0.048800 | **0.27024** | 15.3140 | 2.2655 | **0.011** |
| Species | 1 | 0.104103 | 0.104103 | **0.57649** | 32.6687 | 2.9016 | **0.005** |
| Log(CS):Species | 1 | 0.008558 | 0.008558 | 0.04739 | 2.6856 | 1.0690 | 0.149 |
| Residuals | 6 | 0.019120 | 0.003187 | 0.10588 |  |  |  |
| Total | 9 | 0.180580 |  |  |  |  |  |

Gavialidae PC2 (28.8%) vs Size (vector) and Species (factor)

|  | Df | SS | MS | **R sq** | F | Z | **Pr(>F)** |
| --- | --- | --- | --- | --- | --- | --- | --- |
| Log(CS) | 1 | 0.084904 | 0.084904 | **0.67670** | 55.0470 | 3.6126 | **0.001** |
| Species | 1 | 0.031041 | 0.031041 | **0.24740** | 20.1254 | 2.4475 | **0.010** |
| Log(CS):Species | 1 | 0.000268 | 0.000268 | 0.00214 | 0.1738 | -0.4794 | 0.691 |
| Residuals | 6 | 0.009254 | 0.001542 | 0.07376 |  |  |  |
| Total | 9 | 0.125468 |  |  |  |  |  |

1. Supplementary Table S5: Results of the linear regressions of principal components versus size in the total ontogeny analysis.

**PC1**

Linear regression of *Alligator mississipiensis* PC1 coordinates on Log Centroid Size

Coefficients:

|  | Estimate | Std. Error | t value | Pr(>\|t\|) |
| --- | --- | --- | --- | --- |
| Intercept | -0.14731 | 0.07392 | -1.993 | 0.0865 |
| Slope | 0.24614 | 0.03829 | 6.429 | 0.6531 |

Residual standard error: 0.0275 on 7 degrees of freedom

Multiple R-squared: 0.03052, Adjusted R-squared: -0.108

F-statistic: 0.2203 on 1 and 7 DF, p-value: 0.6531

Linear regression of *Caiman latirostris* PC1 coordinates on Log Centroid Size

Coefficients:

|  | Estimate | Std. Error | t value | Pr(>\|t\|) |
| --- | --- | --- | --- | --- |
| Intercept | -0.003219 | 0.075360 | -0.043 | 0.967 |
| Slope | 0.036730 | 0.022160 | 1.658 | 0.136 |

Residual standard error: 0.02752 on 8 degrees of freedom

Multiple R-squared: 0.2556, Adjusted R-squared: 0.1626

F-statistic: 2.747 on 1 and 8 DF, p-value: 0.136

Linear regression of *Caiman crocodilus* PC1 coordinates on Log Centroid Size

Coefficients:

|  | Estimate | Std. Error | t value | Pr(>\|t\|) |
| --- | --- | --- | --- | --- |
| Intercept | 0.2925 | 0.4222 | 0.693 | 0.614 |
| Slope | 0.1359 | 0.1405 | 0.967 | 0.511 |

Residual standard error: 0.05454 on 1 degrees of freedom

Multiple R-squared: 0.4834, Adjusted R-squared: -0.03326

F-statistic: 0.9356 on 1 and 1 DF, p-value: 0.5106

Linear regression of *Crocodylus niloticus* PC1 coordinates on Log Centroid Size

Coefficients:

|  | Estimate | Std. Error | t value | Pr(>\|t\|) |
| --- | --- | --- | --- | --- |
| Intercept | 0.65297 | 0.11186 | 5.837 | **0.000639** |
| Slope | 0.21312 | 0.03855 | 5.528 | **0.000880** |

Residual standard error: 0.06237 on 7 degrees of freedom

Multiple R-squared: 0.8136, **Adjusted R-squared: 0.787**

F-statistic: 30.56 on 1 and 7 DF, **p-value: 0.0008803**

Linear regression of *Crocodylus porosus* and *Crocodylus siamensis* PC1 coordinates on Log Centroid Size (regrouped as their taxonomic determination is uncertain)

Coefficients:

|  | Estimate | Std. Error | t value | Pr(>\|t\|) |
| --- | --- | --- | --- | --- |
| Intercept | 0.17502 | 0.06005 | 2.914 | 0.100 |
| Slope | 0.07024 | 0.02145 | 3.274 | 0.082 |

Residual standard error: 0.01845 on 2 degrees of freedom

Multiple R-squared: 0.8428, Adjusted R-squared: 0.7642

F-statistic: 10.72 on 1 and 2 DF, p-value: 0.08196

Linear regression of *Mecistops* PC1 coordinates on Log Centroid Size

Coefficients:

|  | Estimate | Std. Error | t value | Pr(>\|t\|) |
| --- | --- | --- | --- | --- |
| Intercept | 0.76048 | 0.15444 | 4.924 | **0.00791** |
| Slope | 0.25515 | 0.05854 | 4.359 | **0.01207** |

Residual standard error: 0.02735 on 4 degrees of freedom

Multiple R-squared: 0.8261, **Adjusted R-squared: 0.7826**

F-statistic: 19 on 1 and 4 DF, **p-value: 0.01207**

Linear regression of *Osteolaemus* PC1 coordinates on Log Centroid Size

Coefficients:

|  | Estimate | Std. Error | t value | Pr(>\|t\|) |
| --- | --- | --- | --- | --- |
| Intercept | 0.23973 | 0.27161 | 0.883 | 0.442 |
| Slope | 0.09186 | 0.09379 | 0.979 | 0.400 |

Residual standard error: 0.05292 on 3 degrees of freedom

Multiple R-squared: 0.2423, Adjusted R-squared: -0.01029

F-statistic: 0.9593 on 1 and 3 DF, p-value: 0.3996

Linear regression of *Gavialis* PC1 coordinates on Log Centroid Size

Coefficients:

|  | Estimate | Std. Error | t value | Pr(>\|t\|) |
| --- | --- | --- | --- | --- |
| Intercept | 0.34997 | 0.09010 | 3.884 | **0.0302** |
| Slope | 0.11606 | 0.03303 | 3.514 | **0.0391** |

Residual standard error: 0.05043 on 3 degrees of freedom

Multiple R-squared: 0.8046, **Adjusted R-squared: 0.7394**

F-statistic: 12.35 on 1 and 3 DF, **p-value: 0.03908**

Linear regression of *Tomistoma* PC1 coordinates on Log Centroid Size

Coefficients:

|  | Estimate | Std. Error | t value | Pr(>\|t\|) |
| --- | --- | --- | --- | --- |
| Intercept | 0.6389 | 0.1610 | 3.968 | **0.0286** |
| Slope | 0.1553 | 0.0547 | 2.839 | 0.0657 |

Residual standard error: 0.04304 on 3 degrees of freedom

Multiple R-squared: 0.7288, Adjusted R-squared: 0.6383

F-statistic: 8.06 on 1 and 3 DF, p-value: 0.0657

**PC2**

Linear regression of *Alligator mississipiensis* PC2 coordinates on Log Centroid Size

Coefficients:

|  | Estimate | Std. Error | t value | Pr(>\|t\|) |
| --- | --- | --- | --- | --- |
| Intercept | 0.78843 | 0.11214 | 7.031 | **0.000206** |
| Slope | 0.23358 | 0.03386 | 6.898 | **0.000232** |

Residual standard error: 0.04172 on 7 degrees of freedom

Multiple R-squared: 0.8718, **Adjusted R-squared: 0.8534**

F-statistic: 47.58 on 1 and 7 DF, **p-value: 0.0002317**

Linear regression of *Caiman latirostris* PC2 coordinates on Log Centroid Size

Coefficients:

|  | Estimate | Std. Error | t value | Pr(>\|t\|) |
| --- | --- | --- | --- | --- |
| Intercept | 0.59459 | 0.09547 | 6.228 | **0.000252** |
| Slope | 0.18763 | 0.02807 | 6.684 | **0.000155** |

Residual standard error: 0.03487 on 8 degrees of freedom

Multiple R-squared: 0.8481, **Adjusted R-squared: 0.8291**

F-statistic: 44.68 on 1 and 8 DF, **p-value: 0.0001553**

Linear regression of *Caiman crocodilus* PC2 coordinates on Log Centroid Size

Coefficients:

|  | Estimate | Std. Error | t value | Pr(>\|t\|) |
| --- | --- | --- | --- | --- |
| Intercept | 0.46260 | 0.27795 | 1.664 | 0.344 |
| Slope | 0.15476 | 0.09247 | 1.674 | 0.343 |

Residual standard error: 0.03591 on 1 degrees of freedom

Multiple R-squared: 0.7369, Adjusted R-squared: 0.4738

F-statistic: 2.801 on 1 and 1 DF, p-value: 0.3429

Linear regression of *Crocodylus niloticus* PC2 coordinates on Log Centroid Size

Coefficients:

|  | Estimate | Std. Error | t value | Pr(>\|t\|) |
| --- | --- | --- | --- | --- |
| Intercept | 0.09106 | 0.08147 | 1.118 | 0.301 |
| Slope | 0.04060 | 0.02808 | 1.446 | 0.191 |

Residual standard error: 0.04542 on 7 degrees of freedom

Multiple R-squared: 0.23, Adjusted R-squared: 0.12

F-statistic: 2.091 on 1 and 7 DF, p-value: 0.1915

Linear regression of *Crocodylus porosus* and *Crocodylus siamensis* PC2 coordinates on Log Centroid Size (regrouped as their taxonomic determination is uncertain)

Coefficients:

|  | Estimate | Std. Error | t value | **Pr(>\|t\|)** |
| --- | --- | --- | --- | --- |
| Intercept | 0.86288 | 0.04732 | 18.24 | **0.00299** |
| Slope | 0.29925 | 0.01690 | 17.70 | **0.00318** |

Residual standard error: 0.01454 on 2 degrees of freedom

Multiple R-squared: 0.9937, **Adjusted R-squared: 0.9905**

F-statistic: 313.5 on 1 and 2 DF, **p-value: 0.003175**

Linear regression of *Mecistops* PC2 coordinates on Log Centroid Size

Coefficients:

|  | Estimate | Std. Error | t value | Pr(>\|t\|) |
| --- | --- | --- | --- | --- |
| Intercept | -0.15057 | 0.14825 | -1.016 | 0.367 |
| Slope | -0.07611 | 0.05619 | -1.355 | 0.247 |

Residual standard error: 0.02626 on 4 degrees of freedom

Multiple R-squared: 0.3145, Adjusted R-squared: 0.1431

F-statistic: 1.835 on 1 and 4 DF, p-value: 0.247

Linear regression of *Osteolaemus* PC2 coordinates on Log Centroid Size

Coefficients:

|  | Estimate | Std. Error | t value | Pr(>\|t\|) |
| --- | --- | --- | --- | --- |
| Intercept | 0.07409 | 0.33866 | 0.219 | 0.841 |
| Slope | 0.01848 | 0.11695 | 0.158 | 0.884 |

Residual standard error: 0.06599 on 3 degrees of freedom

Multiple R-squared: 0.008252, Adjusted R-squared: -0.3223

F-statistic: 0.02496 on 1 and 3 DF, p-value: 0.8845

Linear regression of *Gavialis* PC2 coordinates on Log Centroid Size

Coefficients:

|  | Estimate | Std. Error | t value | **Pr(>\|t\|)** |
| --- | --- | --- | --- | --- |
| Intercept | 0.43936 | 0.08853 | 4.963 | **0.0157** |
| Slope | 0.15482 | 0.03245 | 4.771 | **0.0175** |

Residual standard error: 0.04955 on 3 degrees of freedom

Multiple R-squared: 0.8836, **Adjusted R-squared: 0.8448**

F-statistic: 22.77 on 1 and 3 DF, **p-value: 0.01749**

Linear regression of *Tomistoma* PC2 coordinates on Log Centroid Size

Coefficients:

|  | Estimate | Std. Error | t value | Pr(>\|t\|) |
| --- | --- | --- | --- | --- |
| Intercept | -0.03170 | 0.16597 | -0.191 | 0.861 |
| Slope | 0.02275 | 0.05639 | 0.403 | 0.714 |

Residual standard error: 0.04437 on 3 degrees of freedom

Multiple R-squared: 0.05147, Adjusted R-squared: -0.2647

F-statistic: 0.1628 on 1 and 3 DF, p-value: 0.7136

1. Supplementary Table S6: Ancestral state estimation of ontogenetic trajectory coefficients on PC1 and PC2.

**PC1 Intercept**

| Node (with node number) | Estimation | 95% confidence interval | |
| --- | --- | --- | --- |
|  |  | Min | Max |
| Crocodylia (N10) | 0.2306882 | -0.170884074 | 0.6322604 |
| Longirostres (N11) | 0.4060227 | 0.121018494 | 0.6910269 |
| Crocodylidae (N12) | 0.4429130 | 0.227820939 | 0.6580051 |
| Crocodylus (N13) | 0.4239233 | 0.242356238 | 0.6054904 |
| Osteolaeminae (N14) | 0.4662132 | 0.263493295 | 0.6689332 |
| Gavialidae (N15) | 0.4674298 | 0.237120950 | 0.6977387 |
| Alligatoridae (N16) | 0.1464073 | -0.222926483 | 0.5157411 |
| Caimaninae (N17) | 0.1447366 | -0.009416755 | 0.2988899 |

**PC1 Slope**

| Node (with node number) | Estimation | 95% confidence interval | |
| --- | --- | --- | --- |
|  |  | Min | Max |
| Crocodylia (N10) | 0.09198746 | -0.02808564 | 0.2120606 |
| Longirostres (N11) | 0.13249764 | 0.04727925 | 0.2177160 |
| Crocodylidae (N12) | 0.15034108 | 0.08602693 | 0.2146552 |
| Crocodylus (N13) | 0.14465661 | 0.09036670 | 0.1989465 |
| Osteolaeminae (N14) | 0.15977898 | 0.09916420 | 0.2203938 |
| Gavialidae (N15) | 0.13470896 | 0.06584488 | 0.2035730 |
| Alligatoridae (N16) | 0.07251479 | -0.03791879 | 0.1829484 |
| Caimaninae (N17) | 0.08540270 | 0.03930969 | 0.1314957 |

**PC2 Intercept**

| Node (with node number) | Estimation | 95% confidence interval | |
| --- | --- | --- | --- |
|  |  | Min | Max |
| Crocodylia (N10) | 0.4358197 | -0.06080275 | 0.9324422 |
| Longirostres (N11) | 0.2672966 | -0.08516679 | 0.6197599 |
| Crocodylidae (N12) | 0.2202079 | -0.04579550 | 0.4862112 |
| Crocodylus (N13) | 0.3888032 | 0.16426008 | 0.6133463 |
| Osteolaeminae (N14) | 0.1149141 | -0.13578873 | 0.3656169 |
| Gavialidae (N15) | 0.2232216 | -0.06160023 | 0.5080435 |
| Alligatoridae (N16) | 0.5168264 | 0.06007305 | 0.9735798 |
| Caimaninae (N17) | 0.5278244 | 0.33718372 | 0.7184652 |

**PC2 Slope**

| Node (with node number) | Estimation | 95% confidence interval | |
| --- | --- | --- | --- |
|  |  | Min | Max |
| Crocodylia (N10) | 0.14006650 | -0.027005284 | 0.3071383 |
| Longirostres (N11) | 0.09277671 | -0.025797624 | 0.2113510 |
| Crocodylidae (N12) | 0.07209346 | -0.017394343 | 0.1615813 |
| Crocodylus (N13) | 0.13633257 | 0.060792656 | 0.2118725 |
| Osteolaeminae (N14) | 0.03098098 | -0.053359467 | 0.1153214 |
| Gavialidae (N15) | 0.09000720 | -0.005811447 | 0.1858259 |
| Alligatoridae (N16) | 0.16279803 | 0.009138852 | 0.3164572 |
| Caimaninae (N17) | 0.17064599 | 0.106511394 | 0.2347806 |

1. Supplementary Table S7: Results of the linear regression of Principal components versus size in the subclade-only analyses.

**Alligatoridae-only PCA**

Linear regression of *Alligator mississipiensis* PC1 coordinates on Log Centroid Size

Coefficients:

|  | Estimate | Std. Error | t value | Pr(>\|t\|) |
| --- | --- | --- | --- | --- |
| Intercept | 0.84680 | 0.12679 | 6.679 | **0.000283** |
| Slope | 0.24614 | 0.03829 | 6.429 | **0.000357** |

Residual standard error: 0.04717 on 7 degrees of freedom

Multiple R-squared: 0.8552, **Adjusted R-squared: 0.8345**

F-statistic: 41.33 on 1 and 7 DF, **p-value: 0.0003573**

Linear regression of *Caiman latirostris* PC1 coordinates on Log Centroid Size

Coefficients:

|  | Estimate | Std. Error | t value | Pr(>\|t\|) |
| --- | --- | --- | --- | --- |
| Intercept | 0.71066 | 0.07671 | 9.264 | **1.50e-05** |
| Slope | 0.22297 | 0.02256 | 9.885 | **9.26e-06** |

Residual standard error: 0.02802 on 8 degrees of freedom

Multiple R-squared: 0.9243, **Adjusted R-squared: 0.9149**

F-statistic: 97.71 on 1 and 8 DF, **p-value: 9.256e-06**

Linear regression of *Caiman crocodilus* PC1 coordinates on Log Centroid Size

Coefficients:

|  | Estimate | Std. Error | t value | Pr(>\|t\|) |
| --- | --- | --- | --- | --- |
| Intercept | 0.65896 | 0.15972 | 4.126 | 0.151 |
| Slope | 0.21507 | 0.05314 | 4.047 | 0.154 |

Residual standard error: 0.02063 on 1 degrees of freedom

Multiple R-squared: 0.9425, Adjusted R-squared: 0.8849

F-statistic: 16.38 on 1 and 1 DF, p-value: 0.1542

Linear regression of *Alligator mississipiensis* PC2 coordinates on Log Centroid Size

Coefficients:

|  | Estimate | Std. Error | t value | Pr(>\|t\|) |
| --- | --- | --- | --- | --- |
| Intercept | 0.04324 | 0.06160 | 0.702 | 0.5054 |
| Slope | 0.03893 | 0.01860 | 2.093 | 0.0747 |

Residual standard error: 0.02292 on 7 degrees of freedom

Multiple R-squared: 0.3849, Adjusted R-squared: 0.297

F-statistic: 4.379 on 1 and 7 DF, p-value: 0.07467

Linear regression of *Caiman latirostris* PC2 coordinates on Log Centroid Size

Coefficients:

|  | Estimate | Std. Error | t value | Pr(>\|t\|) |
| --- | --- | --- | --- | --- |
| Intercept | 0.35419 | 0.03920 | 9.035 | **1.8e-05** |
| Slope | 0.08953 | 0.01153 | 7.767 | **5.4e-05** |

Residual standard error: 0.01432 on 8 degrees of freedom

Multiple R-squared: 0.8829, **Adjusted R-squared: 0.8683**

F-statistic: 60.33 on 1 and 8 DF, **p-value: 5.397e-05**

Linear regression of *Caiman crocodilus* PC2 coordinates on Log Centroid Size

Coefficients:

|  | Estimate | Std. Error | t value | Pr(>\|t\|) |
| --- | --- | --- | --- | --- |
| Intercept | 0.267834 | 0.024192 | 11.07 | 0.0573 |
| Slope | 0.074851 | 0.008049 | 9.30 | 0.0682 |

Residual standard error: 0.003125 on 1 degrees of freedom

Multiple R-squared: 0.9886, Adjusted R-squared: 0.9771

F-statistic: 86.48 on 1 and 1 DF, p-value: 0.06819

**Crocodylidae-only PCA**

Linear regression of *Crocodylus niloticus* PC1 coordinates on Log Centroid Size

Coefficients:

|  | Estimate | Std. Error | t value | Pr(>\|t\|) |
| --- | --- | --- | --- | --- |
| Intercept | -0.67451 | 0.12331 | -5.470 | **0.00156** |
| Slope | -0.23632 | 0.04263 | -5.544 | **0.00145** |

Residual standard error: 0.06875 on 6 degrees of freedom

Multiple R-squared: 0.8367, **Adjusted R-squared: 0.8094**

F-statistic: 30.73 on 1 and 6 DF, **p-value: 0.001454**

Linear regression of *Crocodylus porosus* and *Crocodylus siamensis* PC1 coordinates on Log Centroid Size (regrouped as their taxonomic determination is uncertain)

Coefficients:

|  | Estimate | Std. Error | t value | Pr(>\|t\|) |
| --- | --- | --- | --- | --- |
| Intercept | -0.15872 | 0.05541 | -2.865 | 0.1033 |
| Slope | -0.08554 | 0.01979 | -4.322 | **0.0496** |

Residual standard error: 0.01702 on 2 degrees of freedom

Multiple R-squared: 0.9033, **Adjusted R-squared: 0.8549**

F-statistic: 18.68 on 1 and 2 DF, **p-value: 0.04959**

Linear regression of *Mecistops* PC1 coordinates on Log Centroid Size

Coefficients:

|  | Estimate | Std. Error | t value | Pr(>\|t\|) |
| --- | --- | --- | --- | --- |
| Intercept | -0.89166 | 0.18869 | -4.725 | **0.00913** |
| Slope | -0.33175 | 0.07152 | -4.639 | **0.00974** |

Residual standard error: 0.03342 on 4 degrees of freedom

Multiple R-squared: 0.8432, **Adjusted R-squared: 0.8041**

F-statistic: 21.52 on 1 and 4 DF, **p-value: 0.009744**

Linear regression of *Osteolaemus* PC1 coordinates on Log Centroid Size

Coefficients:

|  | Estimate | Std. Error | t value | Pr(>\|t\|) |
| --- | --- | --- | --- | --- |
| Intercept | -0.19963 | 0.24296 | -0.822 | 0.471 |
| Slope | -0.09326 | 0.08390 | -1.112 | 0.347 |

Residual standard error: 0.04734 on 3 degrees of freedom

Multiple R-squared: 0.2917, Adjusted R-squared: 0.05564

F-statistic: 1.236 on 1 and 3 DF, p-value: 0.3474

Linear regression of *Crocodylus niloticus* PC2 coordinates on Log Centroid Size

Coefficients:

|  | Estimate | Std. Error | t value | Pr(>\|t\|) |
| --- | --- | --- | --- | --- |
| Intercept | 0.05656 | 0.08369 | 0.676 | 0.524 |
| Slope | 0.03190 | 0.02893 | 1.103 | 0.312 |

Residual standard error: 0.04666 on 6 degrees of freedom

Multiple R-squared: 0.1685, Adjusted R-squared: 0.02988

F-statistic: 1.216 on 1 and 6 DF, p-value: 0.3125

Linear regression of *Crocodylus porosus* and *Crocodylus siamensis* PC2 coordinates on Log Centroid Size (regrouped as their taxonomic determination is uncertain)

Coefficients:

|  | Estimate | Std. Error | t value | Pr(>\|t\|) |
| --- | --- | --- | --- | --- |
| Intercept | 0.48443 | 0.02985 | 16.23 | **0.00378** |
| Slope | 0.17686 | 0.01066 | 16.58 | **0.00362** |

Residual standard error: 0.009172 on 2 degrees of freedom

Multiple R-squared: 0.9928, **Adjusted R-squared: 0.9892**

F-statistic: 275 on 1 and 2 DF, **p-value: 0.003617**

Linear regression of *Mecistops* PC2 coordinates on Log Centroid Size

Coefficients:

|  | Estimate | Std. Error | t value | Pr(>\|t\|) |
| --- | --- | --- | --- | --- |
| Intercept | 0.19958 | 0.14862 | 1.343 | 0.250 |
| Slope | 0.08995 | 0.05633 | 1.597 | 0.186 |

Residual standard error: 0.02632 on 4 degrees of freedom

Multiple R-squared: 0.3893, Adjusted R-squared: 0.2366

F-statistic: 2.55 on 1 and 4 DF, p-value: 0.1855

Linear regression of *Osteolaemus* PC2 coordinates on Log Centroid Size

Coefficients:

|  | Estimate | Std. Error | t value | Pr(>\|t\|) |
| --- | --- | --- | --- | --- |
| Intercept | 0.22102 | 0.31841 | 0.694 | 0.559 |
| Slope | 0.04617 | 0.10889 | 0.424 | 0.713 |

Residual standard error: 0.06008 on 2 degrees of freedom

Multiple R-squared: 0.08248, Adjusted R-squared: -0.3763

F-statistic: 0.1798 on 1 and 2 DF, p-value: 0.7128

**Gavialidae-only PCA**

Linear regression of *Gavialis* PC1 coordinates on Log Centroid Size

Coefficients:

|  | Estimate | Std. Error | t value | Pr(>\|t\|) |
| --- | --- | --- | --- | --- |
| Intercept | -0.39435 | 0.12306 | -3.205 | **0.0492** |
| Slope | -0.10528 | 0.04511 | -2.334 | 0.1018 |

Residual standard error: 0.06887 on 3 degrees of freedom

Multiple R-squared: 0.6449, Adjusted R-squared: 0.5265

F-statistic: 5.448 on 1 and 3 DF, p-value: 0.1018

Linear regression of *Tomistoma* PC1 coordinates on Log Centroid Size

Coefficients:

|  | Estimate | Std. Error | t value | Pr(>\|t\|) |
| --- | --- | --- | --- | --- |
| Intercept | 0.19514 | 0.15103 | 1.292 | 0.287 |
| Slope | 0.02699 | 0.05131 | 0.526 | 0.635 |

Residual standard error: 0.04037 on 3 degrees of freedom

Multiple R-squared: 0.08446, Adjusted R-squared: -0.2207

F-statistic: 0.2767 on 1 and 3 DF, p-value: 0.6353

Linear regression of *Gavialis* PC2 coordinates on Log Centroid Size

Coefficients:

|  | Estimate | Std. Error | t value | Pr(>\|t\|) |
| --- | --- | --- | --- | --- |
| Intercept | 0.45776 | 0.06453 | 7.094 | **0.00576** |
| Slope | 0.18499 | 0.02365 | 7.822 | **0.00435** |

Residual standard error: 0.03611 on 3 degrees of freedom

Multiple R-squared: 0.9533, **Adjusted R-squared: 0.9377**

F-statistic: 61.18 on 1 and 3 DF, **p-value: 0.004351**

Linear regression of *Tomistoma* PC2 coordinates on Log Centroid Size

Coefficients:

|  | Estimate | Std. Error | t value | Pr(>\|t\|) |
| --- | --- | --- | --- | --- |
| Intercept | 0.63987 | 0.15786 | 4.053 | **0.0271** |
| Slope | 0.20840 | 0.05363 | 3.886 | **0.0302** |

Residual standard error: 0.0422 on 3 degrees of freedom

Multiple R-squared: 0.8342, **Adjusted R-squared: 0.779**

F-statistic: 15.1 on 1 and 3 DF, **p-value: 0.03021**
